# A Review on Applications of Artificial Intelligence (AI) in Monoclonal Antibody (mAb) Manufacturing

**DOI:** 10.3390/molecules31183313

**Published:** 2026-09-18

**Authors:** Fawad Abidi, Dimitrios I. Gerogiorgis

**Affiliations:** Institute for Materials and Processes (IMP), School of Engineering, University of Edinburgh, Edinburgh EH9 3FB, UK

**Keywords:** artificial intelligence, machine learning, deep learning, neural networks, mAbs

## Abstract

This review paper offers a comprehensive exploration of the many Artificial Intelligence (AI) applications in monoclonal antibody (mAb) manufacturing, covering a wide spectrum of topics, including upstream and downstream processes, process control, optimisation, product property monitoring and regulatory compliance. We present several case studies showcasing the predictive modelling capabilities of AI and ML algorithms for cell culture optimisation, media formulation, and bioreactor control, highlighting their potential to enhance cell growth, productivity, and product quality. Furthermore, we explore its potential in process monitoring, fault detection, and real-time decision-making, towards improved process robustness and reduced production costs.

## 1. Introduction

Artificial Intelligence (AI) shows exceptional potential to revolutionise many aspects of monoclonal antibody (mAb) manufacturing, encompassing many application areas toward improved efficiency, cost-effectiveness, quality control and process optimisation [1]. Because AI can rapidly analyse large datasets collected during manufacturing processes and identify patterns or correlations that humans may overlook, it can optimise operating process parameters (temperature, pH, stirring) for higher mAb yield and purity.

AI algorithms can predict the outcome of different process variables and identify critical parameters affecting mAb production. By leveraging historical data, AI can provide insights into the likelihood of process failure or suboptimal performance, enabling manufacturers to take proactive measures. Quality control is another important area that can benefit from AI, by analysing real-time data from key sources (e.g., in-line sensors), to detect deviations or anomalies in the manufacturing process, provide early warnings of potential quality issues, enabling timely interventions for product quality [2].

AI implementations allow for tracking multiple process parameters simultaneously, supporting the vision of real-time feedback control signal provision to plant operators. Automatic adjustment of process parameters can occur on the basis of predefined criteria, ensuring consistent and reproducible mAb production. AI can guide decision-making by analysing multidimensional legacy datasets, considering a multitude of pivotal factors (e.g., product specifications, manufacturing costs, regulatory frames), assisting technical managers to decide on process modifications, raw materials, and production scheduling.

Beyond biopharma process operations, drug formulation optimisation can also be performed via AI/ML algorithms, via analysing in-house data to identify optimal excipient choices for maximum mAb stability, solubility, product performance, and shelf life. Moreover, AI can analyse sensor data and legacy records to predict equipment failures and determine preventive maintenance needs, whose proactive implementation can thus reduce OpEx, minimise plant downtime and ensure continuous mAb production.

### 1.1. AI Methodologies

AI can be classified into distinct categories based on the machine’s capacity to use previous data/experiences to predict future outcomes/decisions: the most frequently used AI tools include machine learning (ML), artificial neural networks (ANNs), machine vision (MV) and deep learning (DL). All these AI methodologies have been applied to tackle demanding medicine and biopharma industry problems in the past two decades.

ML is one of the AI tools where machines are not explicitly programmed to perform specific tasks; instead, they automatically learn and improve from experience. The algorithms use computational methods to directly *learn* from data. There are various machine learning algorithms, e.g., unsupervised, supervised, and reinforcement learning.

#### 1.1.1. Supervised Learning (SL)

Supervised learning is useful when data relevant to a desired prediction is available: therein, previous input-output data is used to predict new outputs based on new inputs. The SL model can make predictions based on evidence, and in the presence of uncertainty; SL algorithms receive a known input dataset and known resulting (output) responses, and then train a model to generate reasonable predictions of output responses for new inputs.

Support Vector Machines (SVM) is an exemplary SL algorithm, offering an exciting approach for model development, which can also be used for estimation and prediction.

#### 1.1.2. Unsupervised Learning (UL)

Unsupervised learning targets the discovery of patterns, structures, or relationships in unlabeled, unstructured data without predefined target outputs. Widespread methods comprise *clustering* (grouping similar data) and *dimensionality reduction* (simplifying data), often used for e.g., customer segmentation, anomaly detection, and data visualisation.

#### 1.1.3. Reinforcement Learning (RL)

In reinforcement learning, the machine is instructed to learn by engagement, hence providing better, more reliable results as more experience is gained and accumulated. Thus, RL can maximise a cumulative reward by recording actions via a trial-and-error approach in a set environment. A great RL advantage is that it can be applied with little or even no historical data: unlike other ML methods, it does not require prior information.

#### 1.1.4. Artificial Neural Networks (ANNs) and Deep Learning (DL)

ANNs aim to capture nonlinear patterns in data via structured layers of parameters. Every ANN has inputs, a hidden layer with parameters, and one or more output layers. Deep learning denotes an ANN with hidden layers, encompassing various architectures; DL techniques require large datasets and great computing power (many GPUs) for best performance, since they tune many parameters postulated within elaborate frameworks.

The focus of this review paper is a detailed overview of ANN case studies and their key benefits for mAb manufacturing; we note that such implementations require careful validation, regulatory compliance, and integration with existing processes/workflows. Harnessing AI power thus offers higher productivity, great mAb quality and cost savings.

## 2. Model Development

**Figure 1:**
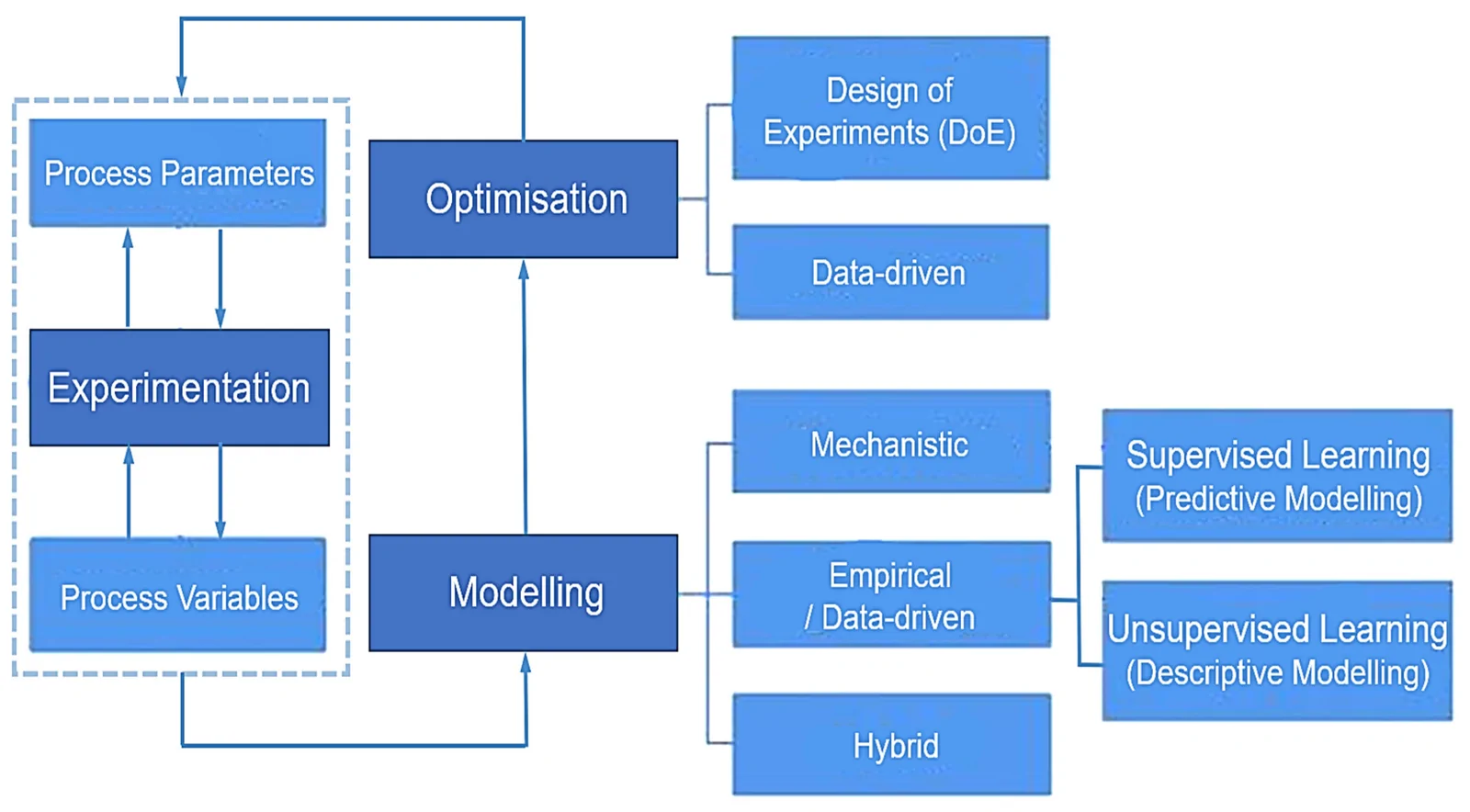
An overview of approaches for biopharma modelling and optimisation, drawn from [3].

Bioprocess modelling plays an important role in biotherapeutics manufacturing, its goal being to estimate optimal production conditions, reduce delays and minimise risks. Bioprocessing modelling tools are broadly classified under: (i) *Mechanistic modelling*, and (ii) *Empirical modelling* (Figure 1). Mechanistic models are maps with rigorous mathematical representations of complex mAb production biosystems (structure and functions), whose usage requires extensive process knowledge and parameterisation.

The complex nature of mAbs manufacturing bioprocesses induces convoluted reaction kinetics, hence globally hampering kinetic model fidelity and parameterisation. Rolandi analysed advanced biopharma modelling via first-principles approaches, emphasising a need for model life-cycle frameworks and sustainable model usage [4].

Recent AΙ developments (especially ML and ANN) mitigate the said challenge and offer an alternative to first-principles bottlenecks, thus facilitating bioprocess modelling. Empirical (especially data-driven) modelling does not mandate a prerequisite of profound mAb manufacturing process knowledge: it relies on a lighter set of assumptions about underlying relationships among system variables, and models can predict outputs from input variables on the basis of fitting against a training dataset, under set assumptions.

### 2.1. ANN Models

**Figure 2:**
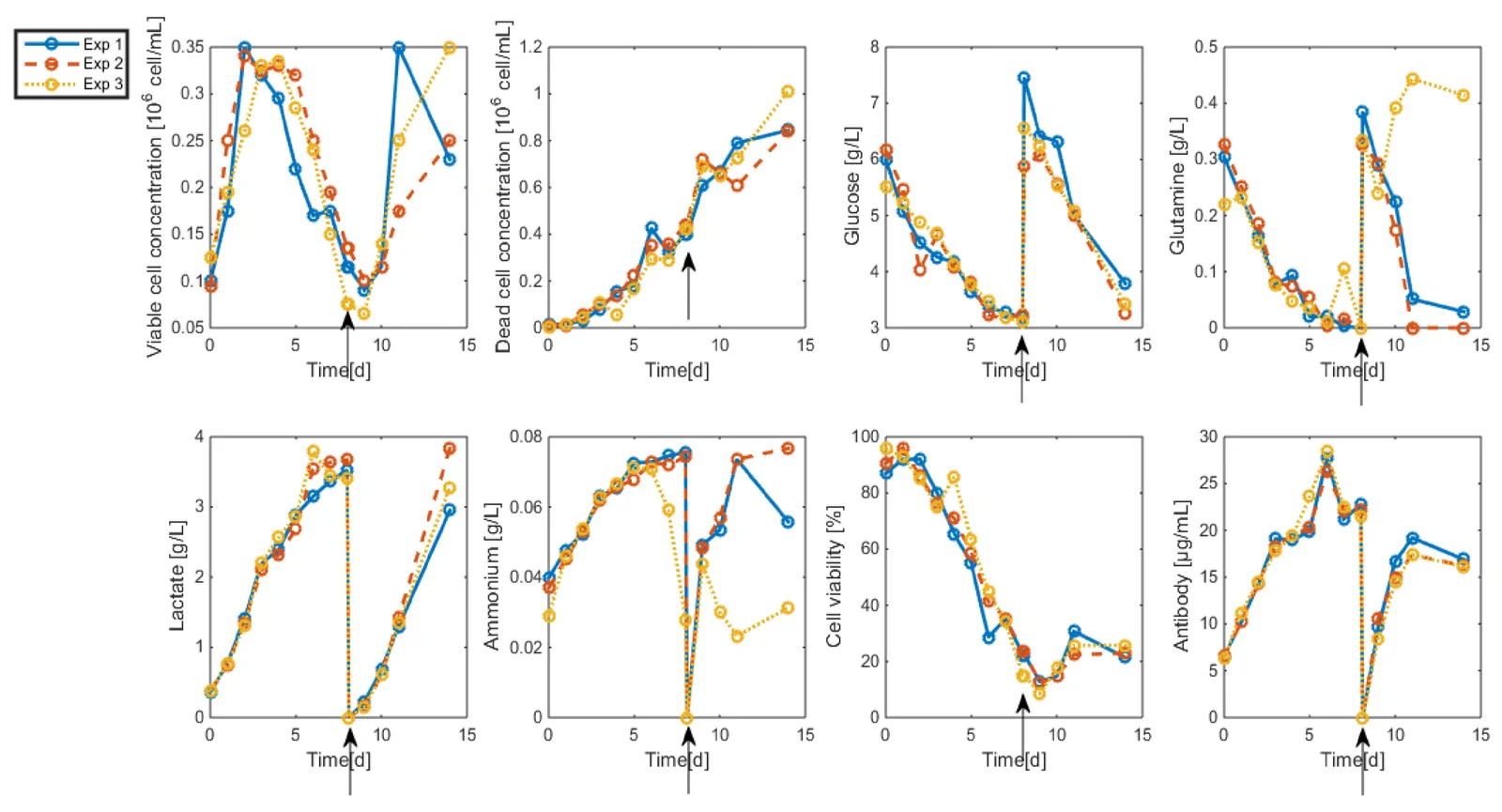
Experimental datasets for sequential batch culture using HB1 hybridoma cells [5].

**Figure 3:**
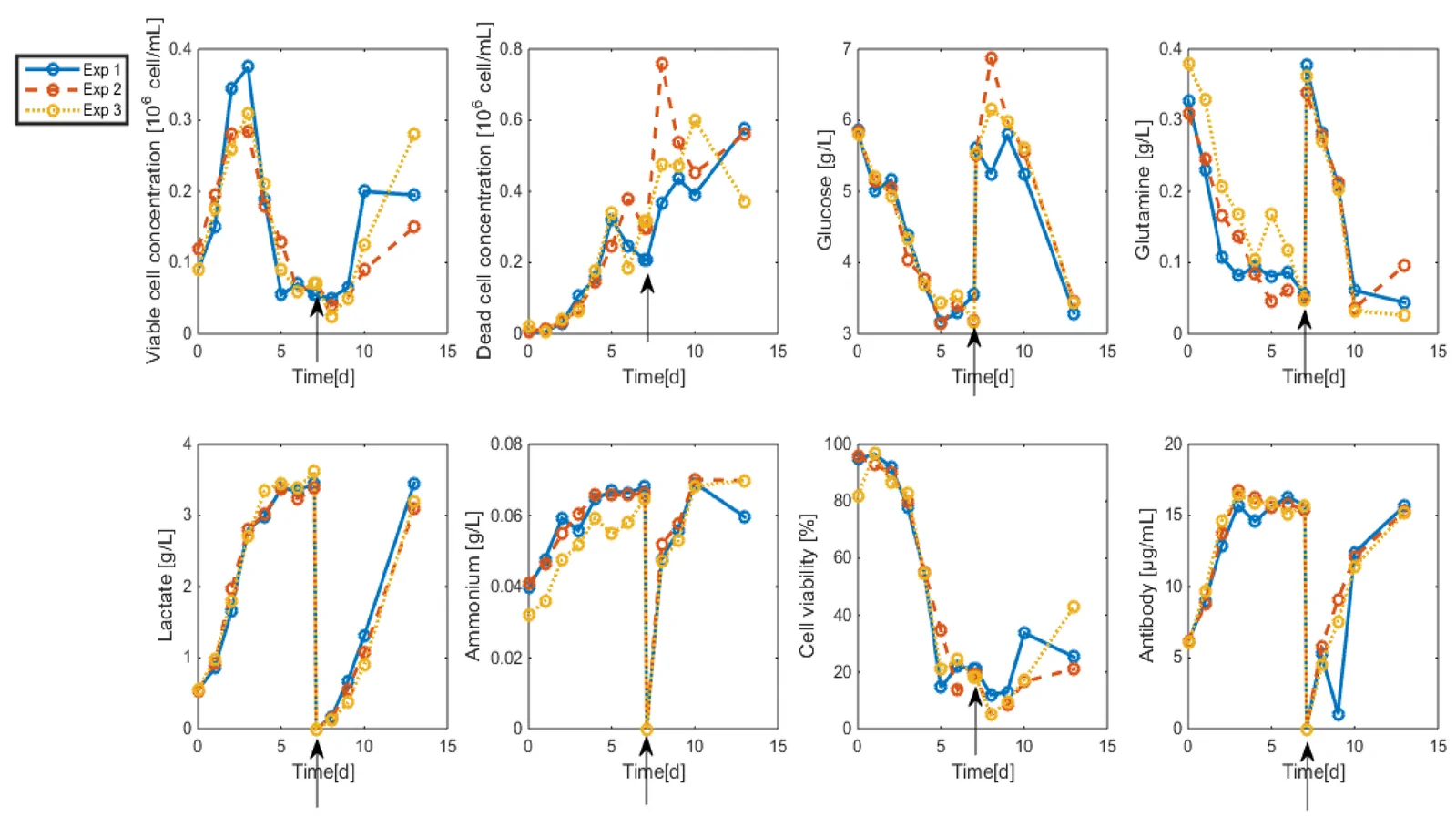
Experimental datasets for sequential batch culture using HB2 hybridoma cells [5].

Dewasme et al. (2017) published a mAb production model, employing six sequential suspended hybridoma batch cell cultures using two hybridoma strains, HB1 and HB2 [5]. The configuration kept biomass in the bioreactor, with products (lactate, ammonia, mAbs) withdrawn. Glucose and glutamine concentrations ranged between 6 and 7 and 0.3–0.4 g/L^−1^, respectively, and the initial biomass concentration of the first batch was at 0.1 × 10^6^ cells/mL^−1^. Cell culture duration was approx. 15 days; culture media renewal occurred after 7 days. Experimental measurements were obtained once daily, with data illustrated in Figure 2 and Figure 3. Cell growth started to decline after 4 days of batch, returning to the initial value at day 7. With fresh culture medium added at this specific time point, culture viability rises again.

Maximum Likelihood Principal Component Analysis (MLPCA) is used with this data to reduce dimensionality, yielding a matrix *ρ* of MLPCA components as a subspace basis:

(1) $$\rho =\left(\begin{array}{ccc} -0.0074 & \;\;\;0.0317 & \;\;\;0.4314\\ -0.0173 & -0.0045 & -0.3108\\ \;\;\;0.1366 & -0.5955 & -0.6581\\ \;\;\;0.0169 & -0.0404 & -0.0561\\ -0.1389 & 0.7778 & -0.5300\\ -0.9805 & -0.1940 & -0.0153\\ . & . & . \end{array}\right)$$

Glucose and glutamine stoichiometric coefficients come from (1); the reaction scheme is:

(2) $$\;\mathrm{Substrate}\;\mathrm{oxidation}:\;\;\;k_{31}G+\;k_{41}Gn\;\overset{\varphi }{\to }X+\;k_{61}mAb$$

(3) $$\;\mathrm{Substrate}\;\mathrm{surplus}\;(\mathrm{to}\;\mathrm{by}\text{-}\mathrm{product}):\;\;k_{32}G+k_{42}Gn\;\overset{\varphi }{\to }X+k_{52}L$$

(4) $$\;\mathrm{Biomass}\;\mathrm{death}:\;X\;\overset{\varphi }{\to }X_{d}+k_{63}mAb$$

Ammonium formation is ignored to achieve a smaller reaction scheme (fewer reactions).

The dynamic reaction model based on mass balances is obtained as an ODE system:

(5) $$\frac{dX}{dt}=\left(\varphi_{1}+\varphi_{2}-\varphi_{3}\right)X$$

(6) $$\;\frac{{dX}_{d}}{dt}=\varphi_{3}X$$

(7) $$\frac{dG}{dt}=-k_{31}\varphi_{1}-k_{32}\varphi_{2}$$

(8) $$\;\;\frac{dGn}{dt}=-k_{41}\varphi_{1}-k_{42}\varphi_{2}$$

(9) $$\;\frac{dL}{dt}=k_{52}\varphi_{2}$$

(10) $$\;\;\frac{d\left[mAb\right]}{dt}=k_{61}\varphi_{1}+k_{63}\varphi_{3}$$

Dewasme et al. propose a viable strategy decoupling stoichiometry identification from reaction kinetics, thus achieving a first estimate of the stoichiometric parameters [2]. Biopharmaceutical processes are extremely complex and require many model parameters.

Model parameterisation for the ODE systems of Equations (5)–(10) was performed via MATLAB^®^ R2025a *fmincon* (constrained minimisation) algorithm, with box constraints used to limit the search space, and typically comprises three successive phases. The first considers the MLPCA stoichiometry estimates and a guess vector for reaction kinetic parameters; the next are initialised with parameter values from previous minimisations.

**Figure 4:**
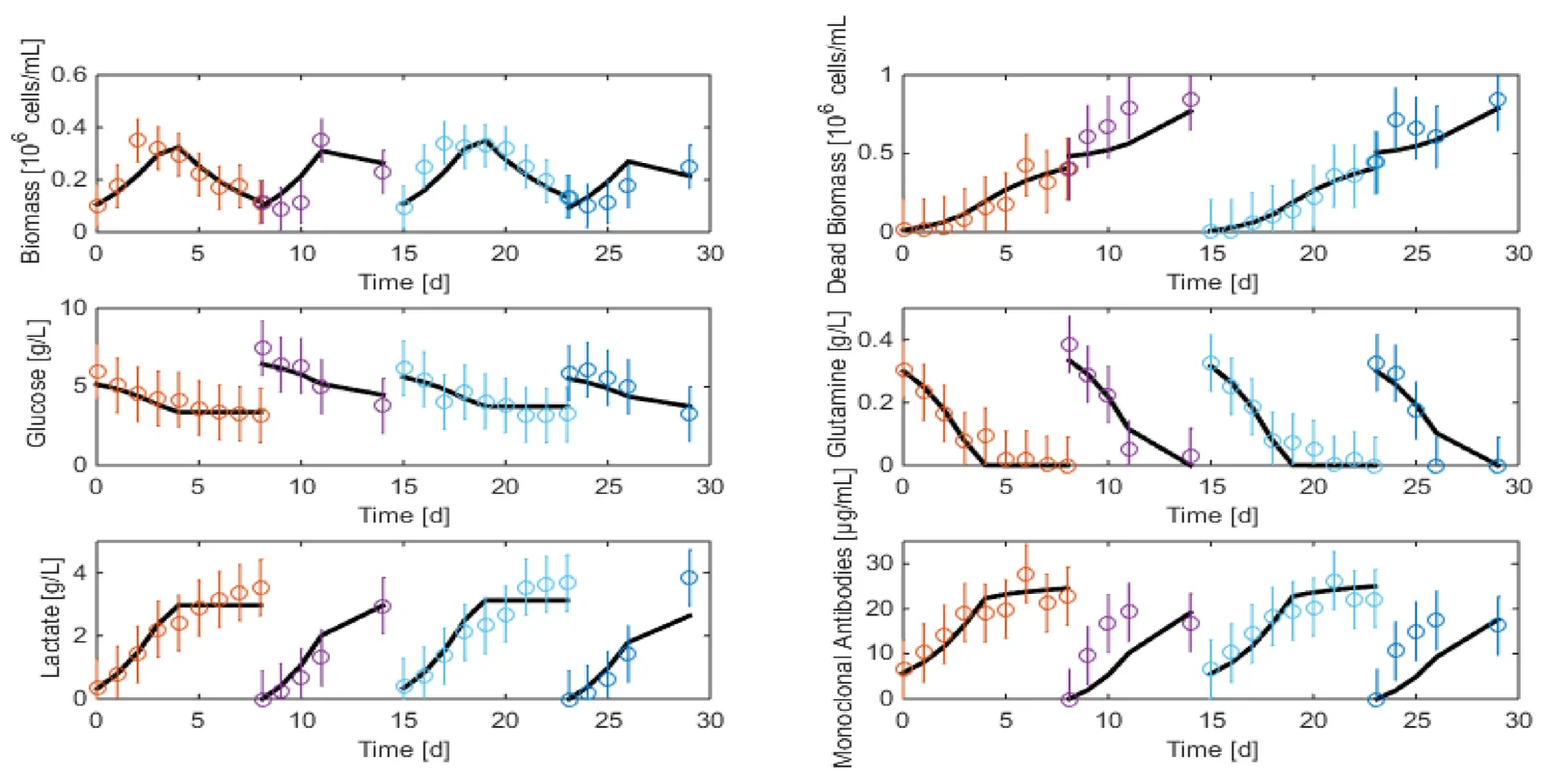
Direct validation of the derived MLPCA model vs. exptl. data for the HB1 strain [5].

Figure 4 presents direct model validation results: coloured circles and error bars (at 99% confidence) denote experiments, and solid black lines are MLPCA model predictions, for a total of six observables (i.e., live and dead biomass, glucose, glutamine, lactate, mAb). The model performs well, but with clear mAb discrepancy after medium renewal (day 8).

Dewasme et al. (2023) later proposed another data-driven strategy for fast dynamic bioprocess modelling with minimal complexity and a minimum number of reactions [6]. MLPCA is used to infer a reduced reaction scheme from a 25-state mammalian cell culture database, and a multi-stage nonlinear model predictive control (MSNMPC) algorithm is used to tackle kinetic structural uncertainty (and then also pursue robust process control).

**Figure 5:**
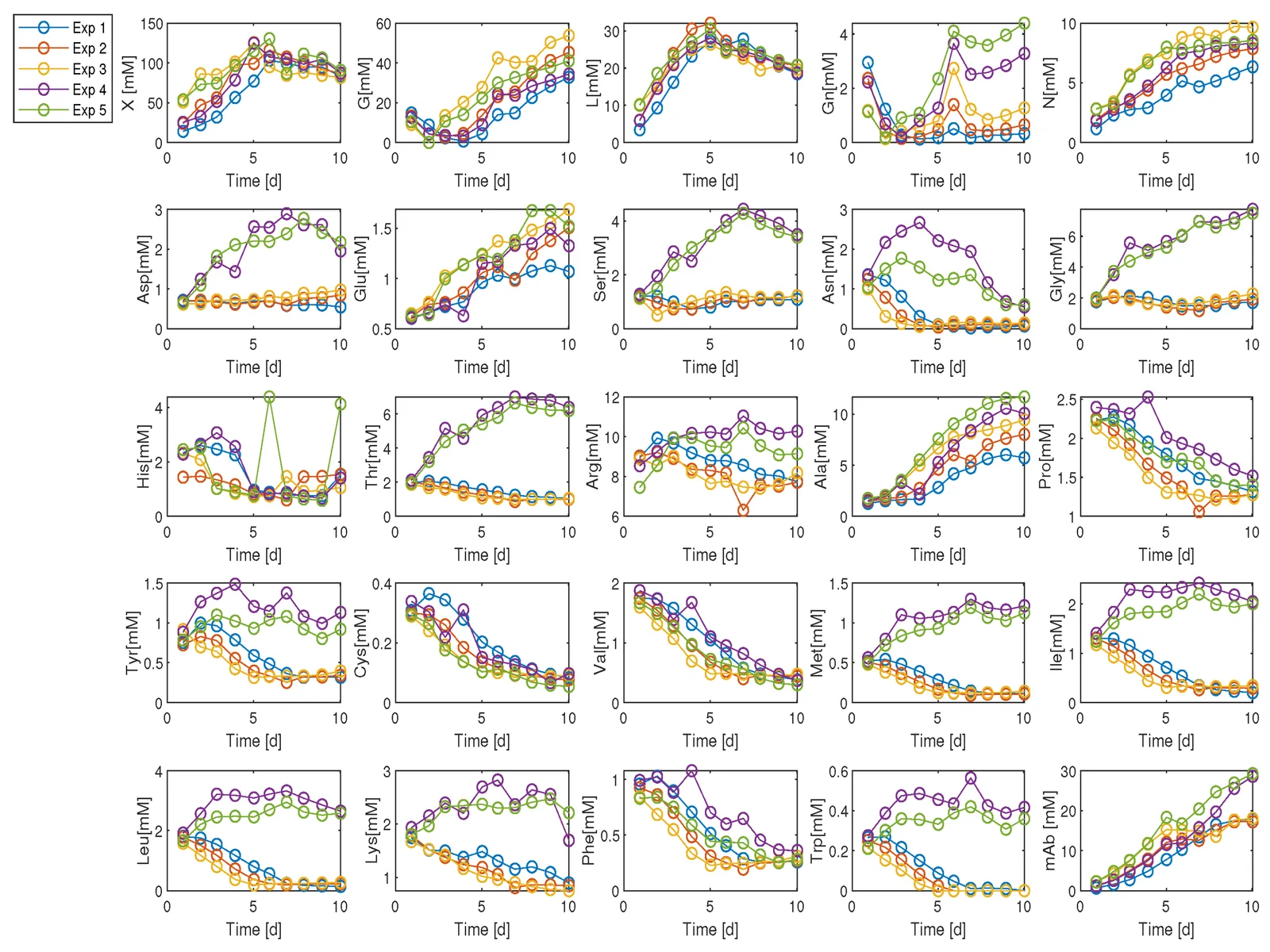
Experiments with low (1–3) and high (4–5) amino acid levels in culture medium [6].

**Figure 6:**
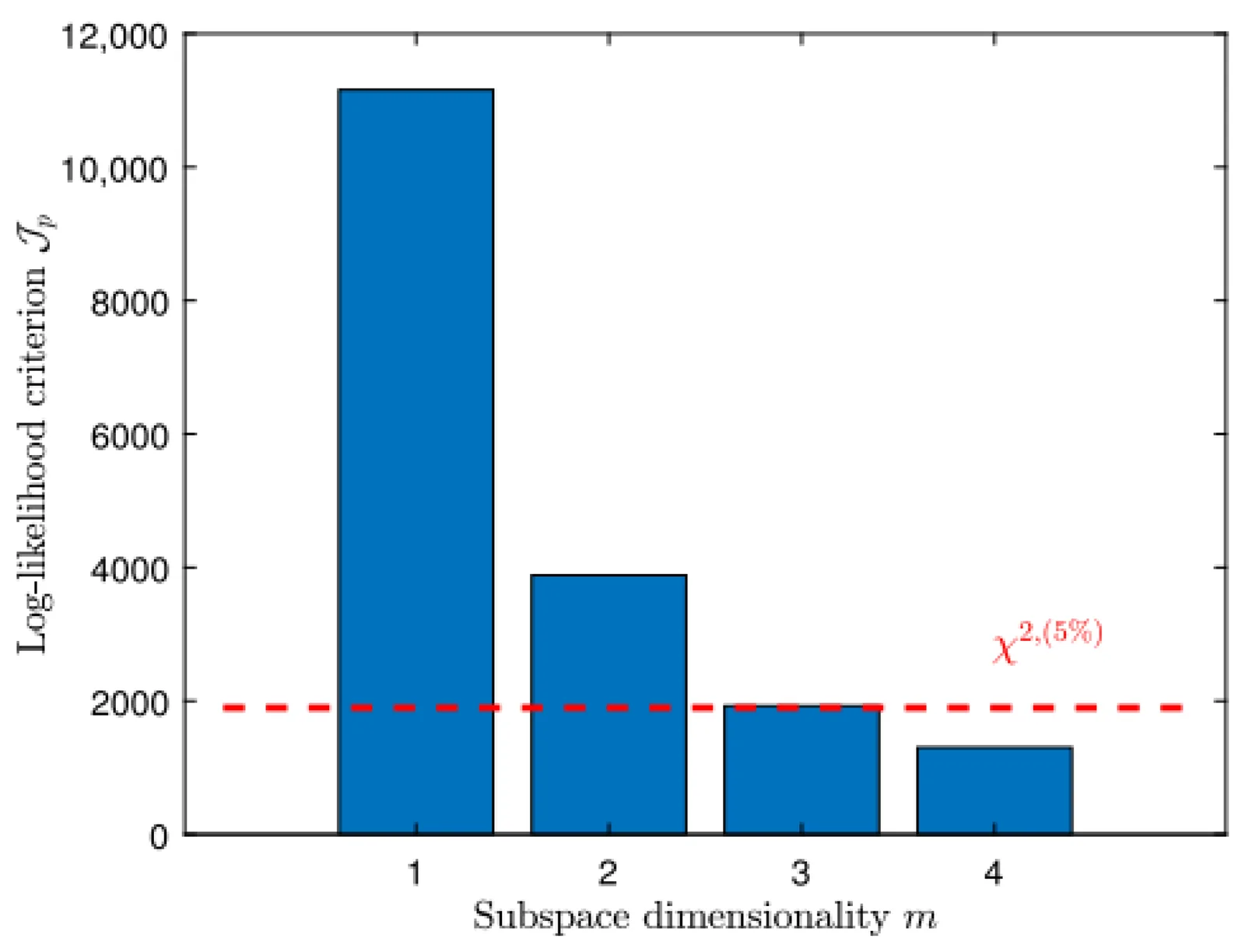
Log-likelihood (*J_P_*) of an m-dimensional subspace (relative noise variance: 10%) [6].

Larger datasets are used therein (Figure 4 and Figure 5, [6]) vs. an early study (Figure 2 and Figure 3, [5]), employing a richer knowledge basis of five (5) experiments and 25 signals for each, with a total of five (3 low, 2 high) amino acid concentration levels in the culture medium.

Figure 6 thus justifies that a 3-dimensional subspace (three principal components) suffices for describing selected data (Expts. 1 + 4), via a log-likelihood criterion (*J_P_*) vs. the MLPCA subspace dimensionality; a dashed line denotes a *χ*^2^ distribution quantile of 5%.

Equation (11) shows a biologically consistent stoichiometric matrix constructed via a linear transformation ($\hat{K}=\rho G$) from MLPCA and *χ*^2^ test-derived basis *ρ* of Equation (1). All three columns of $\hat{K}$ are normalised with respect to biomass concentration; the second features non-positive biomass, glucose and glutamine coefficients, and the third has a zero ammonium coefficient (the 25 row labels denote metabolite, amino acid and mAb, cf. [6]).

(11) $$\hat{K}=\rho G=\left(\begin{array}{ccc} 1 & -1 & 1\\ -0.493 & -0.151 & -2.017\\ 0.266 & -0.443 & -0.153\\ -0.139 & 0 & -0.466\\ 0.074 & -0.106 & 0\\ 0.008 & -0.041 & -0.069\\ 0.0085 & -0.013 & -0.001\\ 0.014 & -0.084 & -0.151\\ 0.003 & -0.099 & -0.222\\ 0.023 & -0.126 & -0.221\\ -0.010 & 0.014 & -0.003\\ 0.012 & -0.105 & -0.207\\ 0.005 & -0.013 & -0.012\\ 0.064 & -0.105 & -0.033\\ -0.006 & 0.004 & -0.011\\ -0.002 & -0.015 & -0.042\\ -0.002 & 0.003 & -0.001\\ -0.011 & 0.011 & -0.009\\ 0.001 & -0.021 & -0.047\\ -0.001 & -0.038 & -0.093\\ -0.002 & -0.059 & -0.147\\ -0.001 & -0.021 & -0.054\\ -0.005 & 0.001 & -0.015\\ -0.001 & -0.010 & -0.025\\ 0.156 & -0.191 & 0.073 \end{array}\right)$$

This MLPCA model formulation includes many previously [5] neglected by-products (alanine, proline, aspartate, asparagine, etc.), and Table 1 summarises model parameters.

**Figure 7:**
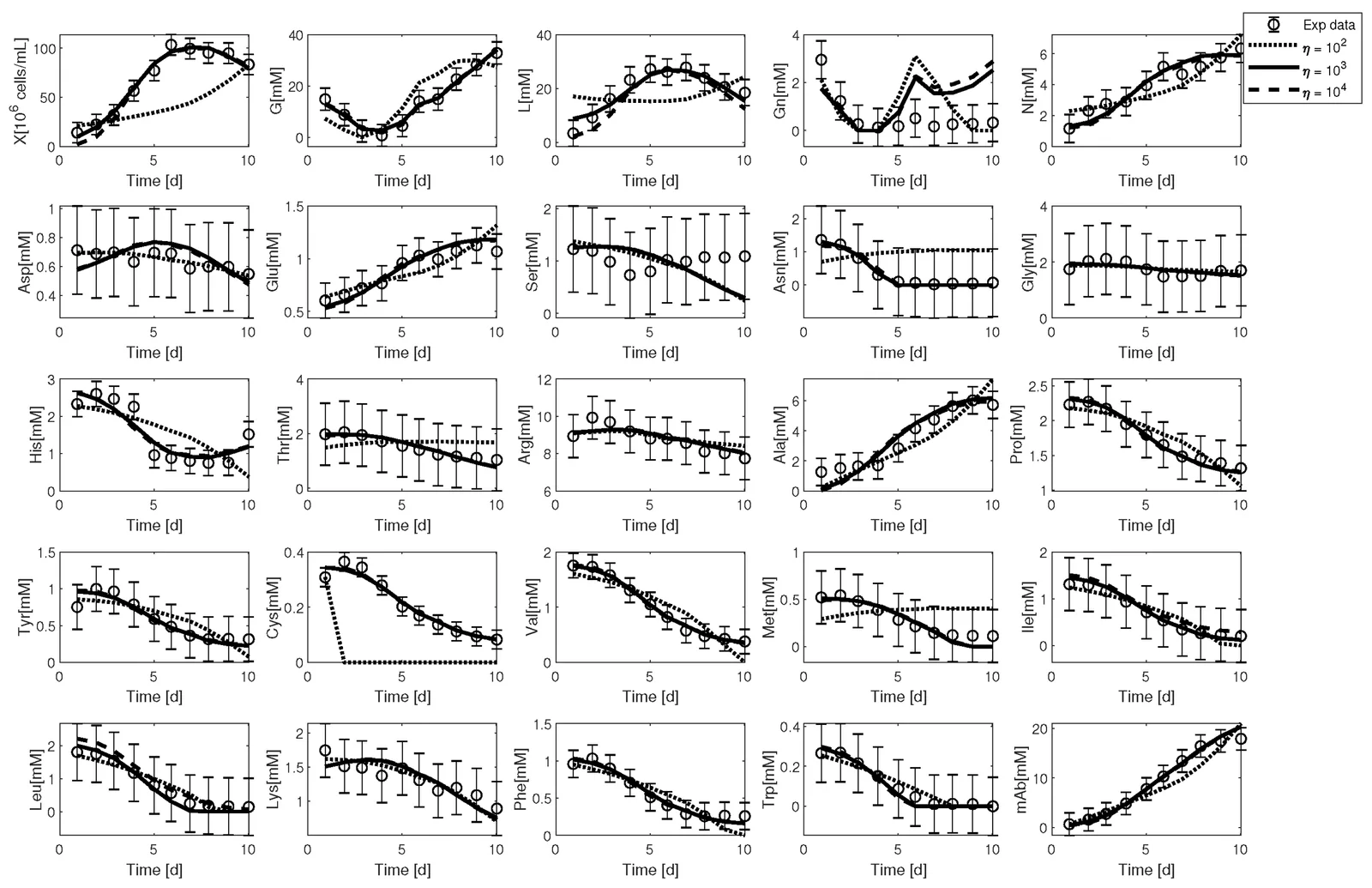
Direct validation of the MLPCA model for three distinct *η* levels vs. Expt. 1 [6].

**Figure 8:**
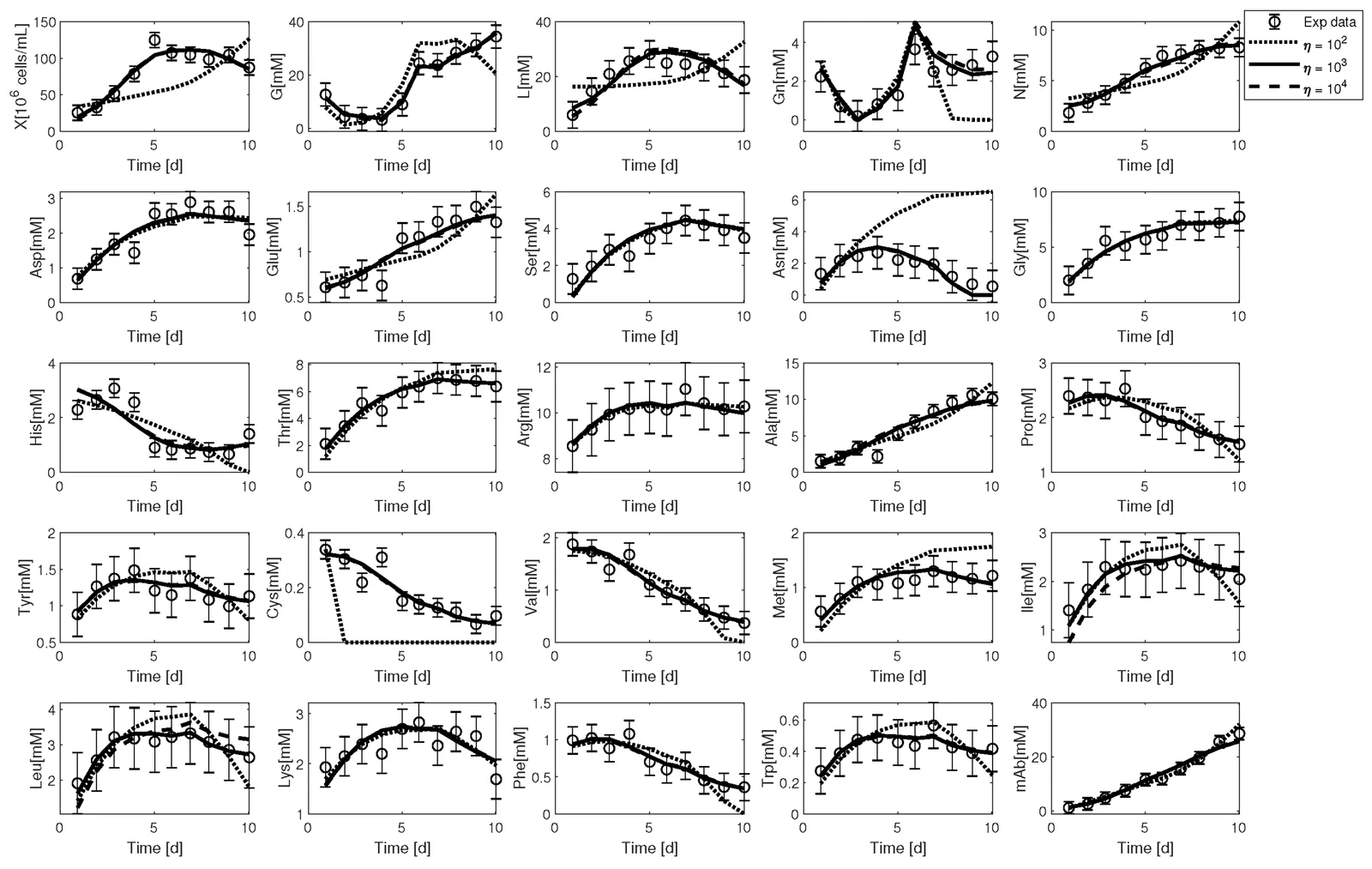
Direct validation of the MLPCA model for three distinct *η* levels vs. Expt. 2 [6].

Direct validation vs. Experiments 1–2 (two culture media) is presented in Figure 7 and Figure 8. Cross-validation was also completed, where only initial conditions were identified, with model parameters set to direct validation values. The other 3 fed-batch Experiments 3–5 (2 with low, 1 with high amino acid concentrations) were used to compute a fit metric, *J*_ML_. Table 2 lists fitting cost function (*J*_ML_) residuals for all cross-validation experiments.

A recent study by Kotidis et al. (2020) employed an ANN-based data-driven model to predict protein glycosylation in mAb manufacturing processes [7], for which rigorous modelling requires accurate estimation of many protein-specific kinetic parameters, as well as prior quantification of enzyme and transport protein levels in the Golgi membrane. Their ANN model describing protein glycosylation was applied for a case study including four recombinant glycoproteins, two mAbs and two CHO-produced fusion proteins [7].

**Figure 9:**
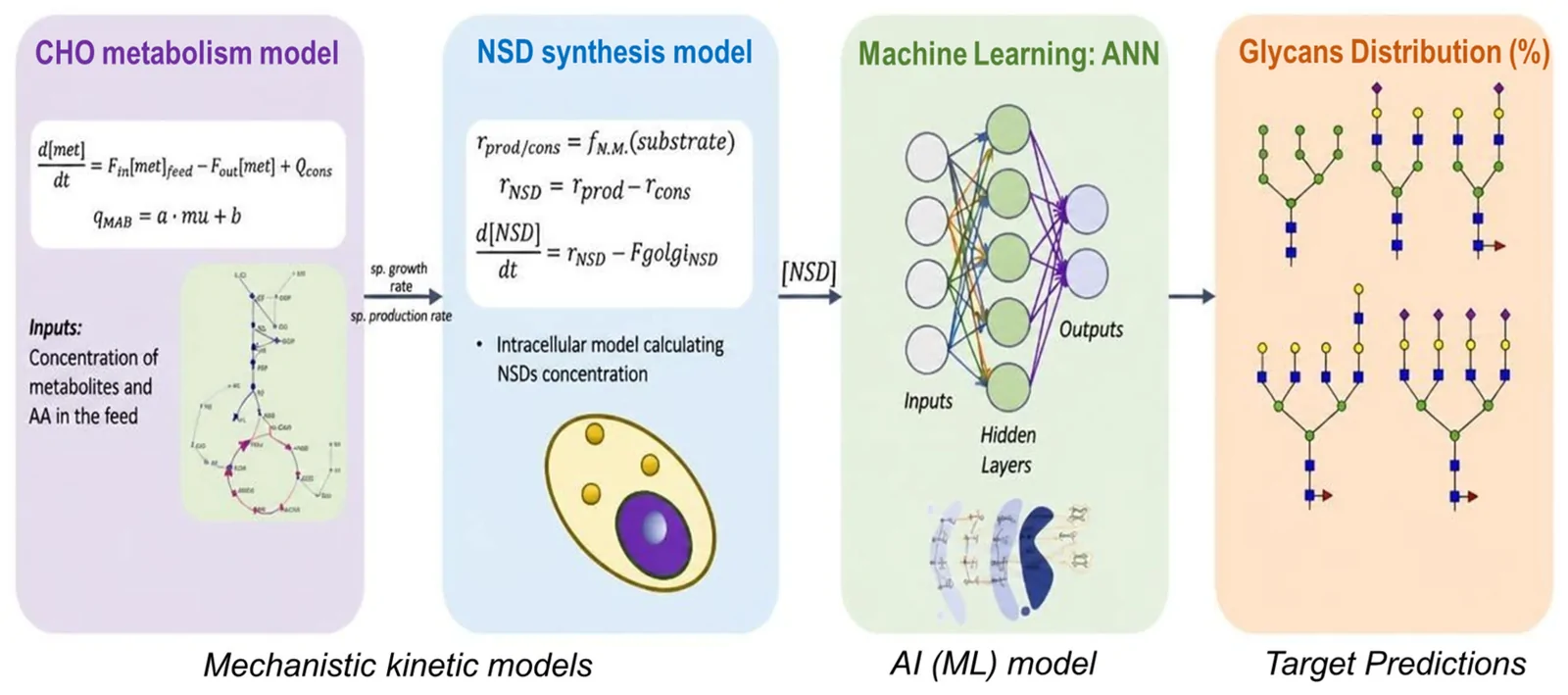
HyGlycoM combines kinetic models with an ANN for glycosylation; adapted from [7].

The glycosylation process is initiated in the endoplasmic reticulum and continues in the Golgi complex: it greatly depends on intra-Golgi membrane glycosylation enzymes and nucleotide sugar donors (NSDs) levels, controlled by nucleotide sugar transporters (NSTs) in the Golgi apparatus. The complex biological phenomena (glycosylation enzyme and NST level variations) governing protein glycosylation and NSD synthesis cannot be captured by Monod-type kinetic models. The proposed data-driven ANN model of [7,8] is aimed at their description using highly nonlinear relationships between nucleotides and NSDs (process inputs) and glycoform (recombinant protein) distribution (process output), circumventing NSD transport modelling challenges if informative training datasets exist. The HyGlycoM tool combines CHO cell metabolism, NSD and mAb synthesis (Figure 9).

**Figure 10:**
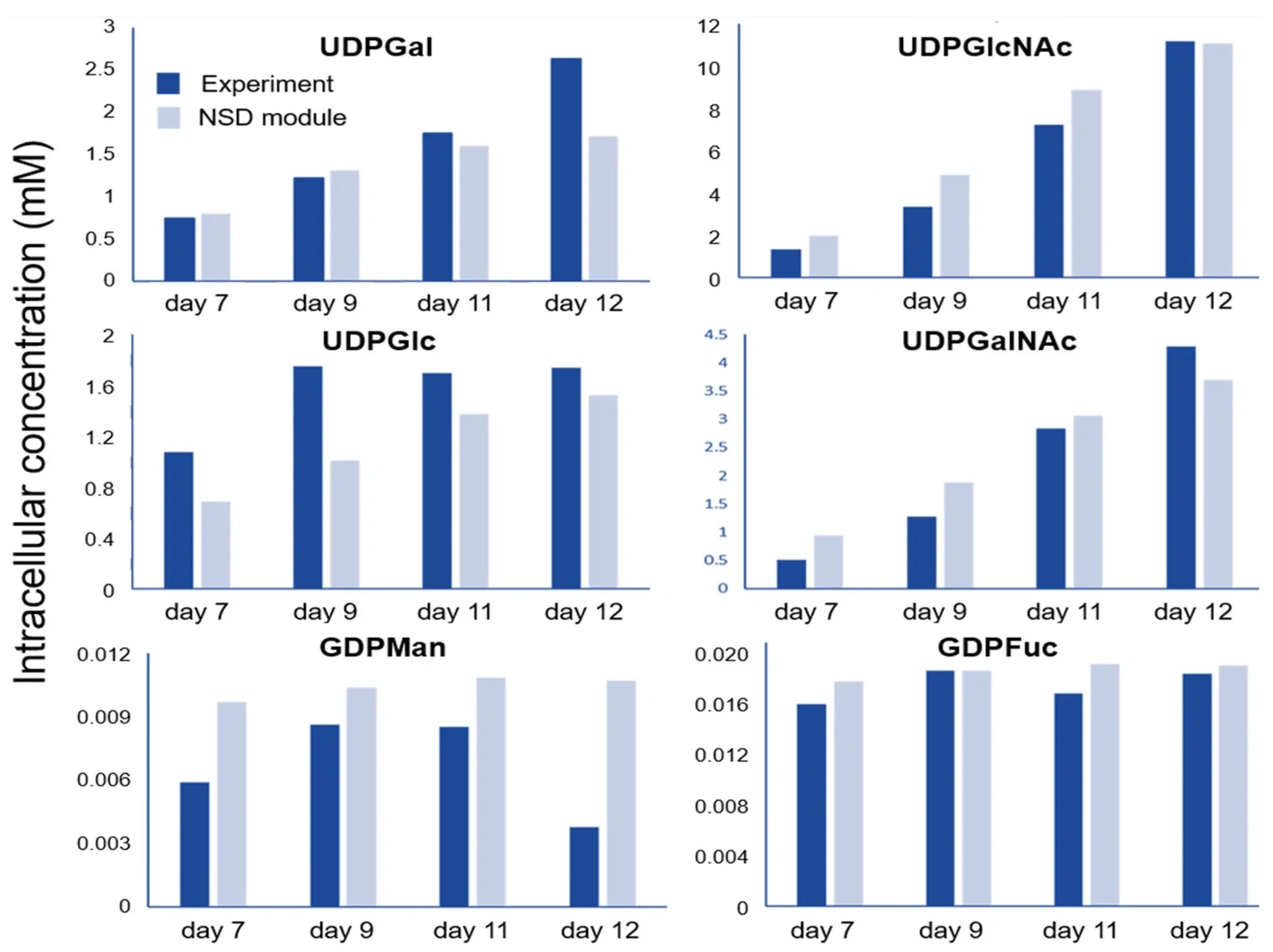
HyGlycoM model NSD estimates vs. exptl. data for 6 nucleotide sugars, data from [7].

Figure 10 shows that the ANN model can greatly suppress kinetic model inaccuracies, which affect nucleotide sugar estimation, featuring high predictive potential and solid estimates; for some, accuracy deteriorates close to the end of runs (days 11–12).

### 2.2. Hybrid Modelling

**Figure 11:**
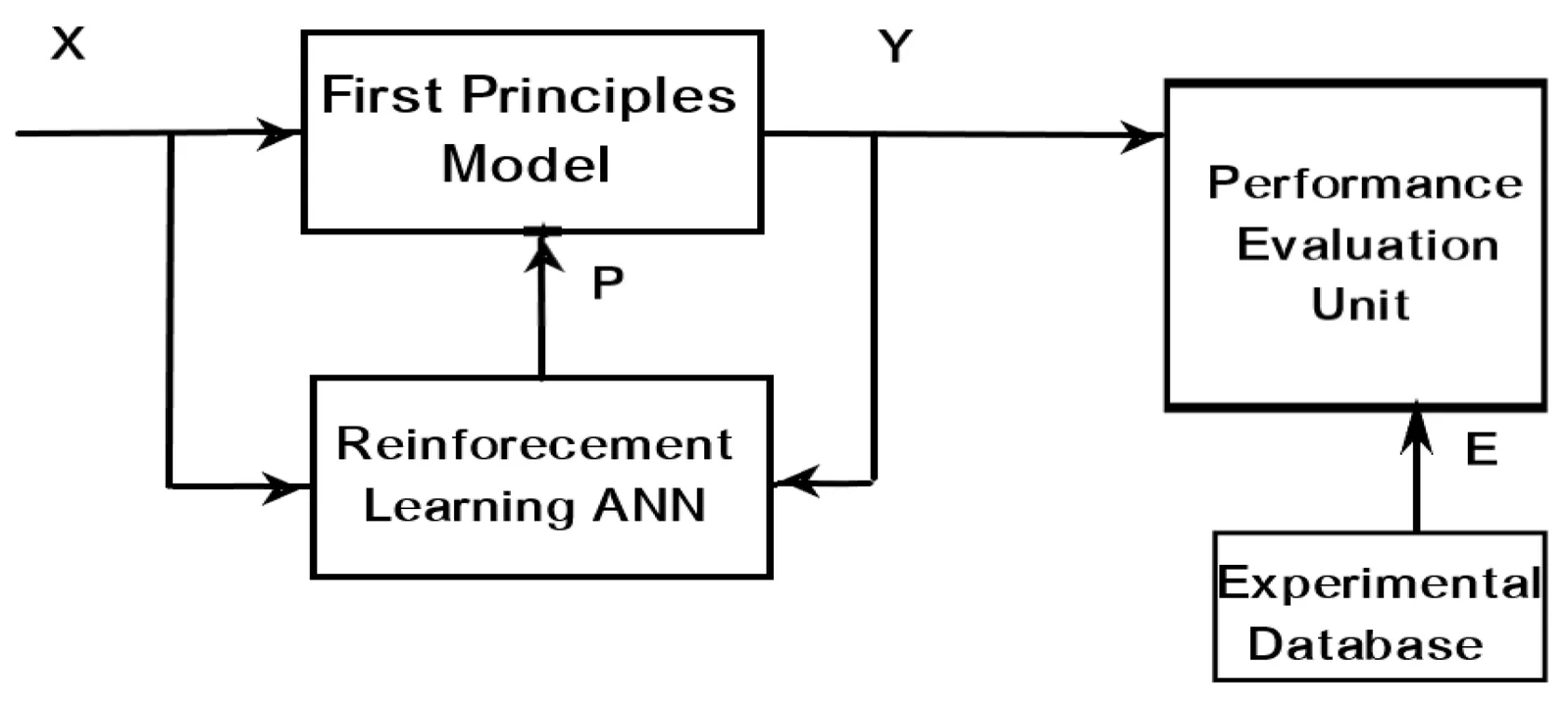
Hybrid model architecture combining first principles, ANN and experimental data [13].

Hybrid models combine more than one system description to boost fidelity [9,10,11,12]. Fu and Barford (1996) pioneered a hybrid system embedding rigorous cell metabolism model (first-principles mass and energy balances combined with reaction kinetics) [13] into an AI framework which used an RL ANN for iterative parameterisation (Figure 11).

**Figure 12:**
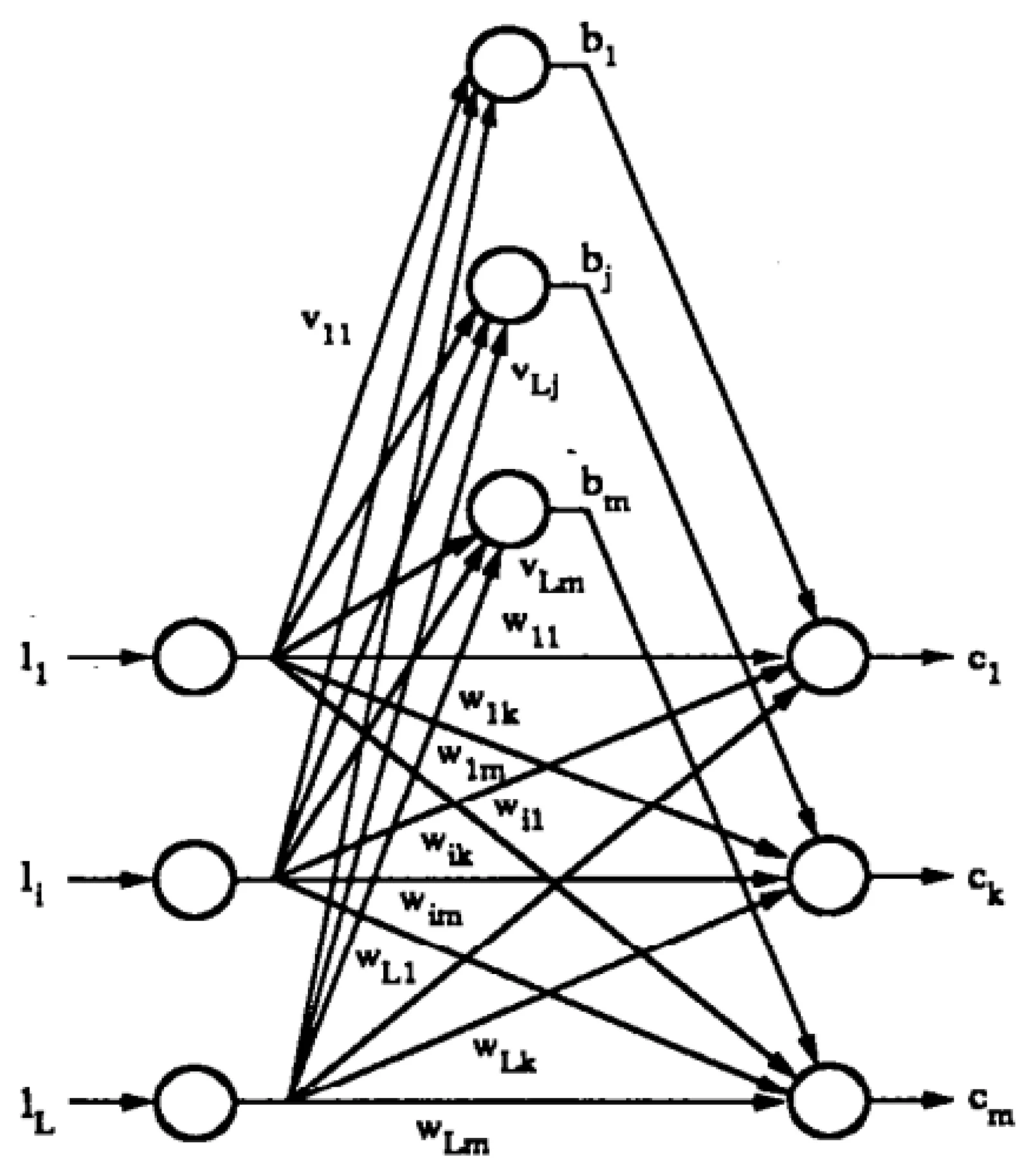
The detailed RL ANN architecture implemented in [13] as first proposed in [14].

The RL ANN block is trained via input (X) and output (Y) datasets and implemented in Fortran to yield model parameters P, which are nonlinear state functions (Figure 12). The 11-step algorithm comprises ANN training, weight updating, internal reinforcement and a performance evaluation unit encoding biochemistry knowledge to decide if the previous P vector is suitable or not vs. the error between predictions Y and actual data E; hence, key hybridoma cell cultivation variables can be kept or altered, respectively [13,14].

A comparative study of its predictive potential was performed vs. experimental data, for the *first-principles model* (solely based on hybridoma growth kinetics for mAb production) and the *hybrid model* (encompassing both reaction kinetics and the ANN as above). The former involves detailed stoichiometry and cell metabolism energetics (45 variables and 145 parameters) and can simulate all key states (specific growth rates, cell densities, macromolecular composition, substrates, lactic and amino acid profiles, and mAb titres). The latter, conversely, relies on the RL ANN structure by Quantrille and Liu (1991), in which first-layer outputs are fed in both the second and third (output) layer [14].

**Figure 13:**
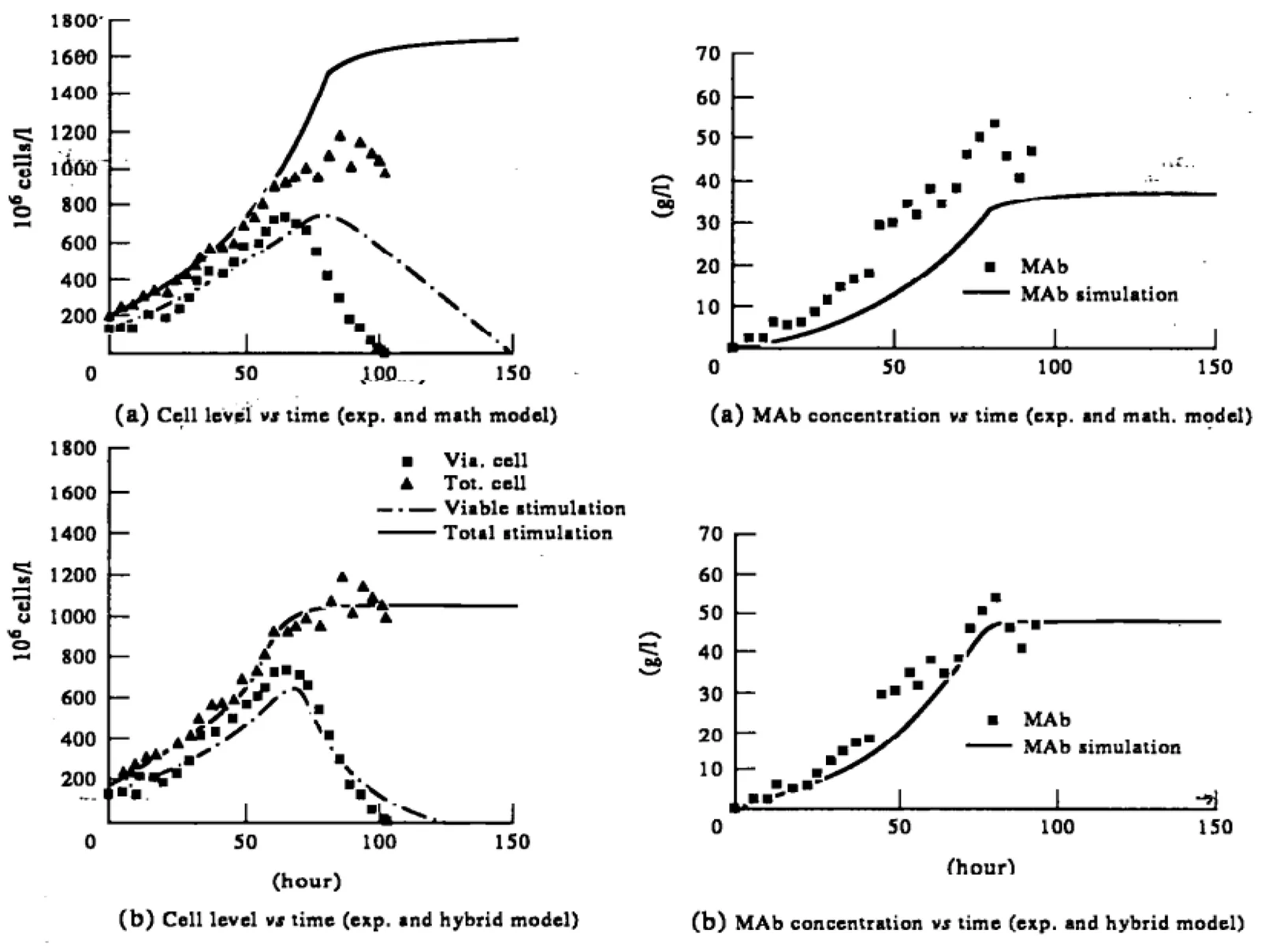
Predictions of (**a**) first-principles and (**b**) hybrid model: cell density and mAb [13].

**Figure 14:**
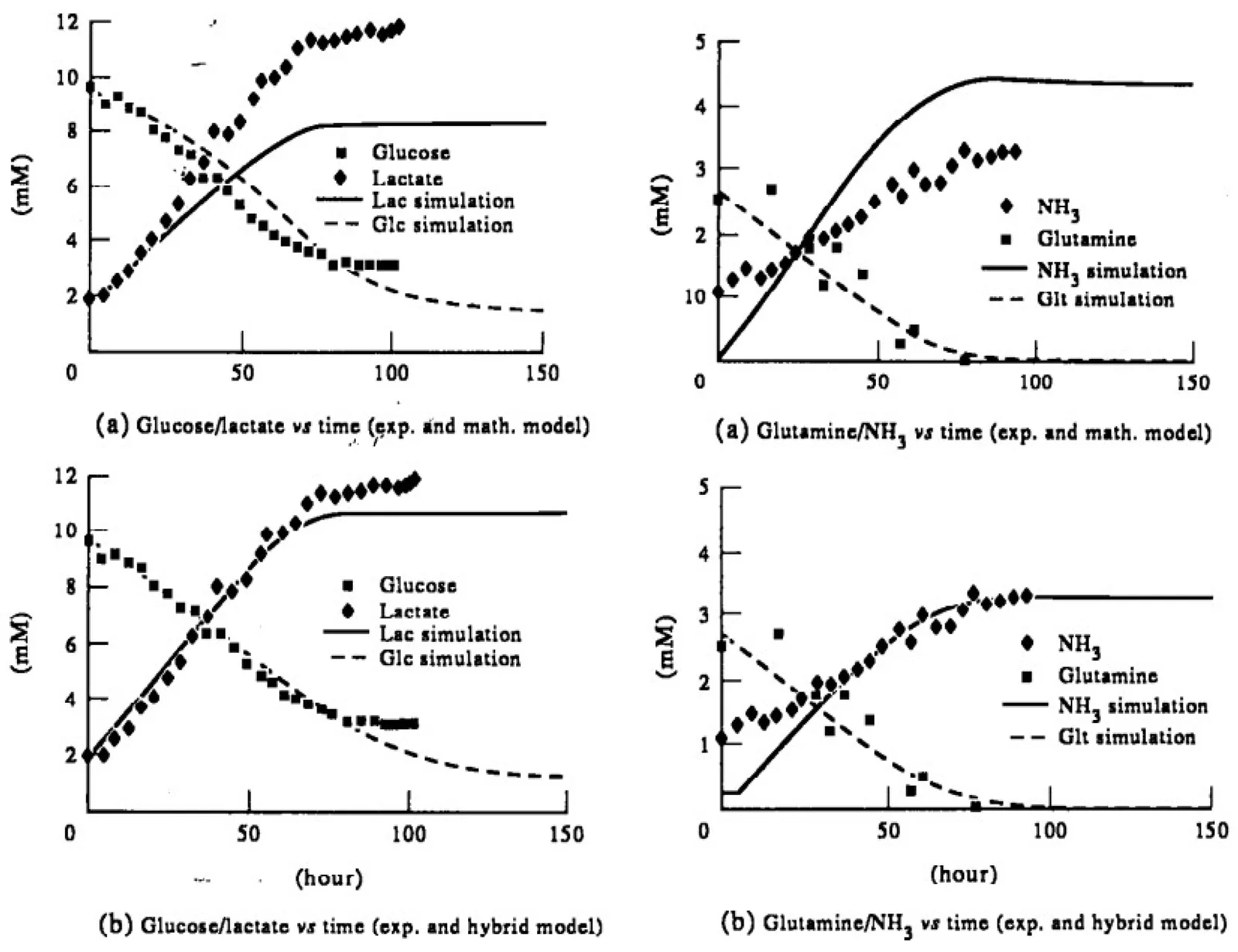
Predictions of (**a**) first-principles and (**b**) hybrid model: key cell metabolites [13].

Figure 13 and Figure 14 show prediction quality comparisons for both simulation approaches vs. experimental data, assessing the first-principles vs. hybrid (reaction + ANN) model. The superior fit performance of the hybrid vs. the conventional kinetic model is thus clear, even though the authors did not provide any metrics for comparative error quantification.

**Figure 15:**
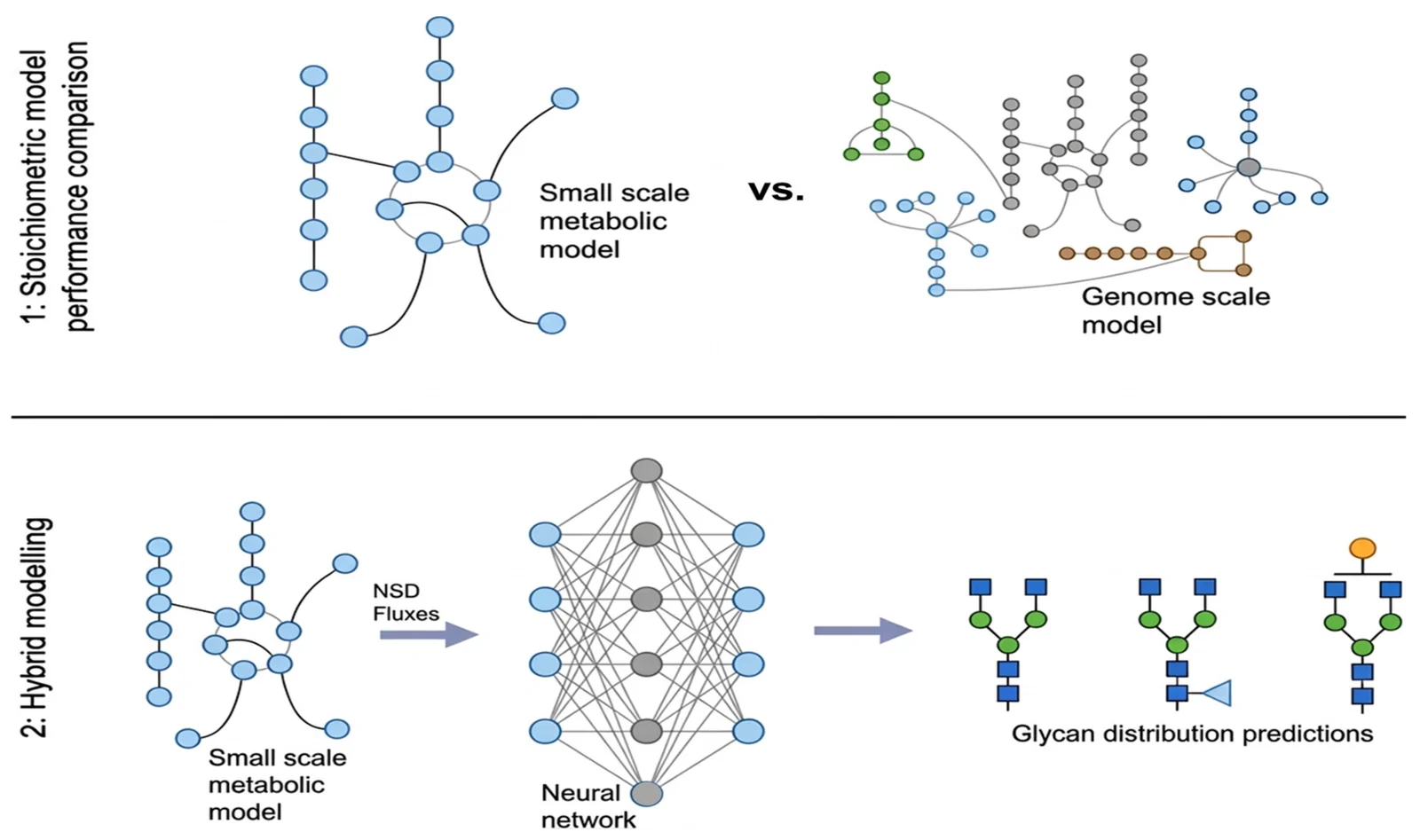
Hybrid modelling via metabolic kinetics, fluxes and NN for glycan prediction, from [15].

An advance to the study [8] was achieved by Antonakoudis et al. (2021) (Figure 15), who developed an ANN considering NSD fluxes computed by the stoichiometric model as inputs, and the glycan distribution of the protein of interest (here, IgG) as output [15]. Original NSD flux and glycan production data were used for ANN training, and another independent dataset was then employed so as to test ANN predictive accuracy (Table 3).

**Figure 16:**
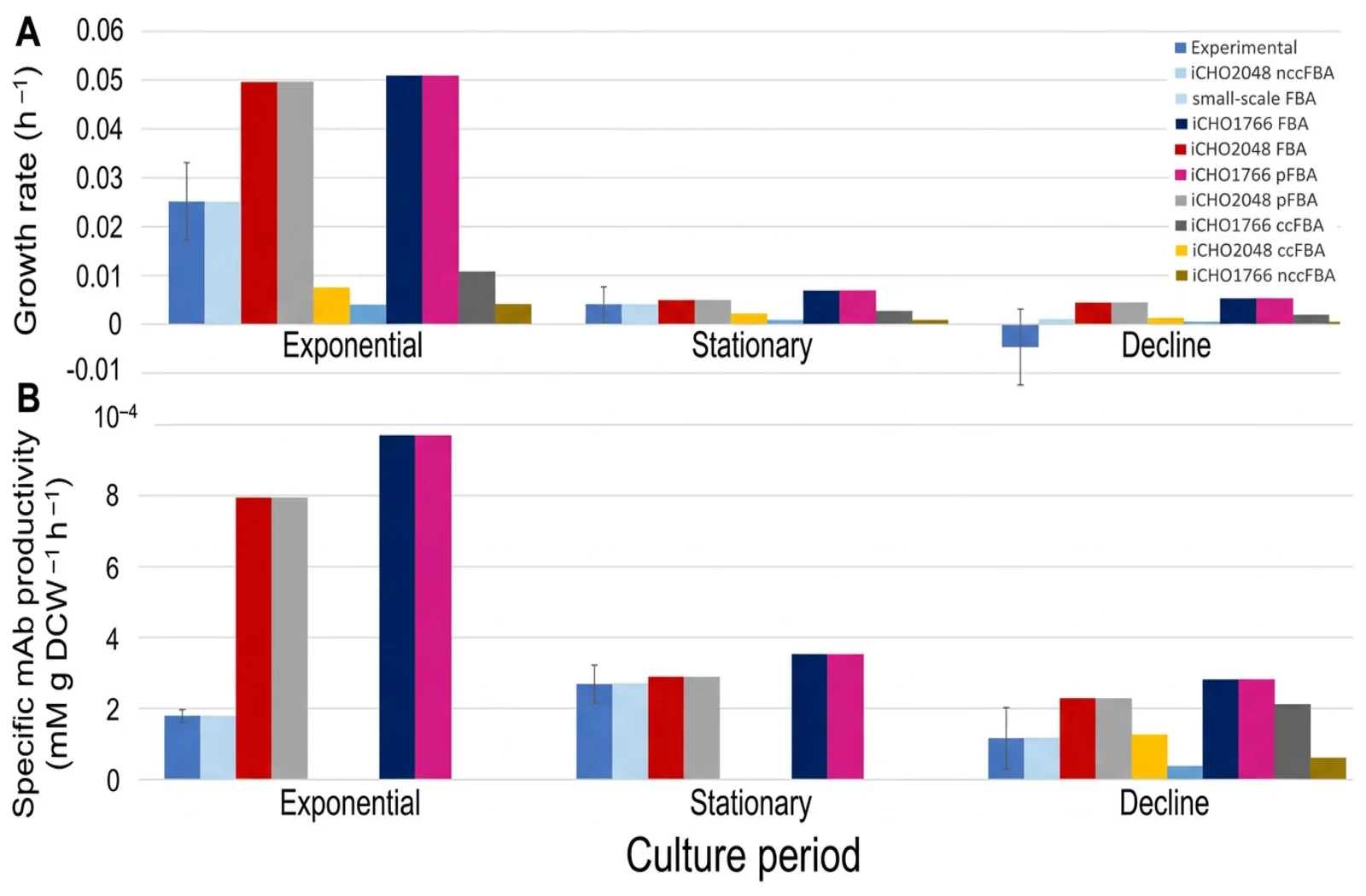
Biomass growth rate (**A**) and specific mAb productivity (**B**) FBA predictions, from [15].

A product-specific genome-scale model (GeM) constructed as per another study [16] implemented flux balance analysis (FBA), a linear programming method computing flux distributions that optimise a chosen objective function, subject to conservation constraints. Figure 16 illustrates the validation of FBS algorithms for biomass growth and mAb yield.

Figure 16A shows that the model was most accurate in predicting biomass growth rates in exponential rise and stationary phases, but not in the decline phase, where it failed to capture trends. Specific mAb productivity was predicted well in all periods (Figure 16B).

Botton et al. [17] proposed a hybrid digital (semi-parametric) model (HDM) which tracks viable culture cells, glucose, glutamine, lactate, ammonia, and mAb concentrations. The focus of their effort was *data augmentation*, where generating in silico results assists the expansion of datasets available from actual (in vivo) bioprocess experimental runs.

**Figure 17:**
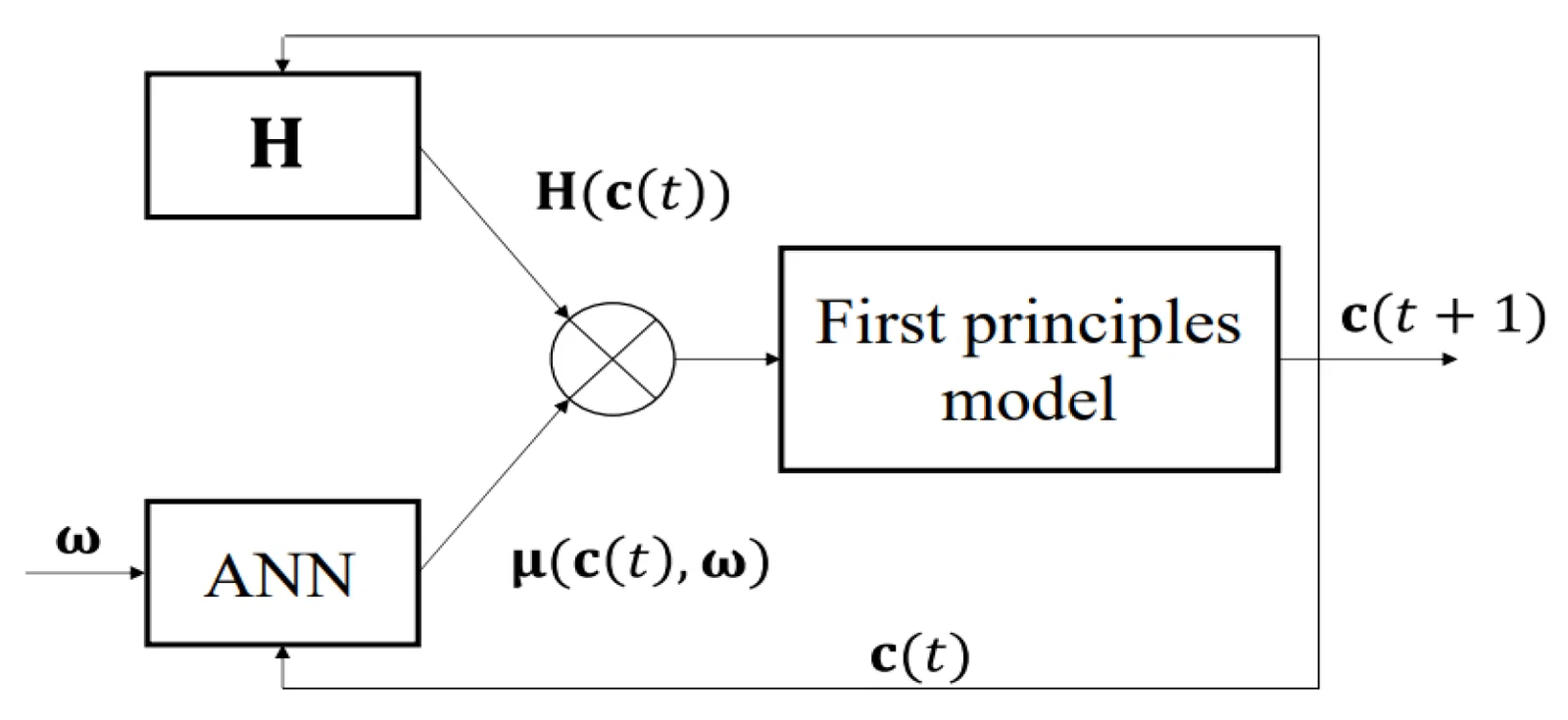
Hybrid digital model (HDM) block structure used to generate in silico batches [17].

The HDM structure (Figure 17) has a mechanistic section describing material balances of chemical species (*first-principles model*), and an ML section (*ANN*) employed to estimate the complex, unknown kinetic expressions from cell culture experimental data. Two data generation strategies were tested for final (mAb) product titer estimation: the first relied on a first-principles model only, the other on the said hybrid (HDM) model.

**Figure 18:**
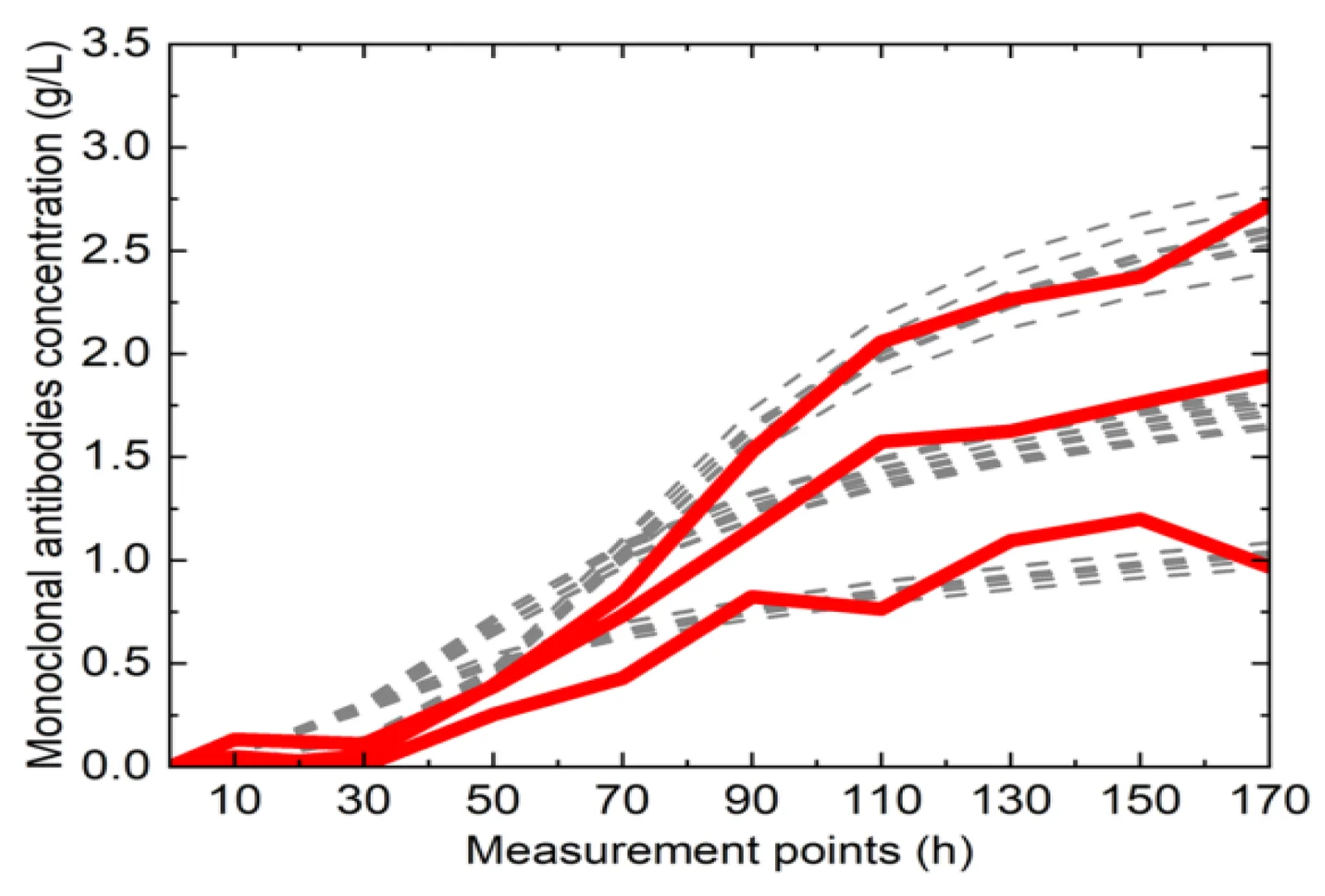
HDM training (solid red) and simulated (dashed grey) mAb titer profiles [17].

Figure 18 shows HDM-generated mAb titer profiles: Solid red lines are the 3 training batches from the process, while the dashed grey lines denote the 10 simulated trajectories. With 100 in silico batches generated, 10 time points from each were used for HDM training: all generated profiles were subsampled at the same 10 time points of real measurements. Resulting data are organised in matrix X_HDM_ [100×5×10] of culture variable trajectories, and vector y_HDM_ [100×1], with the harvest (final time) mAb concentrations. Very promising results were achieved: the HDM identified highly productive cell lines (high mAb titer), even when very few (2–3) actual experimental batches were available.

## 3. Process Analysis, Control and Optimisation

### 3.1. Process Control

ML methods hold promise for biopharma manufacturing process control [18,19,20]. NNs can analyse real-time bioreactor sensor data (temperature, pH, dissolved O_2_, nutrient concentrations) to monitor bioprocess progress. Learning from historical plant data, NNs can detect anomalies, deviations or drifts, and provide early warning for corrective action. By incorporation in hybrid (or standalone) process models and via real-time sensor data, NNs can reliably predict future process behaviour and optimise dynamic manipulations, ensuring optimal performance while accounting for dynamics and respecting constraints.

Integration into model predictive control (MPC) frameworks is an attractive idea; while bioprocess NN applications have abounded for decades, online process control studies are limited due to practical (real-time and hardware) implementation difficulties.

**Figure 19:**
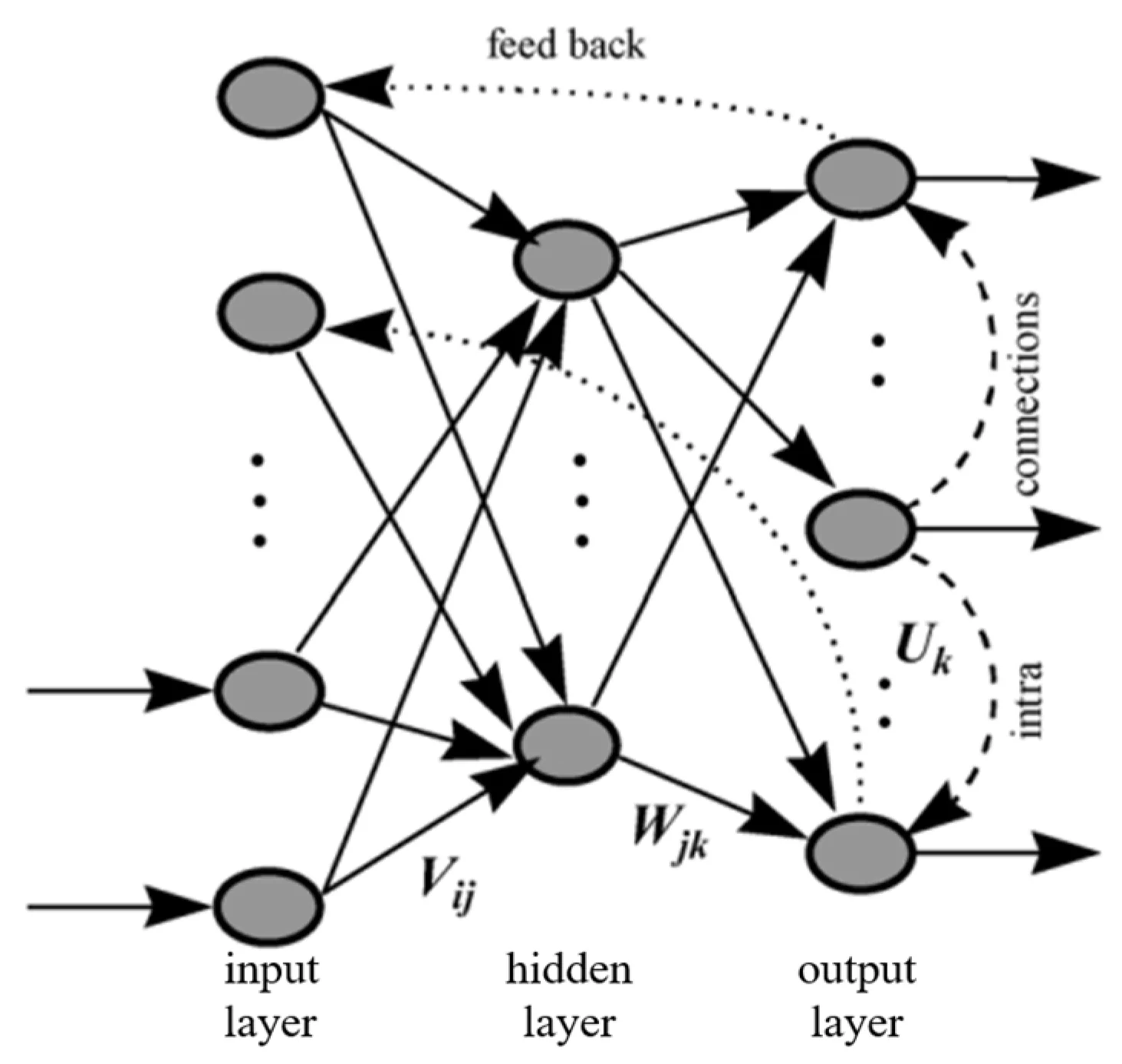
Schematic diagram of the proposed NN architecture with feedback action [20].

Gadkar et al. [20] used a recurrent neural network (RNN) study with output layer feedback and intra-connections, tracking a fed-batch yeast fermentation with adaptive weights computed from online concentration (dissolved O_2_) measurements (Figure 19). Substrate, ethanol and biomass concentrations are not measured but predicted by the NN.

Figure 19 shows the weights for connections between input and hidden layers (*V_ij_*), those for connections between hidden and output layer (*Wj_k_*), and the respective ones for weighted intra-connections (dashed lines) from the dissolved O_2_ (middle) to other output layer nodes (*U_k_*). Bias nodes (*ξ*_j_ and *ξ_k_*) usage is also essential for this NN formulation [20]. For NN tuning and architecture optimisation, a training dataset was generated at a constant 6 min sampling interval for 15 h, and random initial weights in the ±0.1 range.

**Figure 20:**
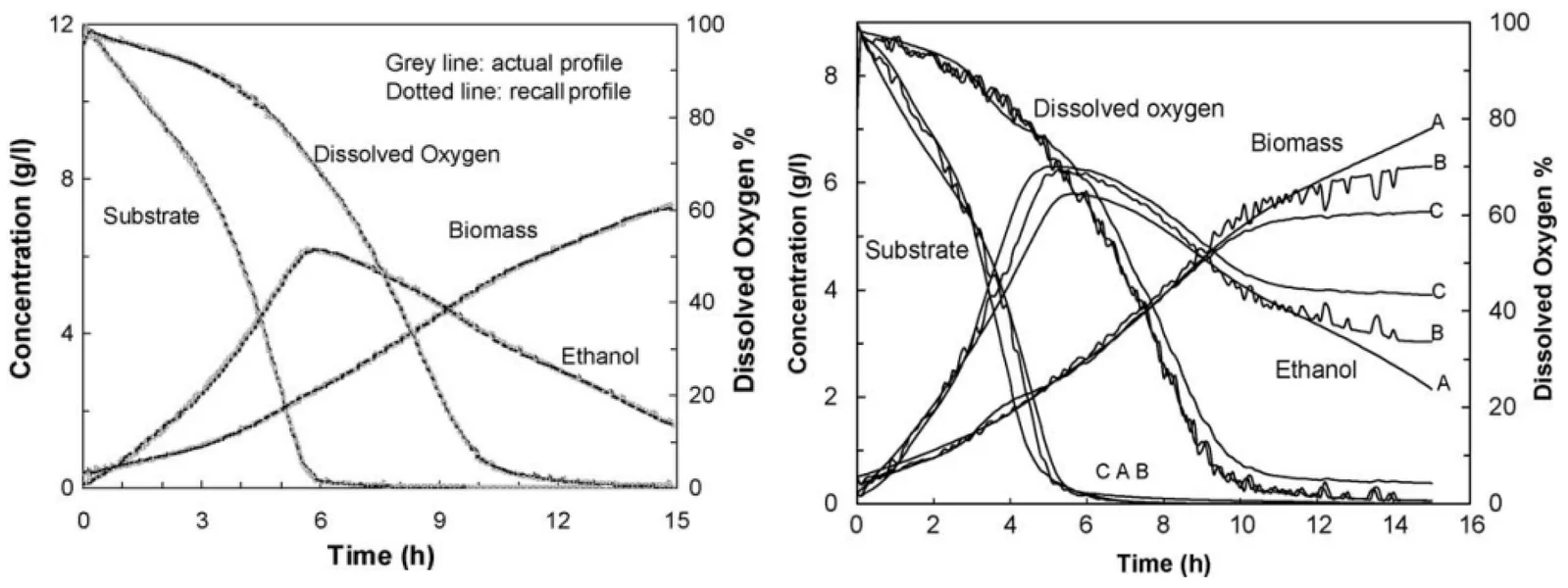
NN training (**left**) and profiles with/without online weight adaptation (**right**) [20].

Figure 20 (left) presents recall profiles from training, confirming excellent fit therein. To achieve reliable NN performance outside the training regime, however, all NN weights had to be continuously updated using the online adaptation algorithm described in [21].

Online weight adaptation as per [20] requires continuous information flow from actual experiments, via at least one online measurement of sufficient process importance. Dissolved O_2_ concentration meets this requirement, reflecting the process metabolic state. Given O_2_ availability and respiratory capacity, growth occurs on both glucose and ethanol.

Figure 20 (right) shows ΝΝ-driven process control (with/without weight adaptation) vs. noisy O_2_ experimental data, where NN predictions (B) with online weight updates are noisier but track more accurately concentration state profiles (A) than those without (C).

**Figure 21:**
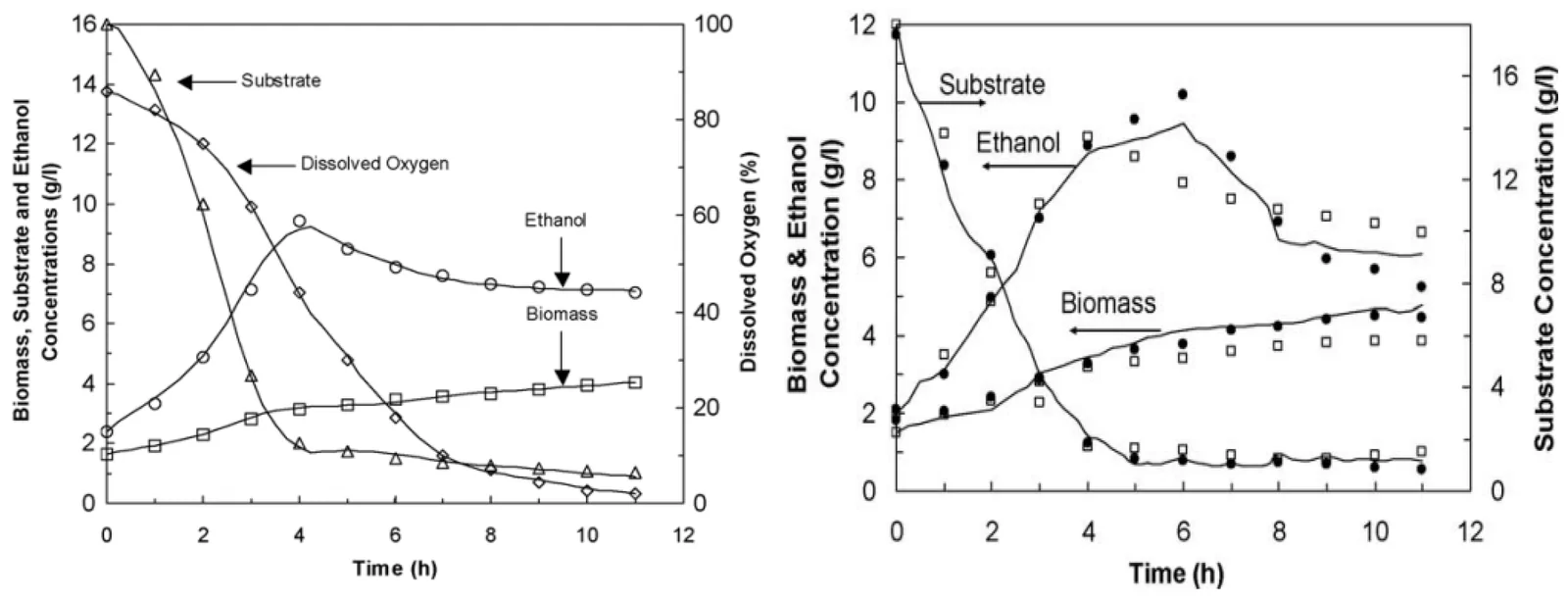
Training dataset (**left**); NN-driven online control signals vs. exptl. data (**right**) [20].

The NN is used to control cell concentration along a linear rise profile prescribed as:

(12) $$X=c*t+\;c_{1}$$

with *c* and *c*_1_ constants, *t* the elapsed time during fermentation and *X* cell concentration. Fermentation runs used a given medium (with 2% glucose, 2% peptone, 1% yeast extract) at Τ = 30 °C, pH = 5 and stirring (300 rpm), with *X* measured via optical density (600 nm). The NN was used to obtain state variable estimates for evaluating instantaneous feed rate. Figure 21 (right) shows NN-driven control improves a lot with online weight adaptation due to rapid computations (1–2 s), even if training occurs for variant *c* via Equation (12).

### 3.2. Process Optimisation

AI can revolutionise the optimisation of mAb manufacturing processes via improved efficiency, cost-effectiveness, and quality control. Big datasets from manufacturing plants can be analysed, to identify patterns or correlations overlooked by human perception.

Optimising process parameters (e.g., temperature, pH, agitation speed) can enhance mAb yield and purity (plant scale); planning inventory levels vs. demand variation, potential bottlenecks or broader disruptions (supply chain scale) yields financial benefits by reduced OpEx costs and improved efficiency of mAb production and distribution.

Traditional mAb manufacturing process optimisation involves testing a small set of specific (often expert-derived) hypotheses, via costly and time-consuming experiments. Data-driven methods are a promising alternative approach, relying on computer models with parameters inferred from process data, economising on experimentation expenses.

**Figure 22:**
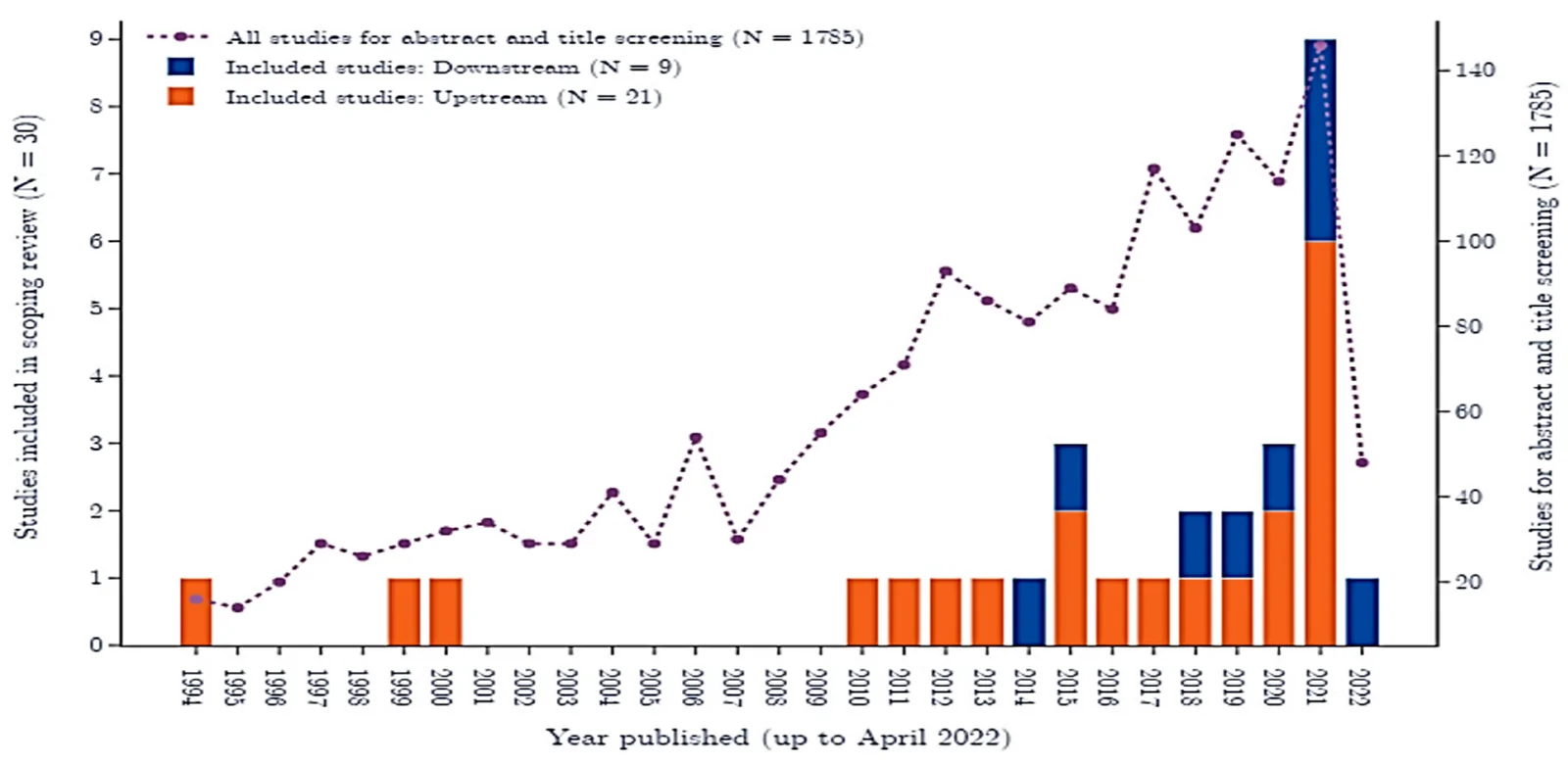
Bibliometric analysis of SLDO studies vs. biopharma publications, 1994–2022 [3].

Pham et al. (2023) explore Supervised Learning and Data-driven Optimisation (SLDO) studies in mAb batch process monitoring and control, illustrating bibliometric data on SLDO applications for mAbs [3]. Their keyword-based search strategy (‘SLDO methods’, ‘mAb bioprocesses’, ‘biomanufacturing’) across 3 decades (1994–2022) reveals that most papers concern upstream problems, and most (90%) were published between 2010–2022 (Figure 22).

**Figure 23:**
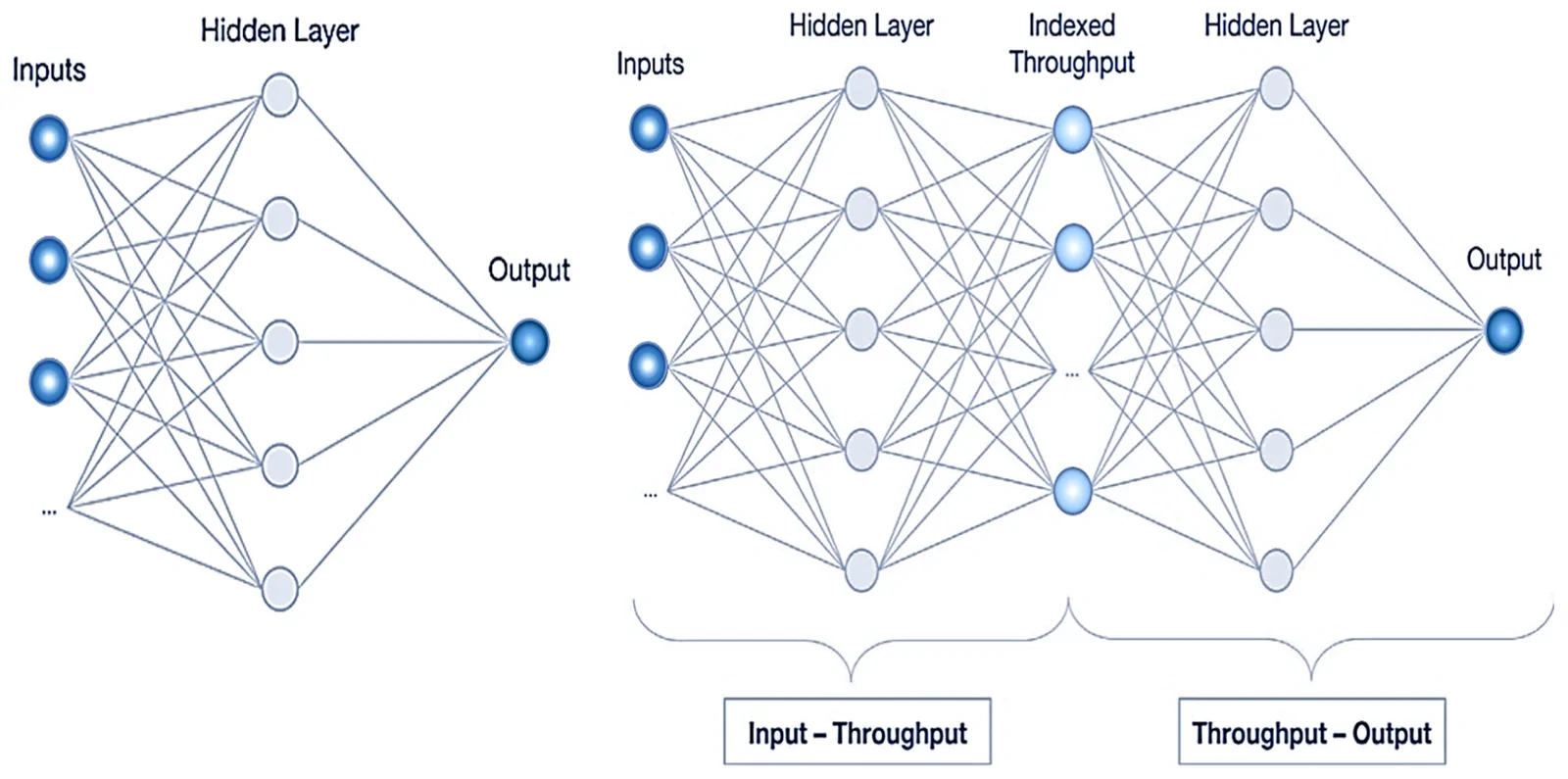
The Naive (**left**) and ITO (**right**) ANN model architectures, both adapted from [22].

Manapragada et al. [22] studied mAb manufacturing process optimisation in a fed-batch culture via ML (Figure 23), in case of insufficient data for surrogate model training, under two distinct (uniform and non-uniform) feeding patterns, as per its daily variation. The feeding pattern schedule is portrayed by a vector of 10 features, each representing the daily bioreactor feed amount for the entire duration (day 3 to day 13) of the culture period. Their input space *X* included the feeding schedule and concentration values for three (expert-selected) amino acids. The objective function to be maximised is product yield.

Figure 23 presents their ML model architectures employed: first, their simpler Naive model has just a 5-neuron hidden layer between the input and output layers. Their ITO model is a dual configuration for capturing the distinct lifecycle stages of cell cultures in mAb manufacturing: it comprises two shallow ANNs, the Input–Throughput and the Throughput–Output model, respectively. An intermediary logical layer (with indexed throughput nodes) forms the output of the first (but also input to the second) ANN [22].

Both in silico and in vivo experimental validation ensued: the first used 100 separate seeds to generate 100 objective function candidates for each (Naïve, ITO) type, and a grid search for 5·5·5 = 125 input combinations (the amino acids 2, 4, 17, varied at 5 levels each) produced the best decision vector, maximising final mAb product concentration (Table 4).

**Figure 24:**
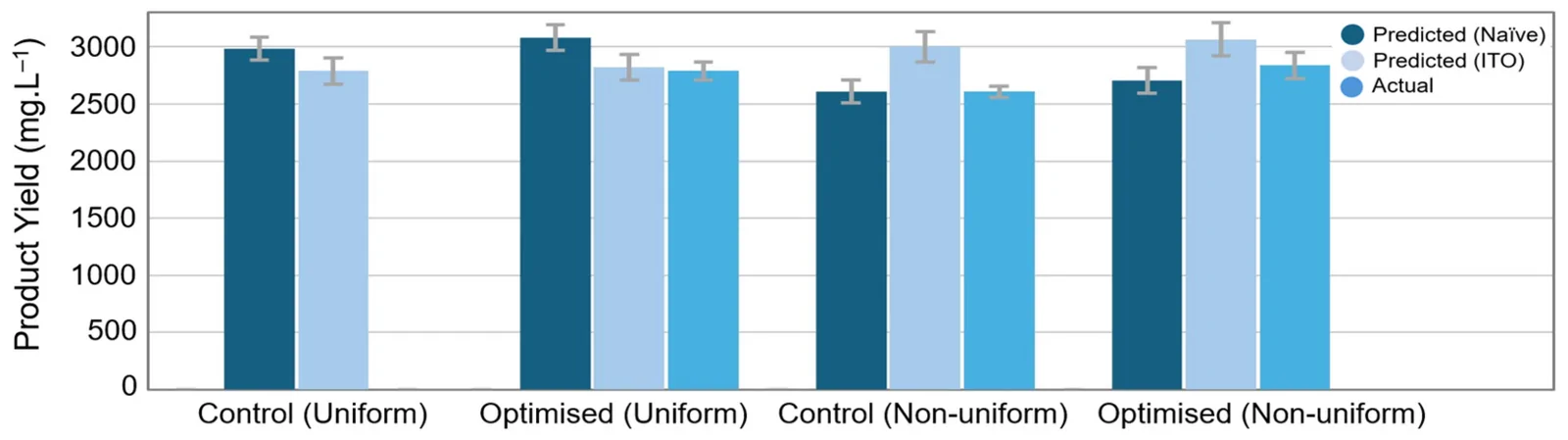
Predicted vs. actual mAb yield for uniform and non-uniform feeding cases, data by [22].

For the in vivo experiments, a six-bioreactor fed-batch study tested two optimised feed flow regimes (non-uniform as well as uniform feeding) vs. the respective reference (control) base cases. Table 4 summarises results for all cases, and Figure 24 compares all in silico runs vs. experiments: both ITO and Naive models predict the outputs accurately.

**Figure 25:**
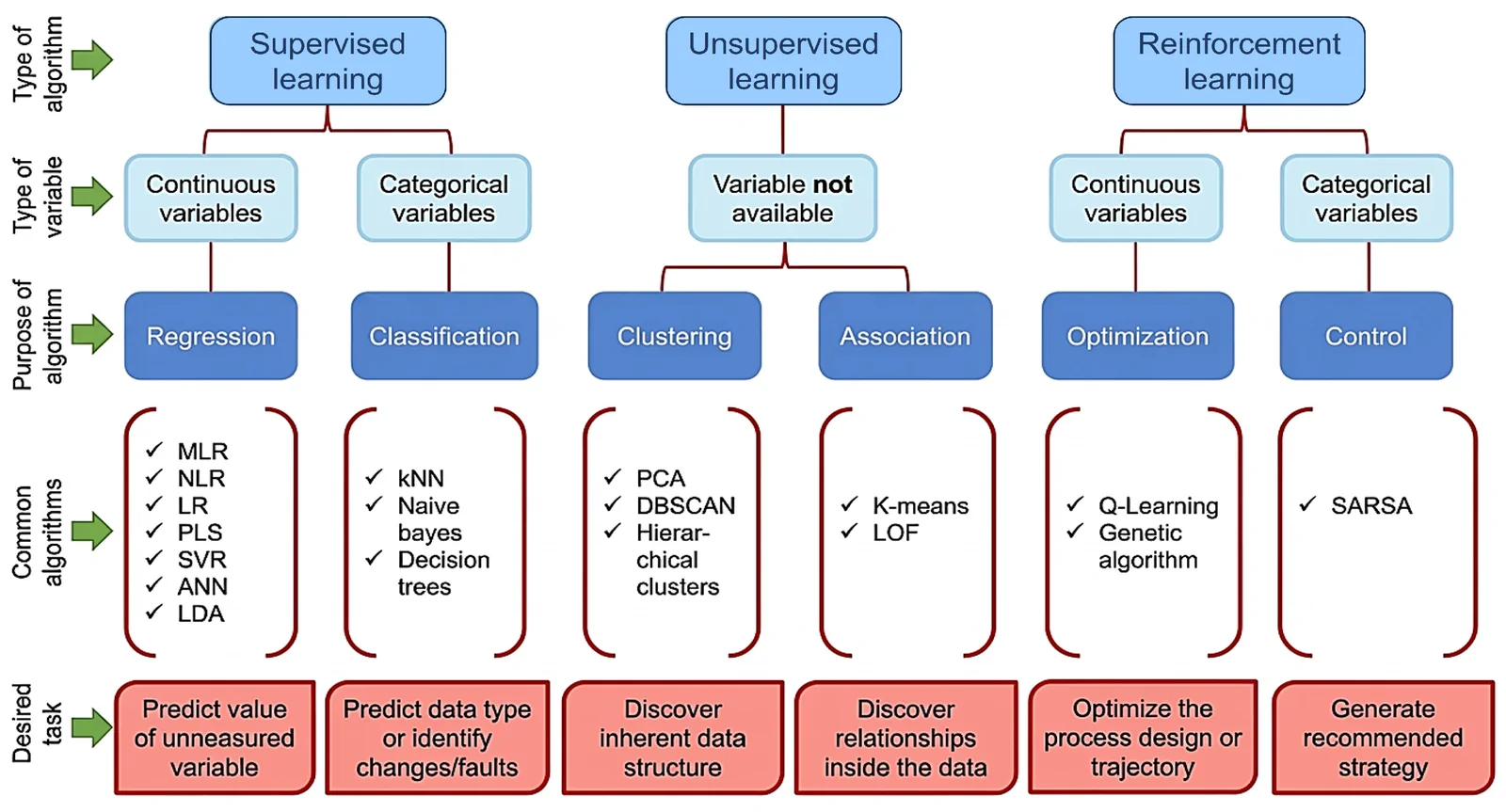
A comprehensive taxonomy of AI/ML methods vs. learning types, adapted from [10].

A review by Rathore et al. [10] summarises AI/ML applications in biopharmaceutical manufacturing, with focus on multivariate data analysis, ANN and RL methods (Figure 25). Optimisation and control of biopharma unit operations is challenging, as it often involves complex phenomena (e.g., protein expression in cell culture bioreactors, binding on composite chromatography resins, pH-driven virus inactivation, ultrafiltration via semi-permeable membranes). The authors opine that there is an exceptionally promising scope for AI/ML at plant-wide manufacturing scale, due to vast datasets from diverse process sensors (e.g., pH, temperature, conductivity, redox, UV, weight, level, flow rate, pressure). Many subsequent studies confirmed this booming field of sustained R&D interest [23,24,25,26]. A focal challenge is accelerating bioprocess scale-up via ML-driven frameworks [27,28].

The Dewasme et al. model was used for optimisation, to compute optimal medium renewal time and composition, to maximise mAb production at lower substrate cost [5]. The goals form an objective function, subject to the validated model of Equations (1)–(11).

(13) $$J_{obj}=mAb{{(t}_{renewal})}^{2}+mAb{{(t}_{f})}^{2}-\alpha (G_{renewal}^{2}+{Gn}_{renewal}^{2})$$

where α is a weight coefficient accounting for substrate savings vs. mAb production, thereby arbitrarily specifying the predominance of one optimisation target over the other. The MATLAB^®^ 2025a *fmincon* (constrained optimisation) algorithm is used to find the optimal decision vector *θ* = [*t_renewal_ G_renewal_ Gn_renewal_*]^T^, i.e., the medium renewal time, and the glucose and glutamine concentrations in culture medium at that medium renewal point.

**Figure 26:**
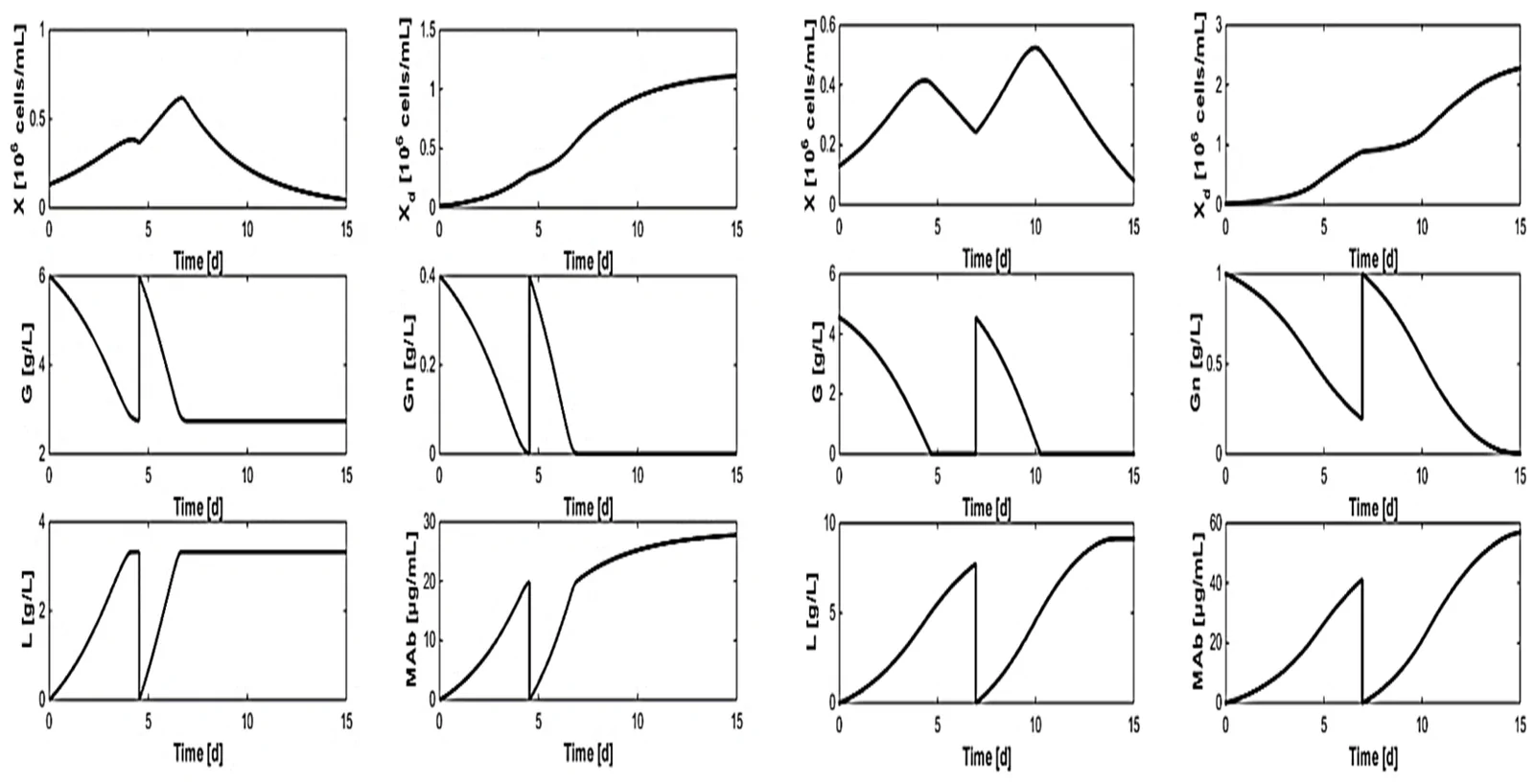
mAb production maximisation vs. weight variation: α = 0 (**left**), α = 10 (**right**), as per [5].

Figure 26 depicts optimisation results for α = 0 (disregarding substrate cost savings) and α = 10 (ascribing a strong emphasis on substrate cost savings vs. mAb production). The optimal medium renewal time is *t_renewal_* = 4.54 vs. 7 days (i.e., much later), respectively. The final mAb concentration is 60.92 vs. 75 µg.mL^−1^, respectively (after the 14-day period); thus, even the first (smaller) improvement is a 30% gain vs. experiments (40–45 µg.mL^−1^). A more economical fermentation may thus last 56% longer (before first renewal), but also offer a remarkable (23%) extra mAb production increase, in addition to the said for α = 0.

MLPCA thus reliably extracts critical information from experimental data, achieving:

- Rapid stoichiometry estimation that is independent of reaction kinetics, reducing the number of unknown model parameters (half of the latter are actually stoichiometric).
- A small number of reactions, to avoid useless model complication vs. experimental rig while capturing underlying biological mechanisms (viability, growth, inhibition).

**Figure 27:**
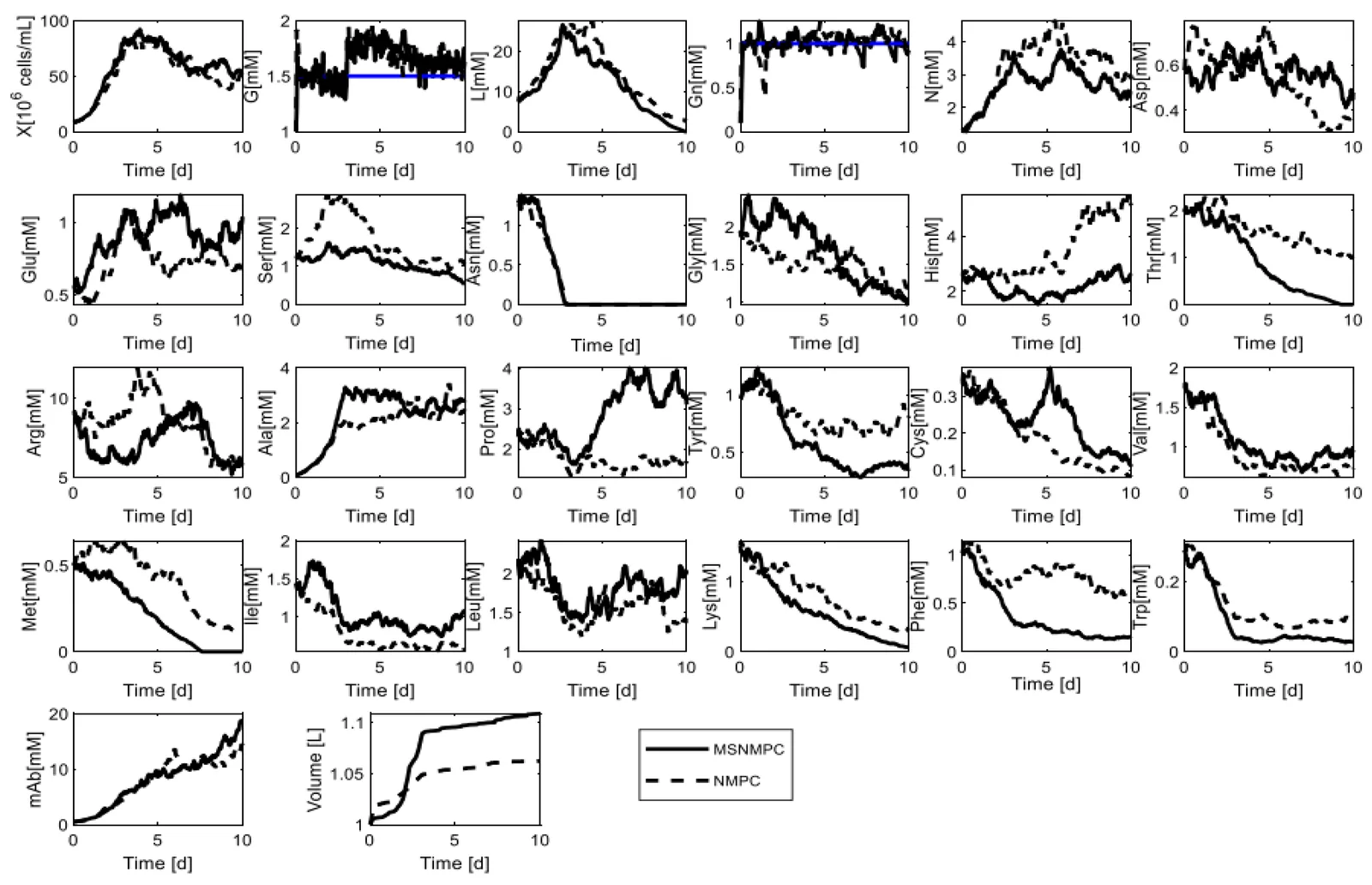
State trajectory comparisons: MSNMPC vs. NMPC optimisation algorithms, as per [6].

Their subsequent study via the same model [6] presents a comparative evaluation of multi-stage vs. classical nonlinear model predictive control (MSNMPC vs. NMPC), with the “divide and conquer” approach allowing for stoichiometry and reaction kinetics to be estimated iteratively (separately or simultaneously), via estimates from previous steps. All 25 state variable concentration (and volume) trajectories are illustrated in Figure 27.

**Figure 28:**
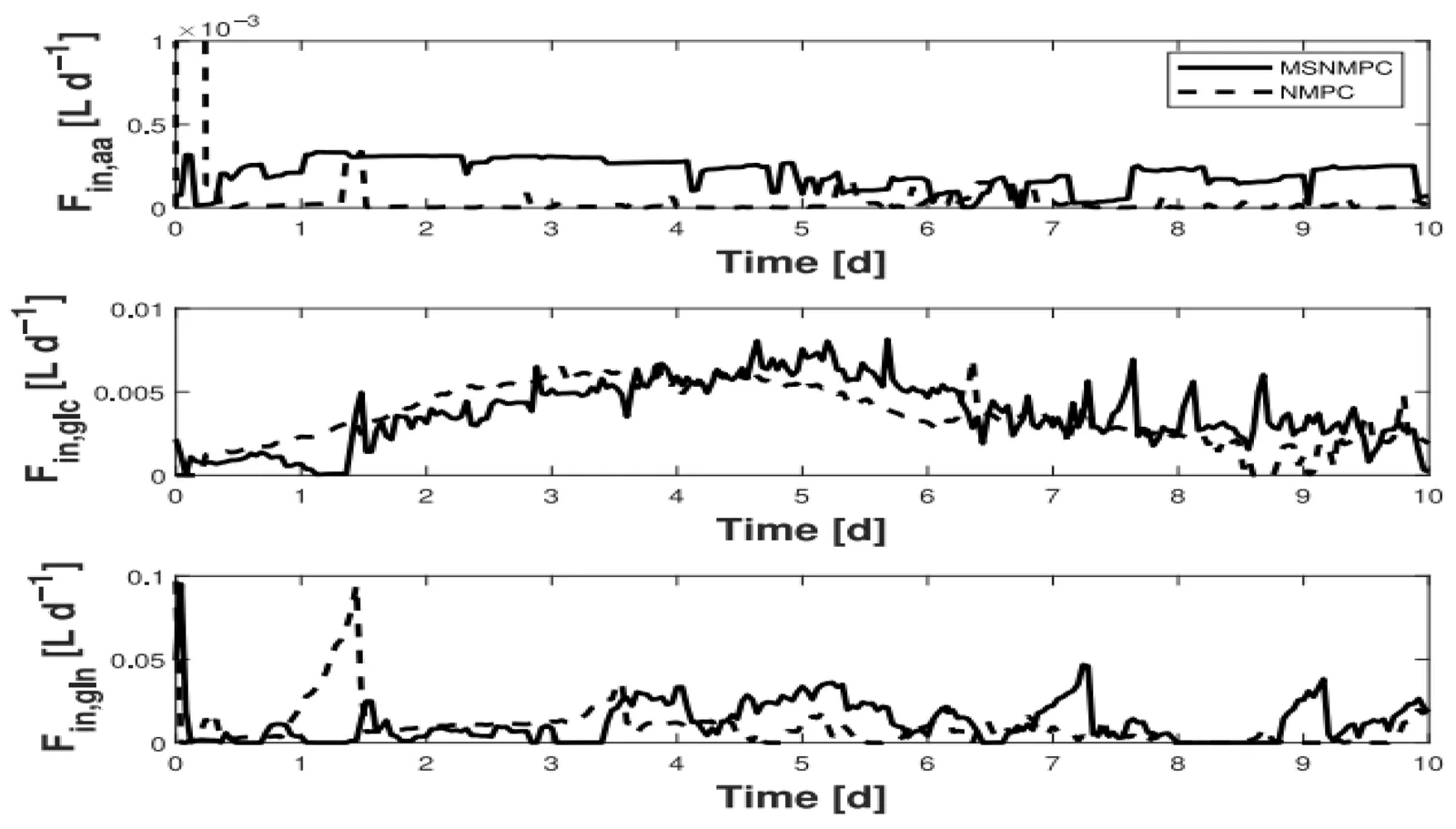
Feed rate trajectory comparison: MSNMPC vs. NMPC optimisation algorithms [6].

Figure 28 presents MSNMPC vs. NMPC optimised feed rate profile comparisons: the first achieves better performance via faster but also smaller actuator variations, while the second features smoother input trajectories with abrupt peak variations in the first 2 days. Glutamine (bottom panel) shows early abrupt rises in both cases, then rapidly attenuates.

The authors conclude that NMPC computes continuous reaction rates but cannot also detect possible metabolic switches cancelling/activating some of the rates at critical times. The MSNMPC model is challenged and compared to the classical economic NMPC, using a simulated plant with discontinuous reaction kinetics representing metabolic switches. Results show that classical NMPC performs well, but the MSNMPC algorithm achieves superior accurate tracking and increased robustness vs. structural uncertainties globally.

Le et al. (2018) explored the development of a rapid ML-based analytical method for mAb manufacturing quality control, focusing on the production of four mAbs (Infliximab, Bevacizumab, Rituximab, Ramucirumab) in a range of conditions and concentrations [29]. This can be critical, e.g., for ensuring mAb quality before administration by medical units. The study used a novel collaborative data challenge platform, namely Rapid Analytics and Model Prototyping (RAMP), collating problem solutions from ca. 300 data scientists.

**Figure 29:**
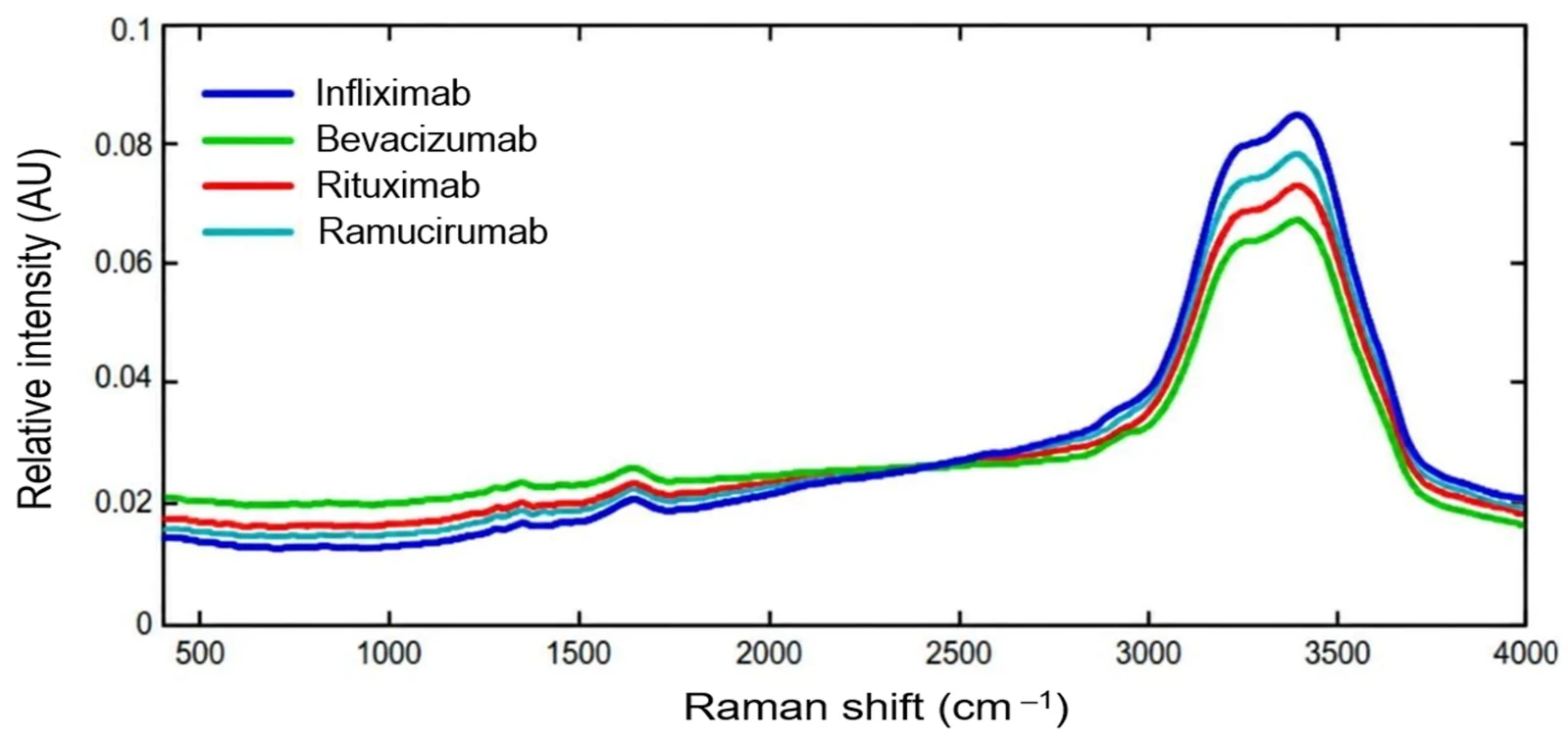
Mean raw Raman spectra for the four mAbs studied (400–4000 cm^−1^); adapted from [29].

ML analyses occurred in partnership with the Paris-Saclay Centre for Data Science (CDS) using the Scikit-Learn library [30]; Figure 29 shows Raman spectra for all mAbs. Raman and near-infrared (NIR) spectroscopy enable direct mAb measurements through glass/plastic packaging (unlike HPLC/UV or LC/MS/MS chromatography), so a linear and an ML method were comparatively used towards classification and regression (Table 5). Classification via Principal Components vs. Partial Least Squares versions of Discriminant Analysis (PCA-DA and PLS-DA), and via ML, was used to estimate mAb concentrations.

The challenge here is to predict mAb concentrations in solution: Table 5 results clearly indicate that the ML approach achieves much lower error metrics for all four mAbs, and overall. With linear (PCA-DA and PLS-DA) tools, 63% of samples at a low concentration ≤ 1 mg/mL) had error of over 15%; the error is much smaller with the use of ML (5.6%). ML model bias is negligible at concentrations above 0.1 mg.mL^−1^; relative error drops sharply as mAb concentration of 15% (*y* < 1 mg.mL^−1^) falls to 5% (*y* > 3 mg.mL^−1^).

The ML models employed are sensitive to the scale of data: the majority of effective prediction strategies use nonlinear kernel-based methods, e.g., kernel PCA for nonlinear dimensionality reduction [31] and support vector machines (SVM) for prediction [32]. Nitika et al. (2024) used convolutional neural networks (CNN) with Raman spectroscopic data in their process analytics tool for mAb charge variant monitoring and prediction [33].

Another fruitful field for mAbs optimisation applications is production operations. Response surface methods (RSM) offer multi-dimensional visualisation of cause (x, y axes) vs. effect (z axis) [34,35], and are a lot more intuitive than PCA/PLS approaches [36,37]. Bashokouh et al. (2019) [36] used an ANN to optimise cell culture cultivation conditions (i.e., incubation time, temperature and foetal bovine serum/FBS concentration) to maximise IgM mAb production by hybridoma M1A2 cells. Two cell culture media were used: Dulbecco’s Modified Eagle Medium (DMEM) and Roswell Park Memorial Institute Medium (RPMI 1640). Experimental data were analysed via the three-layer feed-forward ANN structure to establish a predictive model that could optimise IgM mAb production.

The ANN comprises a three-neuron input layer (cultivation time, temperature, FBS), an eight-neuron hidden layer, and a single-neuron output layer (IgM mAb concentration), trained with back-propagation to find the optimal topology via RMSE and R^2^ metrics [36]. IgM concentration measurements were obtained via a protocol by Ishida et al. (2018) [38].

Table 6 shows the highest IgM mAb production (1220 µg.mL^−1^) is obtained in DMEM with 17% FBS, cultivated for 5 days at 33 °C; the mAb production drops to 719.24 µg.mL^−1^ for a DMEM at low (7%) FBS, at identical cultivation time and temperature (thus a 41% reduction in IgM mAb production occurs for a 59% FBS concentration reduction).

**Figure 30:**
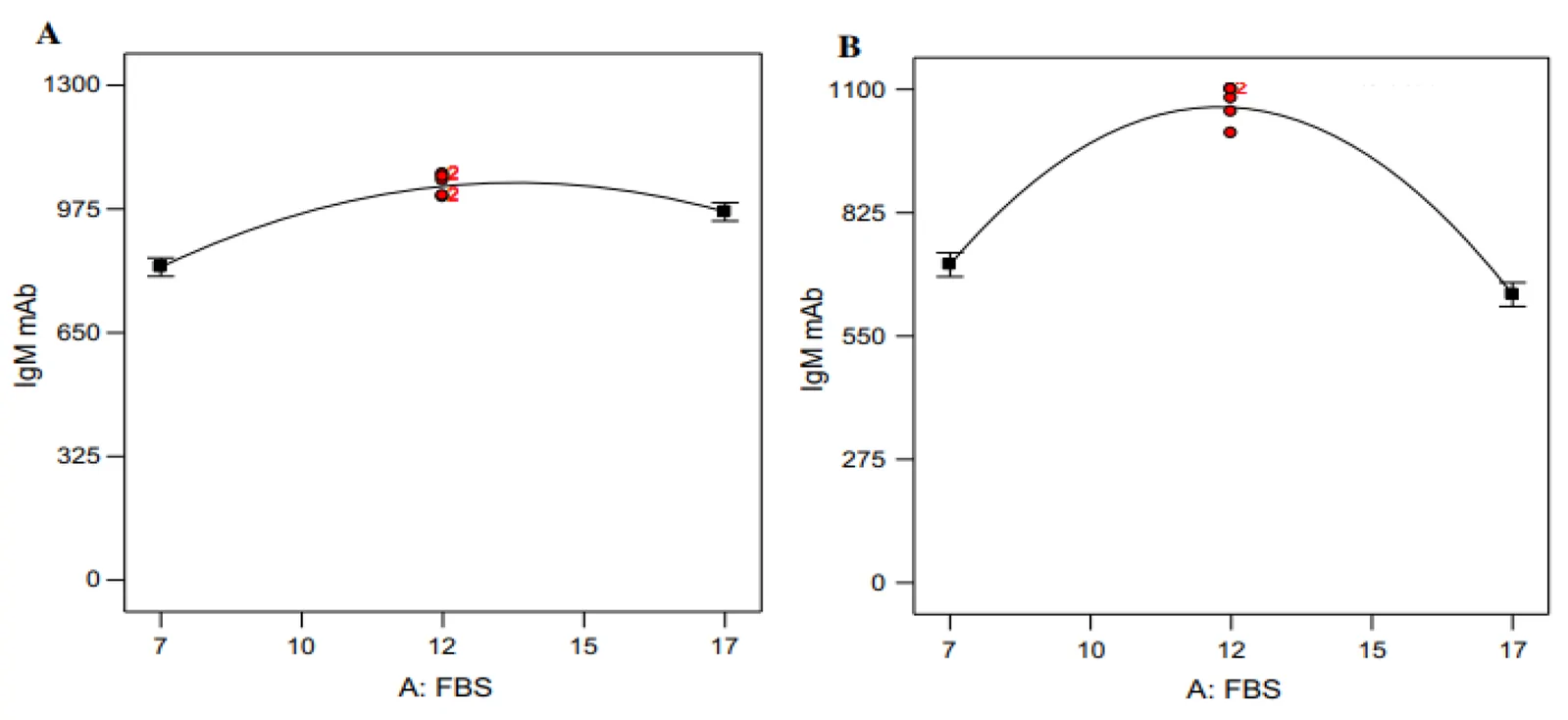
Effect of FBS concentration on mAb production in DMEM (**A**) vs. RPMI 1640 (**B**) [36].

**Figure 31:**
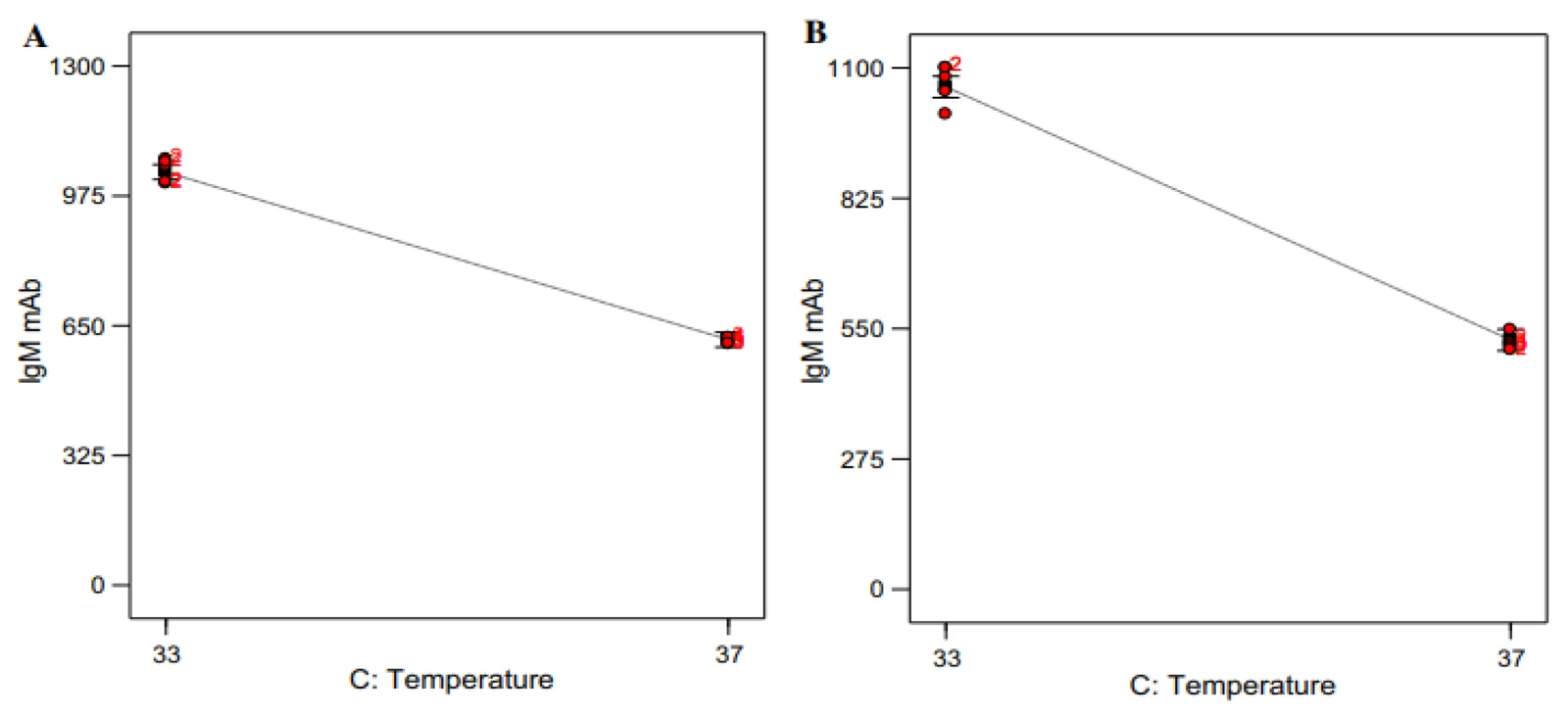
Effect of temperature on mAb production in DMEM (**A**) and RPMI 1640 (**B**) [36].

**Figure 32:**
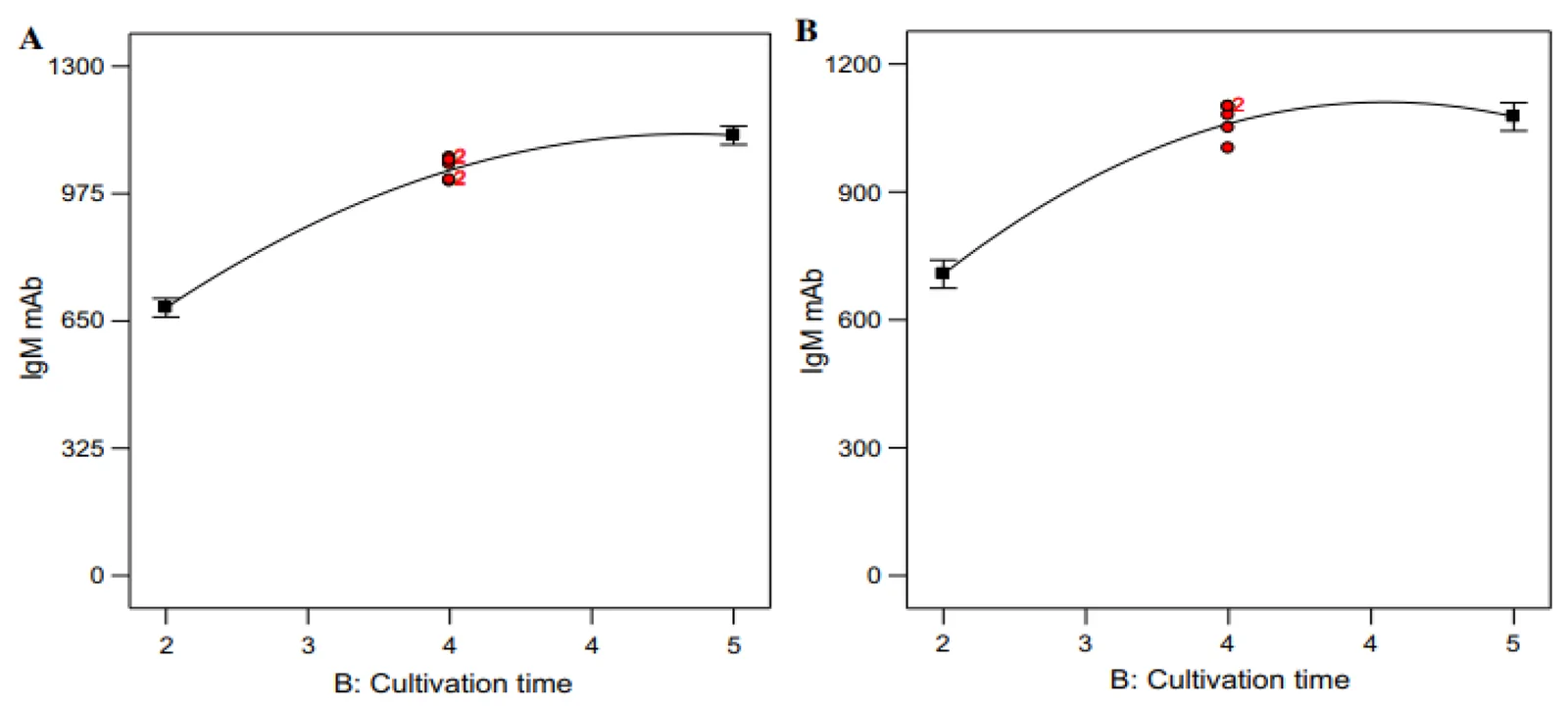
Effect of cultivation time on mAb production in DMEM (**A**) vs. RPMI 1640 (**B**) [36].

Figure 30, Figure 31 and Figure 32 present IgM concentration curves vs. each of the three operational variables, indicating similar trends (but with substantial differences in mAb productivity).

The mAb results vs. FBS concentration results agree with Mel et al. (2008) [34], who studied FBS effects (5–15% range) on RC1 hybridoma viability (IgG mAb-secreting cells). Temperature can impede mAb growth (Figure 31), as per Chong et al. (2008) [39], who show hybridoma C_2_E_7_ cell growth is slower under mild hypothermic (32 °C) conditions; maximum viable cell density is 10% lower, but mAb productivity is 5% higher vs. 37 °C.

**Figure 33:**
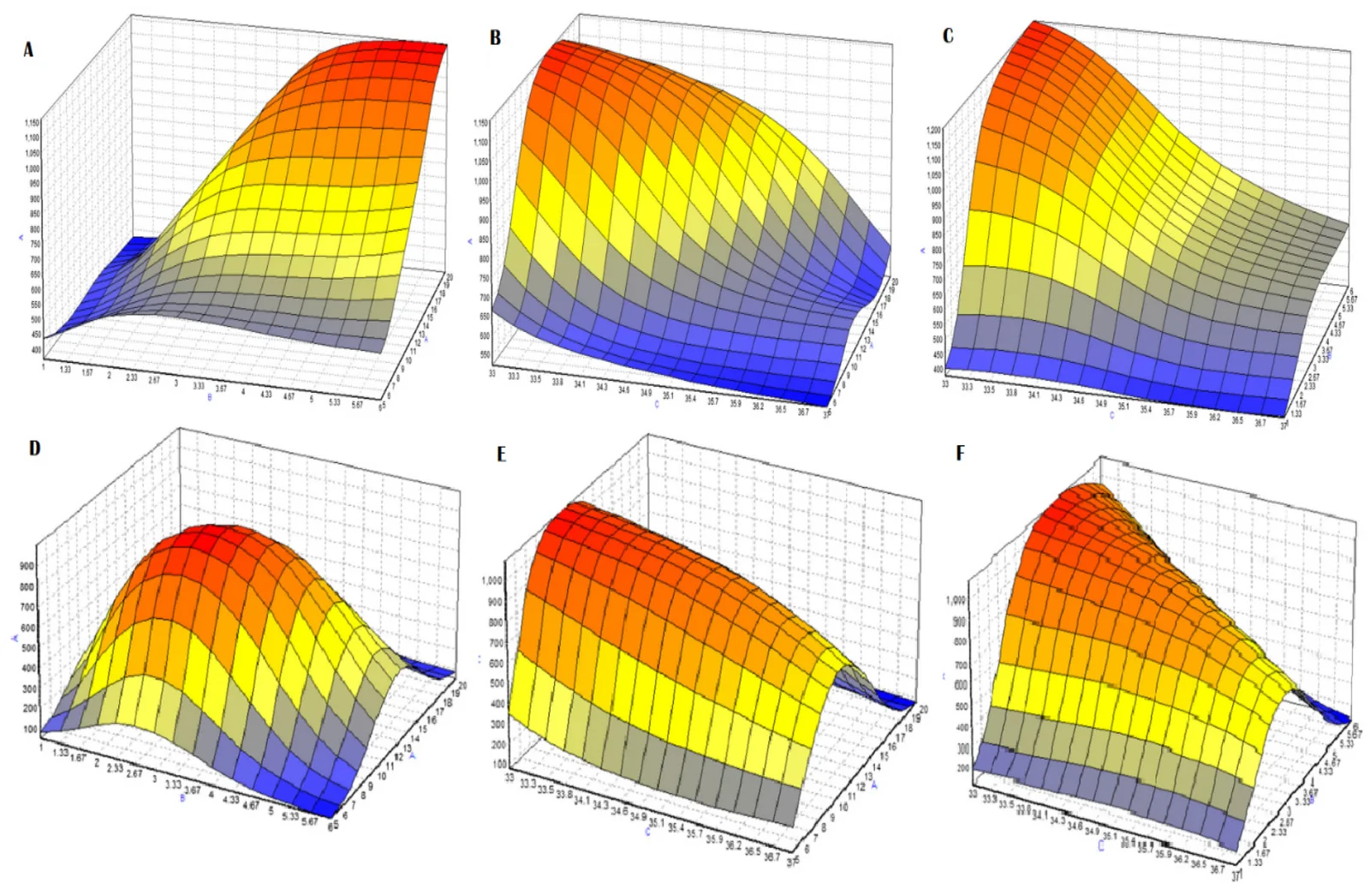
Bivariate mAb production: (**A**,**D**) FBS concentration + cultivation time; (**B**,**E**) FBS concentration + T (°C); (**C**,**F**) cultivation time + T (°C) (media: DMEM/top; RPMI 1640/bottom) [36].

The 3D plot (Figure 33) transcends the univariate RSM view of Figure 30, Figure 31 and Figure 32, as it shows mAb productivity (z-axis) vs. two-at-a-time of three independent variables; beyond capturing evident local maxima, the culture media also clearly affect yield (A–C vs. D–F).

Maximised mAb productivity may suffice at bench- but not production-scale [40,41,42], since technical *as well as* economic performance metrics are pivotal to biopharma ventures. George and Farid (2007–2009) in stochastic combinatorial multiobjective optimisation (MOO) studies employed [41,42] went beyond previously established mathematical approaches (e.g., mixed-integer (non)linear programming, MILP/MINLP) of optimisation frameworks for manufacturing capacity planning and scheduling in mAb plants [43,44].

**Figure 34:**
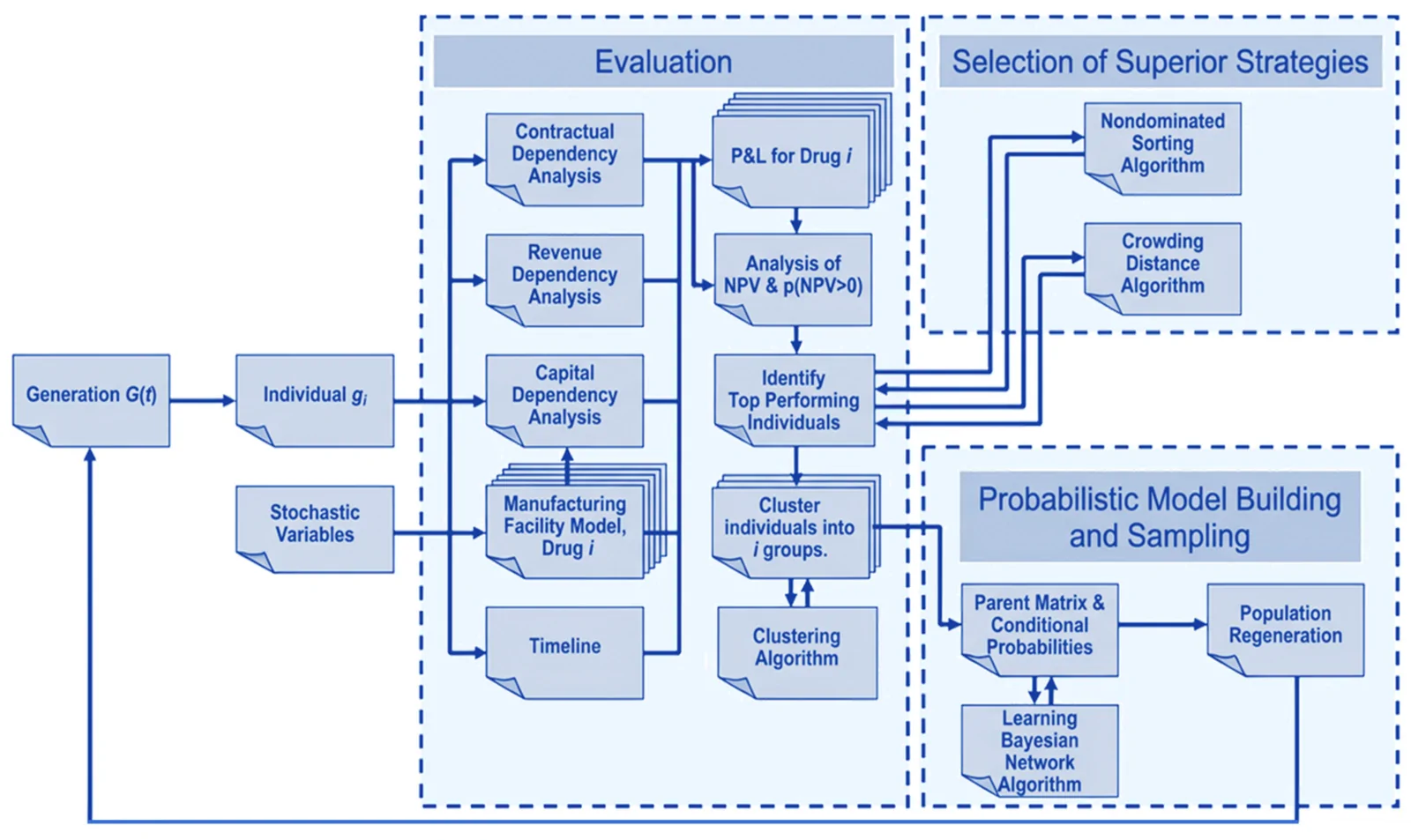
The complete biopharma simulation and optimisation framework, adapted from [41].

A combined ML-evolutionary computation strategy was chosen to tackle the significant complexity of the problem via of an estimation-of-distribution algorithm (EDA). The complete stochastic optimisation framework employed is illustrated in Figure 34 [41].

Model development combined C++ (for fast calculations saving CPU time) and MS Excel/VBasic (versatile in data storing, extraction, manipulation and plotting). The reward (profitability) and risk metrics for identifying the best strategies were the mean positive net present value (NPV) and the probability for it to be positive *p* (NPV > 0), respectively.

Economic performance vs. multiple objectives in an uncertain environment requires case studies; for instance, that in [42] assumes a biopharma company with 10 mAb drug candidates available for development, which must decide on a drug portfolio of limited size. The optimisation model considers commercial product characteristics, technical success probabilities, durations, revenue and costs (capital and operating expenditure, royalties). Stochastic variables addressed are taken to obey triangular probability distributions, (convenient for small datasets). Four cash flow constraints (i.e., −$75 MM, −$200 MM, −$200 MM, unconstrained) portray company cash limitations to R&D funding.

The stochastic optimisation settings include: max. number of iterations (*t_max_*) = 17, candidates in each generation/iteration (|*G*(*t*)*|)* = 1000, Monte Carlo trials per candidate strategy (*U*) = 250, and superior candidate strategies in *G*(*t*) at each iteration (|*S*(*t*)|) = 500.

**Figure 35:**
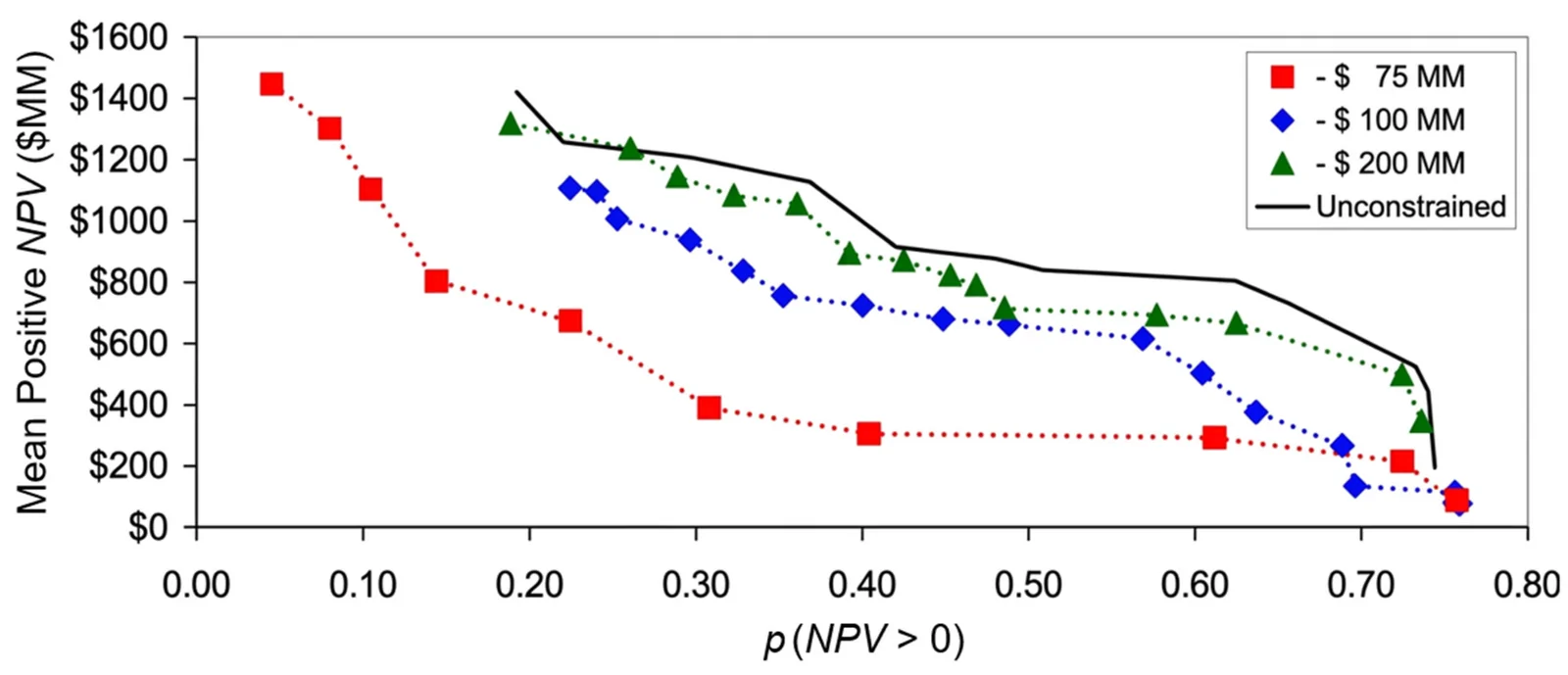
Mean NPV vs. risk for a 5-drug portfolio and 4 cash flow constraints, adapted from [41].

Figure 35 shows optimisation results for a five-drug portfolio under said constraints, with four clear Pareto fronts and strong relation (trade-off) between NPV and *p*(NPV > 0). For a given risk level *p*(NPV > 0), each tighter cash flow constraint reduces the mean NPV.

The rigorous formulation of [42] combines ML and evolutionary computation under uncertainty, achieving efficient decision space search and reliable tracing of a Pareto front.

Table 7 summarises key features of impactful publications discussed in this section.

### 3.3. Process Monitoring and Control

The biopharmaceutical industry follows good manufacturing practices (GMPs), but process and product variability (often due to feedstock variability) pose great challenges. Multivariate statistical methods, as per [5,6], have been established for decades in batch process monitoring (BPM), and can efficiently monitor and control biopharma processes.

Nikita et al. (2022) and Park et al. (2023) published two papers summarising many advances on real-time quality prediction in continuous mAb manufacturing [45] and on digital twins for multistep forecasting of cell culture evolution profiles, respectively [46]. Biochemical complexity necessitates the development of elaborate frameworks in the case of continuous mAb manufacturing, e.g., a study on control of surge tanks employs four layers (data acquisition, process scheduling, deviation handling, real-time execution) [47].

**Figure 36:**
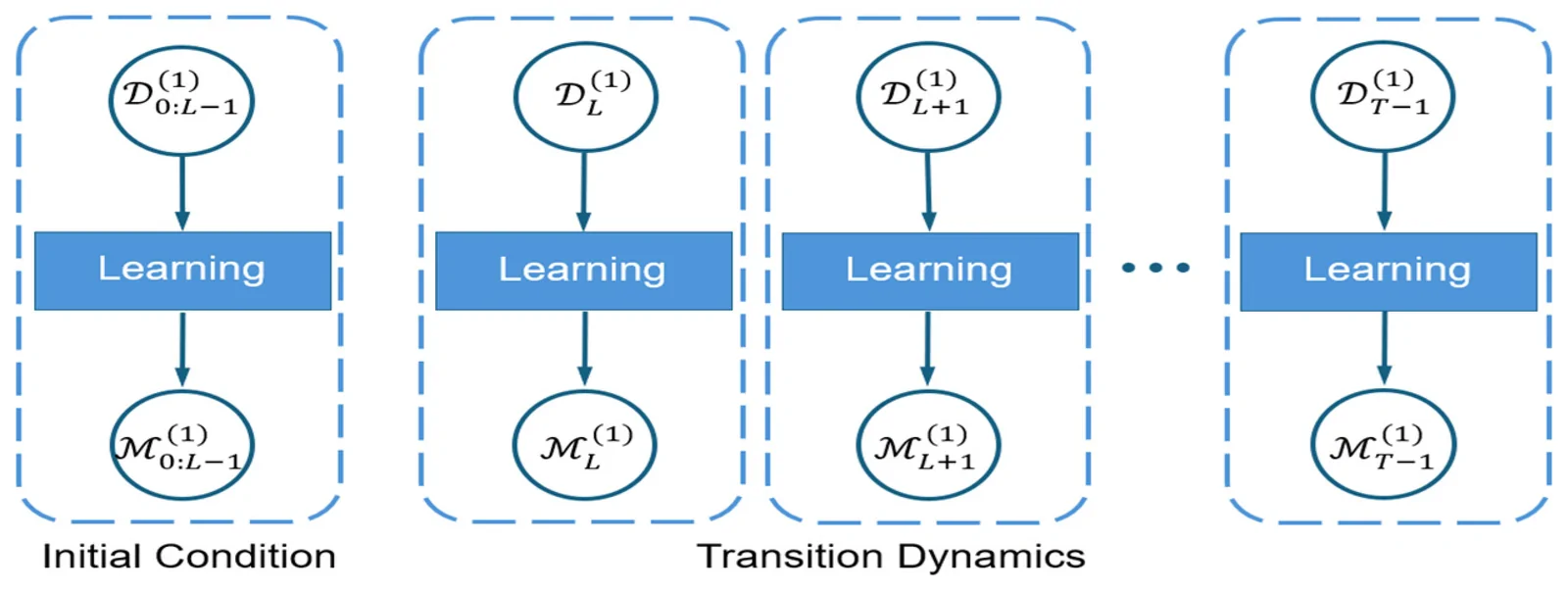
Sparse block-wise learning, adapted from the GP-SSM generator model shown in [48].

Tulsyan et al. (2019) [48] addressed the real-time statistical process control (SPC) problem for processes with limited production history or modest data availability (*Low-N* datasets). Low-N scenarios pose several BPM challenges, so a transition from a Low- to a Large-N scenario can involve generating an arbitrarily large number of in silico batch datasets [48]. This happens in two steps: first, stratified resampling occurs on the original data stored in the high-frequency data acquisition system (DAQ); then, a generator model is trained on the said resampling data, via a Gaussian process state-space model (GP-SSM) (belonging to a class of Bayesian non-parametric methods) which provides the flexibility for portraying complex, nonlinear, non-stationary (batch) process dynamics (Figure 36).

**Figure 37:**
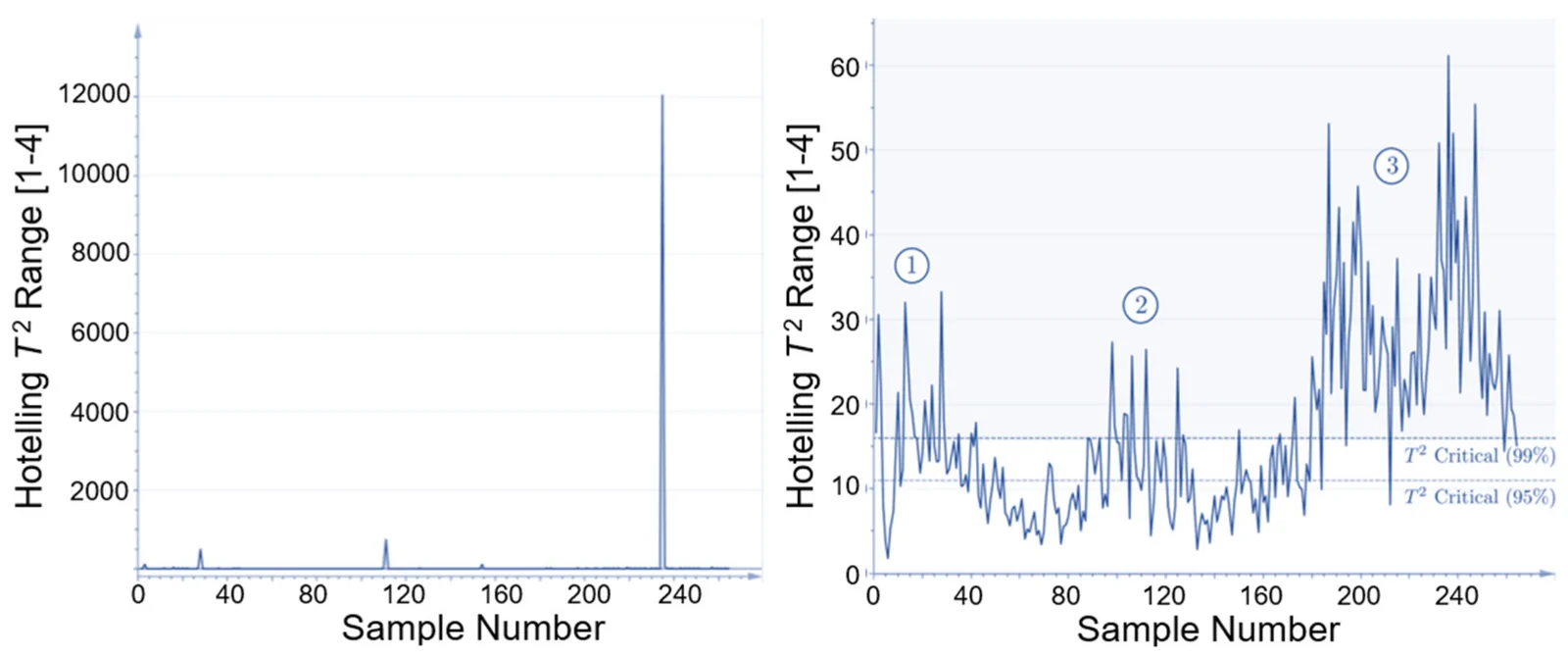
Hotelling *T*^2^ plots for: new batch, with 4 components and 3 historical batches (**left**), and new batch, again with 4 components but combining 50 in silico and 3 historical batches (**right**) [48].

The Hotelling *T*^2^ and squared prediction error (SPE) provided the control limits: the first detects shifts and deviations from normal operations, and the second quantifies the magnitude of variations in the data that cannot be explained by the postulated model [48]. Figure 37 presents a *T*^2^ chart for a new incoming batch based on the Low-N model (left), depicting large *T*^2^ values yet without control limits to flag atypical operation of concern. Employing in silico batch dataset generation (via GP-SSM) yields a richer profile (right), in which we can distinguish three separate areas outside the colour-lined control limits.

The conclusion is that the Large-N outperforms the Low-N model in explaining and predicting data variation; the former has higher statistical stability vs. the latter (due to its Law of Large Numbers/LLN-based assumptions in the BMP theory), and can credibly pinpoint three separate *alarm events* denoted as (1), (2) and (3) in Figure 37 (right), in which the signal surpasses both 95% and 99% *T*^2^ control limits, for several consecutive samples (indeed the right panel is far more indicative of *T*^2^ plots derived from batch operations).

A study by Jin et al. (2018) [49] presents a PCA-based bioprocess monitoring method for analysing abnormal variations often experienced in commercial mAb manufacturing, adversely affecting plant performance and consistency of final pharma product quality.

Their case study concerns Immunoglobulin G (IgG), which is the most abundant type of antibody in human blood and extracellular fluid (ca. 75% of serum antibodies); a potent remedy against bacterial and viral infections, it neutralises pathogens and binds to them for destruction by other immune cells. For such IgG plants, such abnormal variations are correlated with high lactate in recombinant CHO cells producing IgG, and lactate titres were also predicted therein via a nonlinear support vector machine (SVM) classifier [30].

**Figure 38:**
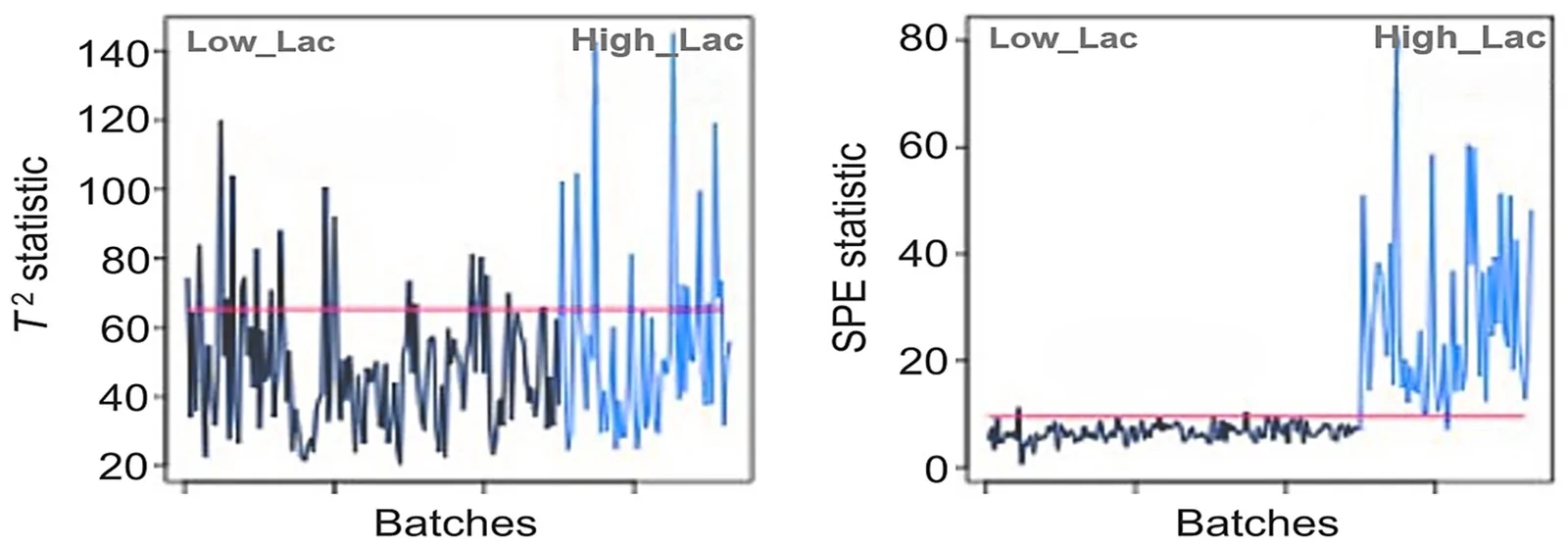
PCA monitoring results in principal space (**left**) and in residual space (**right**) as per [49].

Figure 38 presents a typical signal variation (the study used a dataset from 166 runs), observing that high- and low-lactate batches could not be intuitively (or unmistakably) separated in the original (principal) space. Hence, a latent space was then searched and determined, so as to separate them under reduced coordinates (PCA-based monitoring). Figure 38 elucidates how high- and low-lactate runs are inseparable in the principal (vs. the *T*^2^ limit), but differentiable in the residual space vs. the respective (SPE) control limit.

**Figure 39:**
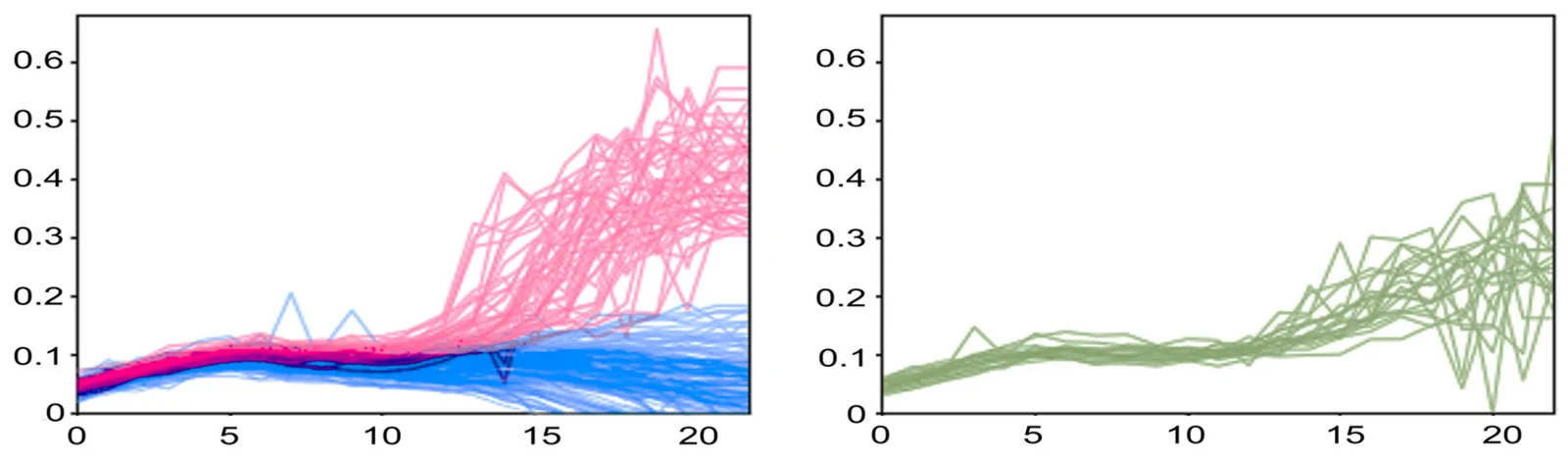
Classified high/low (**left**) and unclassified intermediate (**right**) lactate profiles from [50].

Jin et al. (2019) in a later study [50] further observed that the lactate concentration fluctuated widely near the end of batch runs, and did so even more for high-lactate ones. Due to a quasi-bivariate final lactate concentration distribution, it is easy to distinguish high- and low-lactate profiles, but also with many intermediates unclassified (Figure 39).

**Figure 40:**
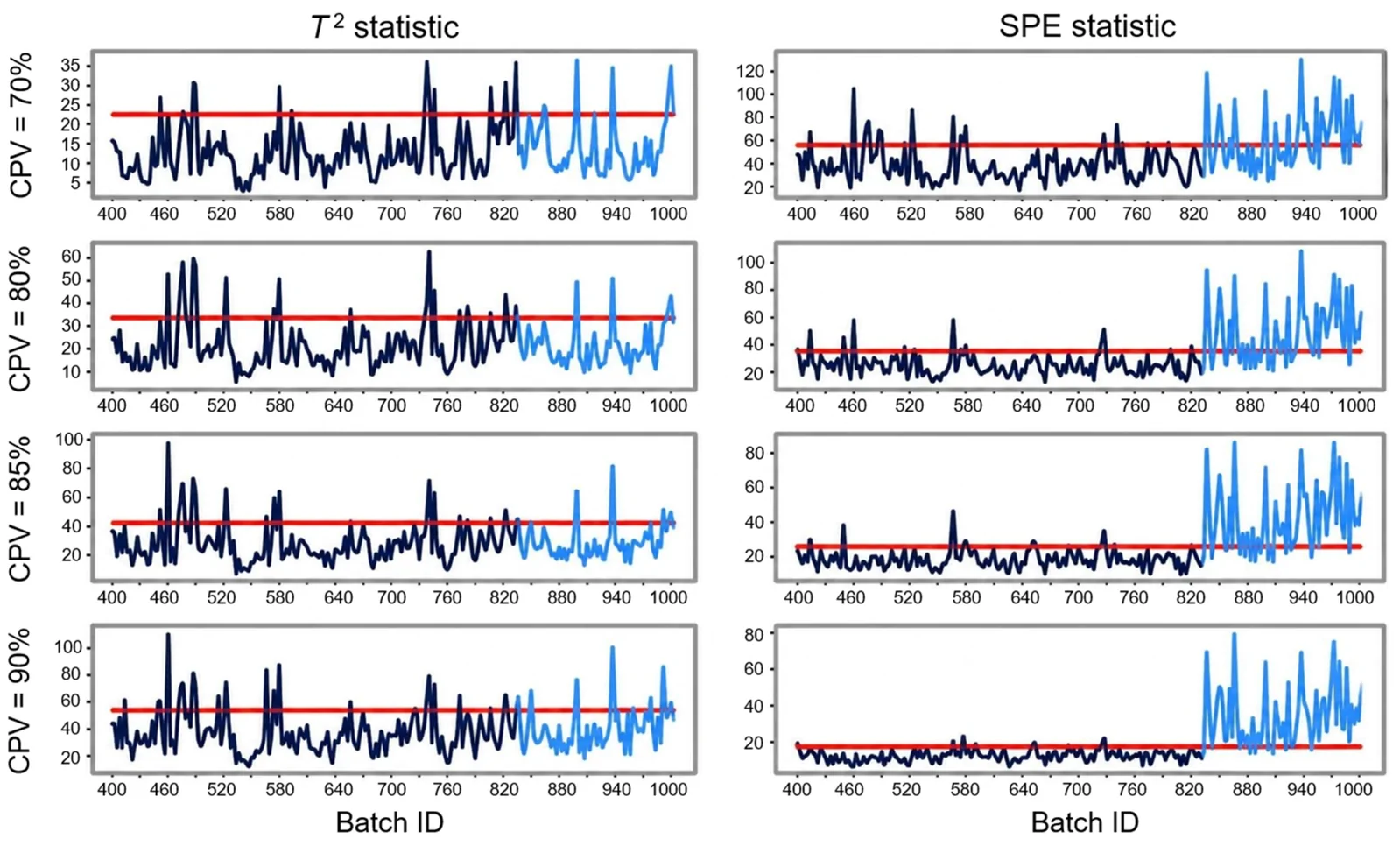
T^2^ (**left**) and SPE (**right**) plots for (top) 70%, 80%, 85% and 90% CPV, adapted from [50].

Figure 40 shows that using batch industrial production data of an 8-year period is ineffective and inconclusive in the PCA subspace: high- and low-lactate batches scarcely differ in terms of *T*^2^ values (left), whereas SPE is very effective in the residual subspace. High- vs. low-lactate batches were thus easily separated for most (if not all) cumulative proportion of variance (CPV) values, with the best distinction seen for CPV = 90% (right).

## 4. Downstream mAb Processing

The foregoing studies discuss AI/ML applications for upstream mAb manufacturing process modelling and optimisation only, within or near the bioreactors producing mAbs. Beyond that, AI/ML can improve many pivotal downstream operations (chromatographic separations, filtration, viral inactivation) for better quality control and plant sustainability.

**Figure 41:**
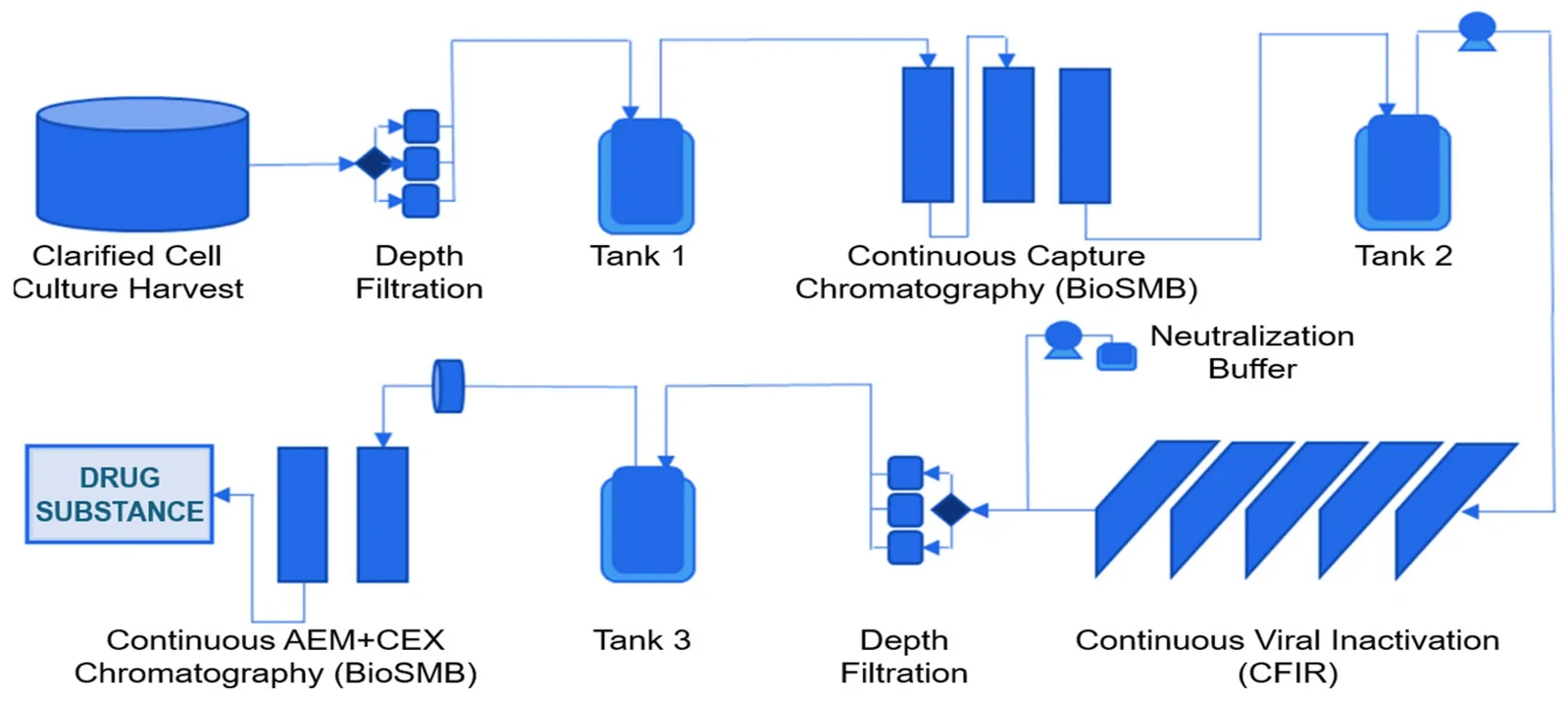
A process diagram for downstream continuous mAb manufacturing, adapted from [45].

Figure 41 is a process flow diagram for the downstream part of continuous mAb manufacturing: after the bioreactor and cell culture harvest, the resulting mAb solution undergoes protein A chromatography, viral inactivation, filtration [51], anion exchange membrane (AEM) and cation exchange (CEX) chromatography before final formulation.

The study by Nikita et al. [45] details ML application to develop prediction models for capture and polishing chromatography steps, comparing 5 different ML algorithms: (i) deep neural networks (DNN), (ii) support vector regression (SVR), (iii) decision trees (DT), (iv) random forest regression (RFR), and (v) gradient boosting regression (GBR). Intra-batch real-time data were compiled for key variables (conductivity, pH, UV sensor).

A brief overview of fundamental concepts for some of the methods in [45] follows. The DNN implementations rely on several processing layers that learn based on process conditions *x*_i_, predicting parameters *w*_i_ and targets *y*. The nonlinear output function is:

(14) $$y=\;\alpha (x_{1}w_{1}+x_{2}w_{2}+x_{n}w_{n}+b)$$

where *α* is the activation function, *x*_i_ are input values, *w*_i_ are the weights, and b is a bias.

**Figure 42:**
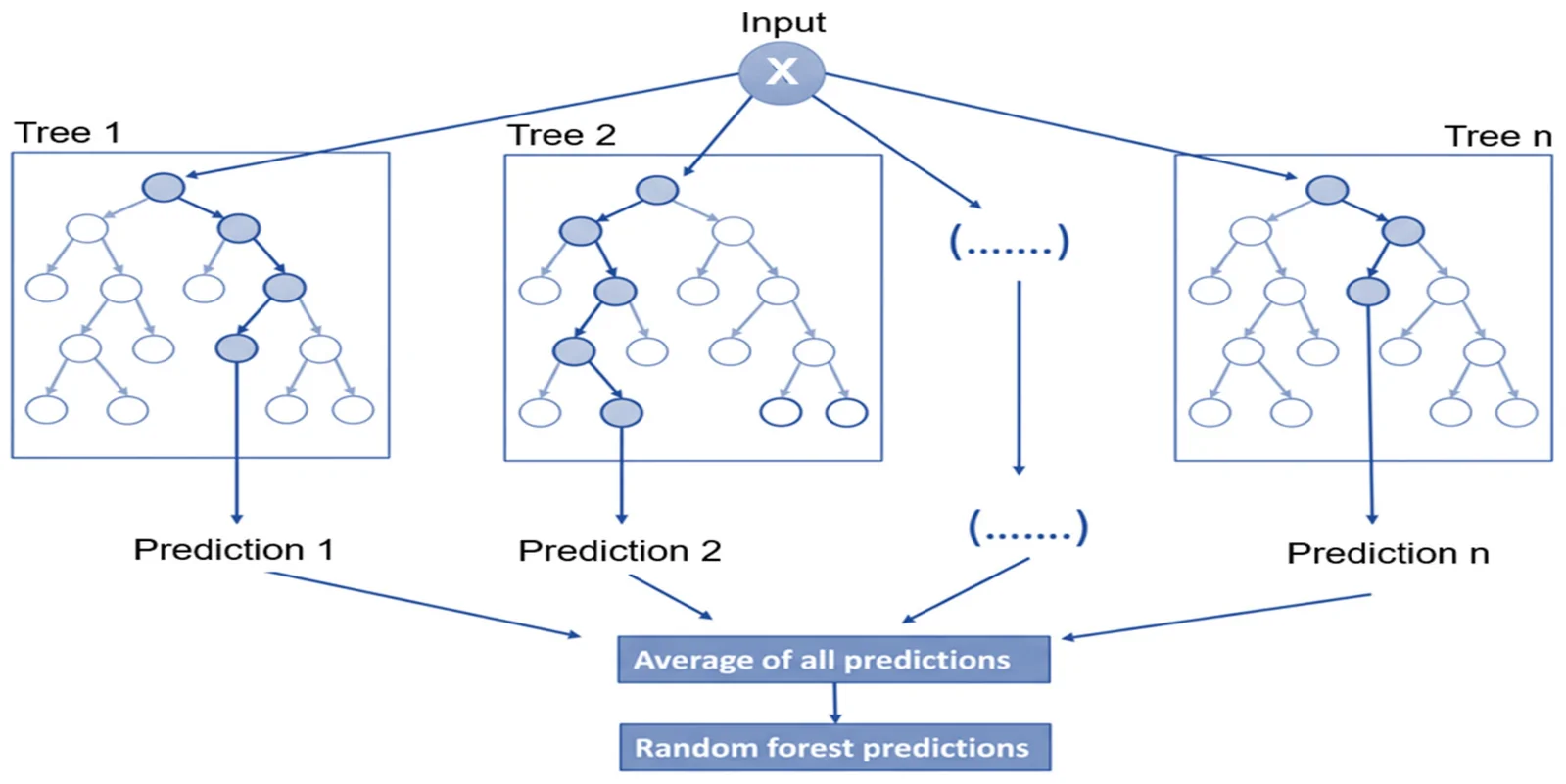
Random Forest Regression (RFR) for ML based response prediction, adapted from [45].

**Figure 43:**
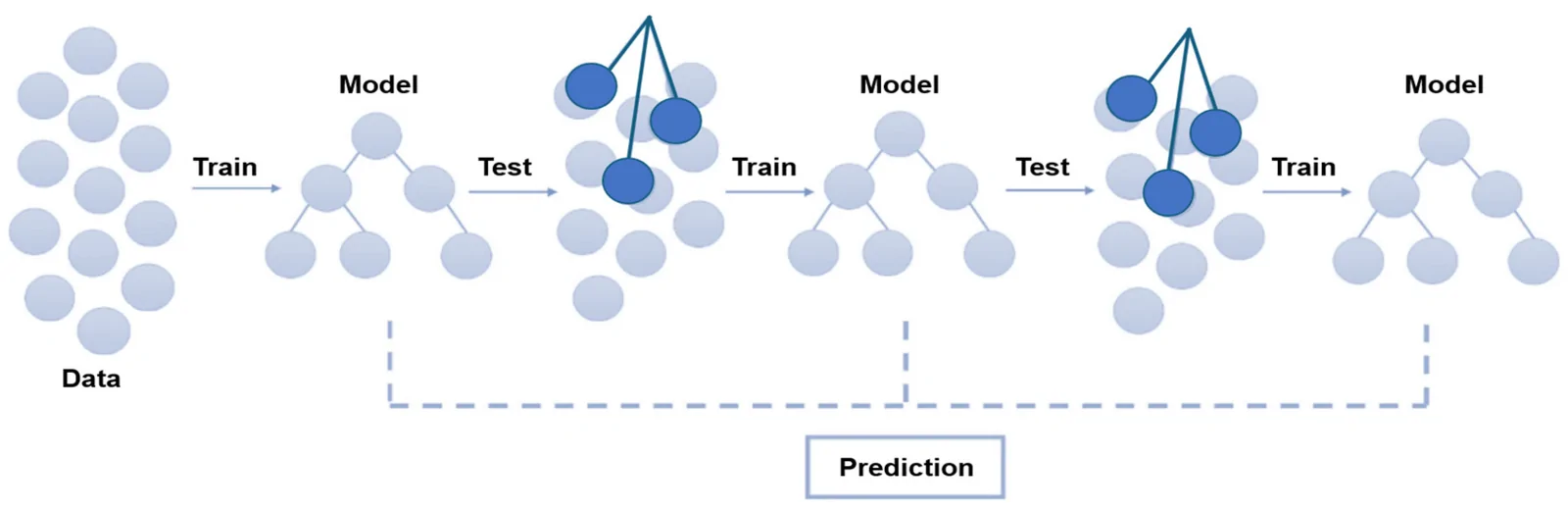
Gradient Boosting Regression (GBR) for ML-based trend prediction, adapted from [45].

Figure 42 and Figure 43 show RFR and GBR principles: the first averages predictions from many diverse structures, and the second relies on successive training/testing cycles [45].

Model fidelity is measured by the root mean square error (RMSE) and the coefficient of determination (*R*^2^), close to 1 for reliable regressions (training datasets are excluded):

(15) $$R^{2}=1-\frac{\sum_{i}{\left(y_{i}-f_{i}\right)}^{2}}{\sum_{i}{\left(y_{i}-\bar{y}\right)}^{2}}$$

where *y_i_* are experimental data, $\bar{y}$ is their average, and *f_i_* are the model predictions.

**Figure 44:**
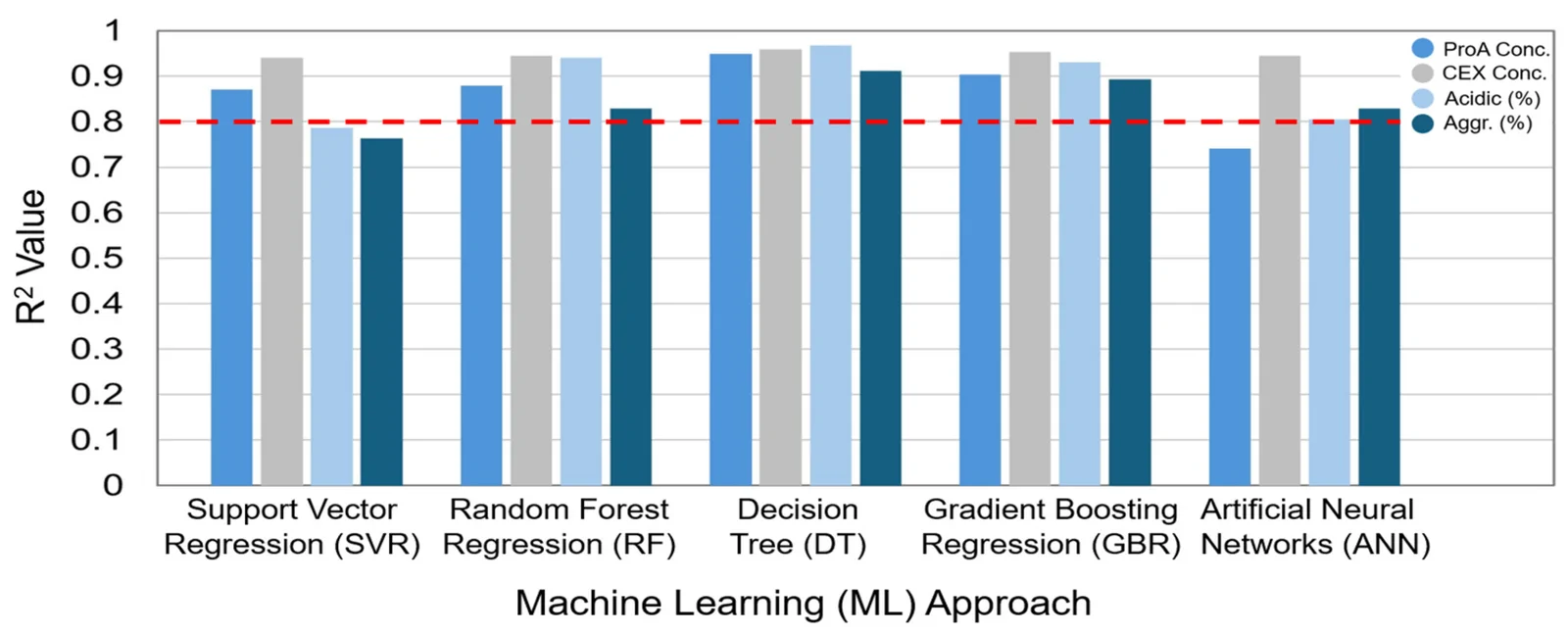
Comparison of *R*^2^ values for key observables and different ML approaches; data by [45].

Figure 44 shows that all methods achieve adequately high accuracy for most observables (*R*^2^ > 0.8), exception SVR for aggregate content (first group, last bar), but also DNN for mAb elute concentration of Protein A chromatography (last group, first bar). Akaike (AIC) and Bayes (BIC) information criteria rule out GBR use: AIC/BIC values are 5–65% higher than tree-based models, and low criteria values maximise fidelity [45].

**Figure 45:**
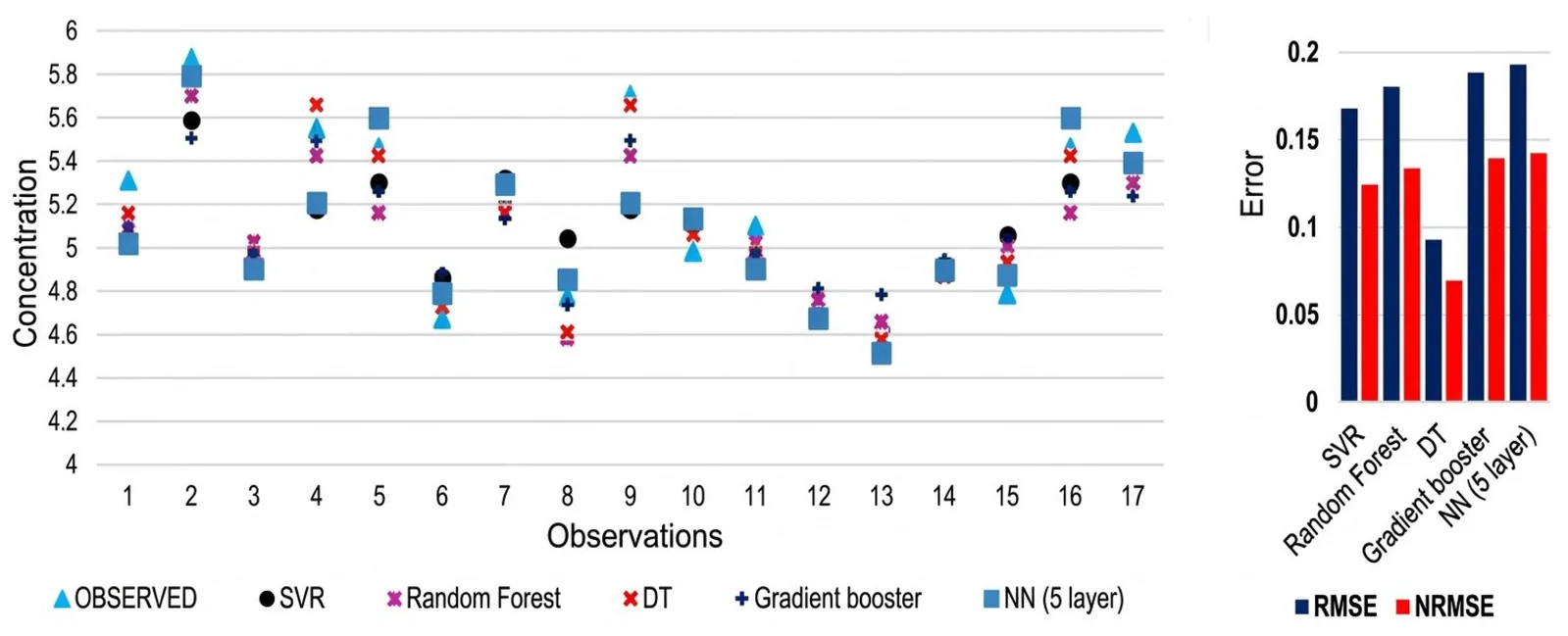
AI/ML performance for eluted mAb prediction in protein A chromatography, from [45].

Figure 45 shows all model predictions vs. experiments (left) and error metrics (right). DT and SVR emerge as most accurate (minimal RMSE), with GBR and NN least reliable; RFR is superior to DT and other algorithms for small datasets (due to reduced CPU time), achieving 4.76% max. error in mAb elute level prediction from protein A chromatography.

## 5. Biopharmaceutical Process Scale-Up

AI can play a crucial role in biopharmaceutical process scale-up, which belabours the transition from laboratory-scale processes to larger production scales. AI algorithms can analyse large datasets generated during laboratory-scale processes and identify patterns, correlations, and process parameters influencing product quality and yield. Data-driven approaches can help us develop accurate models and extrapolate to larger sizes, to predict (via historical data and process knowledge) how bioprocesses behave when scaled up. By simulating various scenarios, AI can help identify potential challenges and optimise scaling-up, reducing the need for costly and time-consuming trial-and-error experiments.

**Figure 46:**
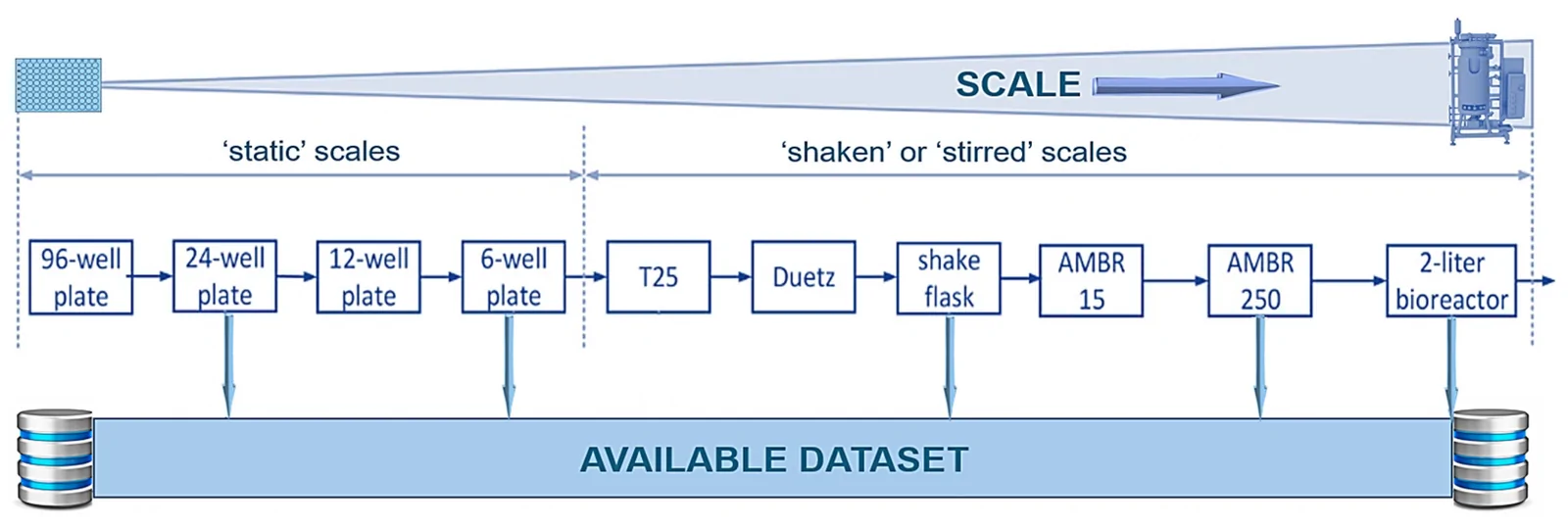
Bioprocess scale-up for mAb-producing cell line generation: units and data, from [27].

Facco et al. (2020) used multivariate statistics, data analytics and pattern recognition for biopharma process scale-up in mAb production (Figure 46) [27], achieving cell line screening and performance prediction for selecting static, shaken or agitated bioreactors.

Data-driven models used in [27] make use of multivariate statistical methodologies and standard pattern recognition techniques. Principal component analysis (PCA) is thus deployed for data processing; it summarises the information available in a dataset *X* as:

(16) $$X=\sum_{a=1}^{R}t_{a}{P_{a}}^{T}=\sum_{a=1}^{A}t_{a}{P_{a}}^{T}+\sum_{a=A+1}^{R}t_{a}{P_{a}}^{T}=\sum_{a=1}^{A}t_{a}{P_{a}}^{T}+E$$

where *R* is the rank of *X*; *P_a_* is the [M × 1] loading vector and *t_a_* is the [N × 1] score vector for the ath PC; superscript ^T^ is the transpose operator; *E* is the [N × M] residual matrix (to be minimised in least-squares sense), and *A* is the number of PCs that need be determined.

Two statistical modelling tools, Partial Least Squares (PLS) and Joint-Y PLS (JY-PLS) were used for predictive analysis: these regression methodologies determine the direction of maximum variability in a set of regressors (inputs) *X* [N × M] that are most predictive for a given set of responses (outputs) *Y* [N × U], according to the next matrix equations:

(17) $$X=TP^{T}+E$$

(18) $$Y=TQ^{T}+F$$

(19) $$T=XW^{*}$$

where *T* is the [N × A] score matrix, *P* [M × A] and *Q* [U × A] are the loading matrices for inputs *X* and outputs *Y*, E and *F* are the [N × M] and [N × U] residual matrices of *X* and *Y*, respectively (to be minimised in least-squares sense), and W* is the [M × A] weight matrix.

**Figure 47:**
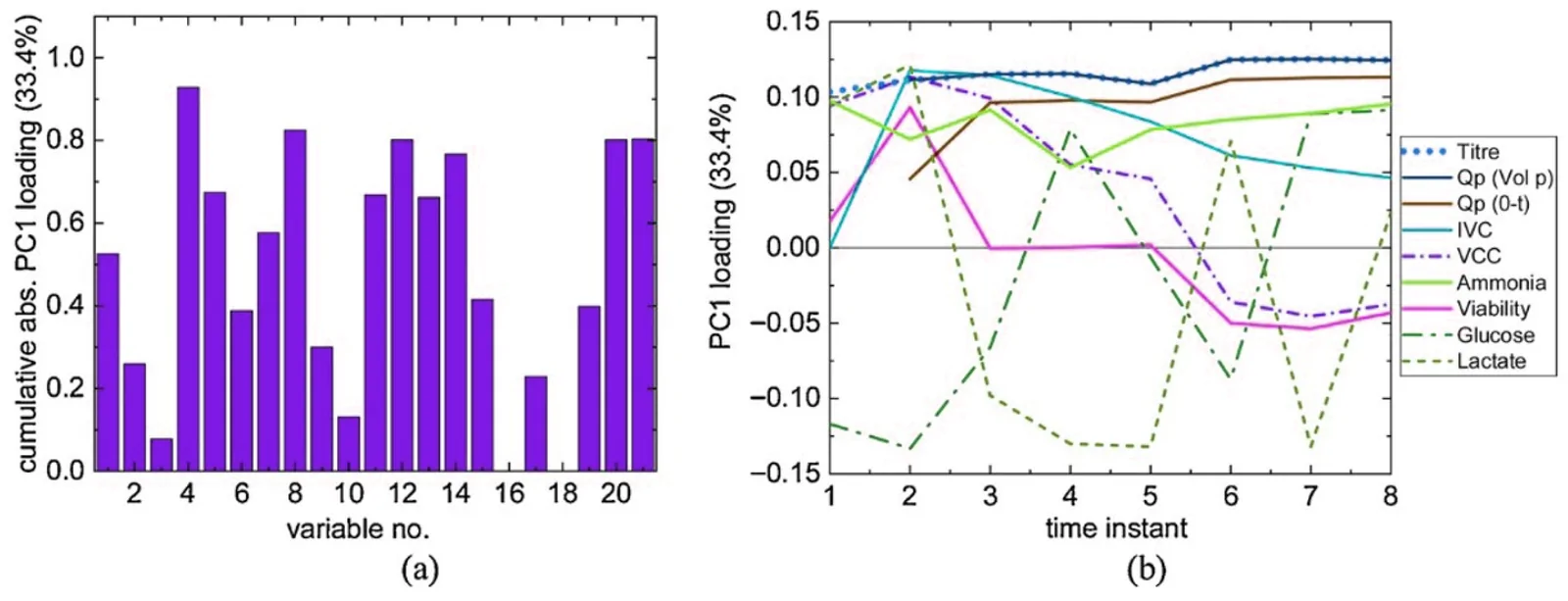
MPCA (**a**) time-cumulated absolute loadings, and (**b**) key state variable profiles, by [27].

The study considered 4 PCs: Figure 47a shows PC1 load cumulative absolute values of all measured variables across all batches, and Figure 47b proves the model correctly captures titres, and a pronounced anti-correlation of glucose (cell nutrient) vs. lactate. The authors of [27] also report similar results from a dynamic analysis of the 2 L bioreactor, implying the variables affecting variability among batches are the same across both scales.

The PLS (local) model uses available data (metabolites, pH, O_2_ transfer rates), while the JY-PLS (global/shared) model uses data from all other scales except for the predicted.

**Figure 48:**
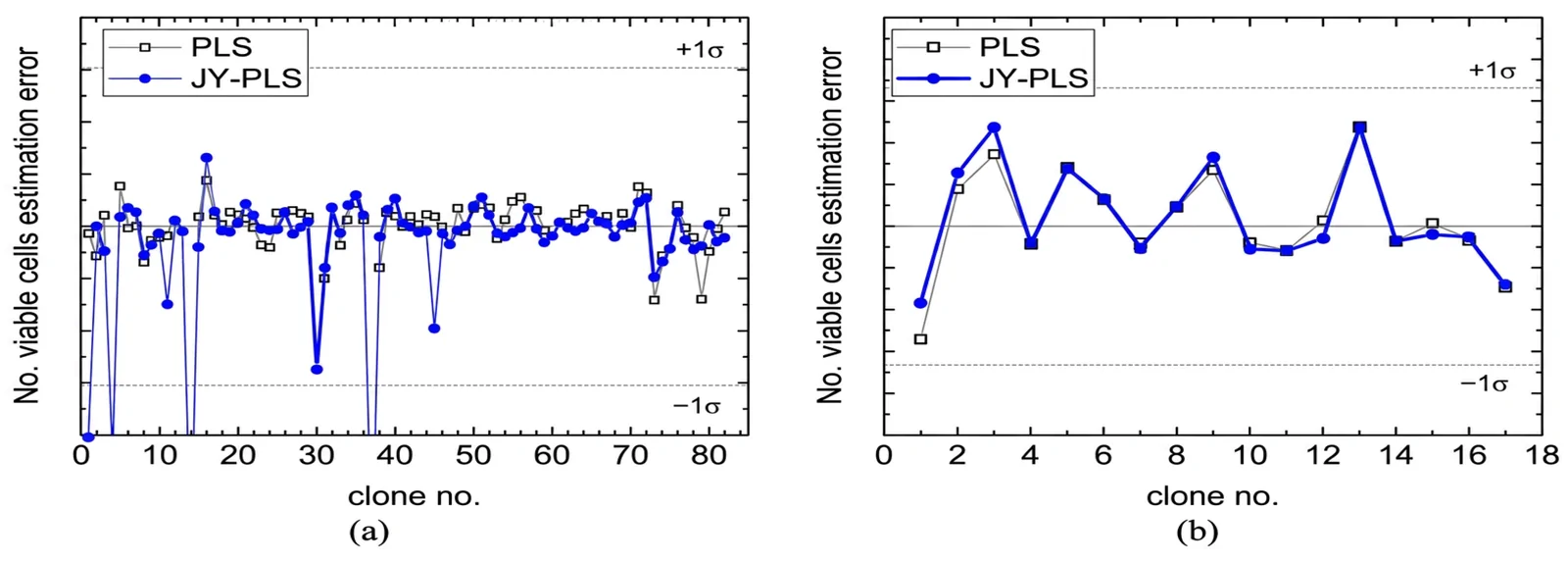
Viable cell estimation error: (**a**) 6-well scale (best), (**b**) late T25 scale (worst), from [27].

Table 8 summarises the validation and performance for both modelling approaches. For PLS and JY-PLS and all bar one scale, the determination coefficient *R^2^* is at least 0.94, indicating reliable estimation. The PLS model is more accurate than JY-PLS in all bar one cases; the best and worst overall validation results at two scales are shown in Figure 48, showing all estimation errors well within a standard deviation of measured data (μ ± 1σ).

**Figure 49:**
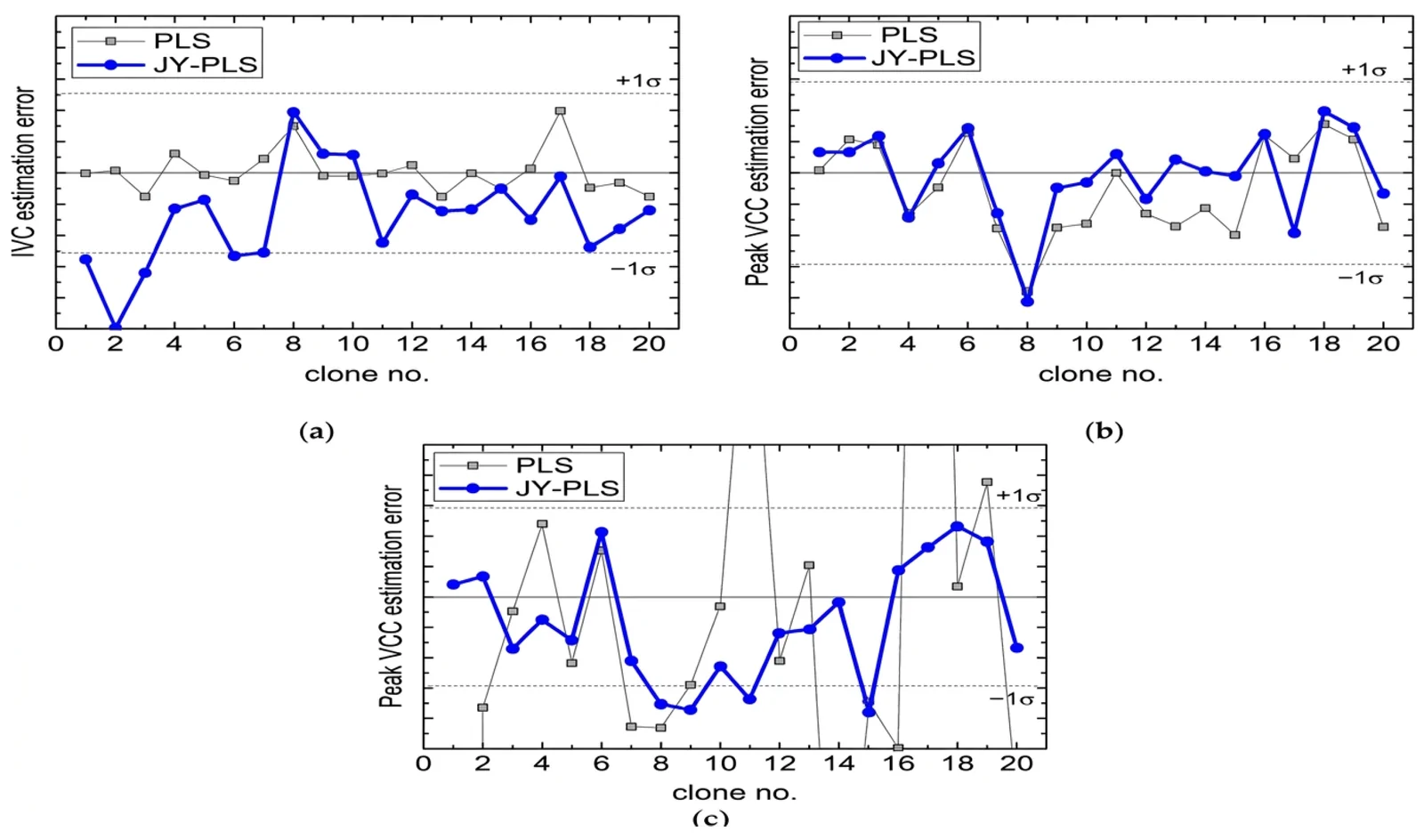
Estimation error for: (**a**) IVC and (**b**,**c**) Peak VCC estimation at shake-flask scale, for which (**b**) all the calibration clones and (**c**) only 5 calibration clones are used for computations - from [27].

Figure 49a portrays Integral of Viable Cells (IVC) estimates from shake-flask data; PLS is good, but JY-PLS (inclusion of other-scale data) worsens estimation performance. Figure 49b, however, shows that the peak viable cell count (VCC) trend is very different: JY-PLS is much better than PLS herein, indicating that information from other scales can improve estimation accuracy, a clear benefit to correlation structure at the particular size. The JY-PLS advantage is greater if only a few samples are available at shake-flask scale.

Figure 49c depicts Peak VCC estimation also, but for only 5 samples available now: PLS accuracy declined enormously, while JY-PLS performance deteriorated very slightly. The conclusion is that data scarcity at a given scale can be mitigated by exploiting valuable information embedded in data at other scales, reducing R&D risk in cell culture selection.

**Figure 50:**
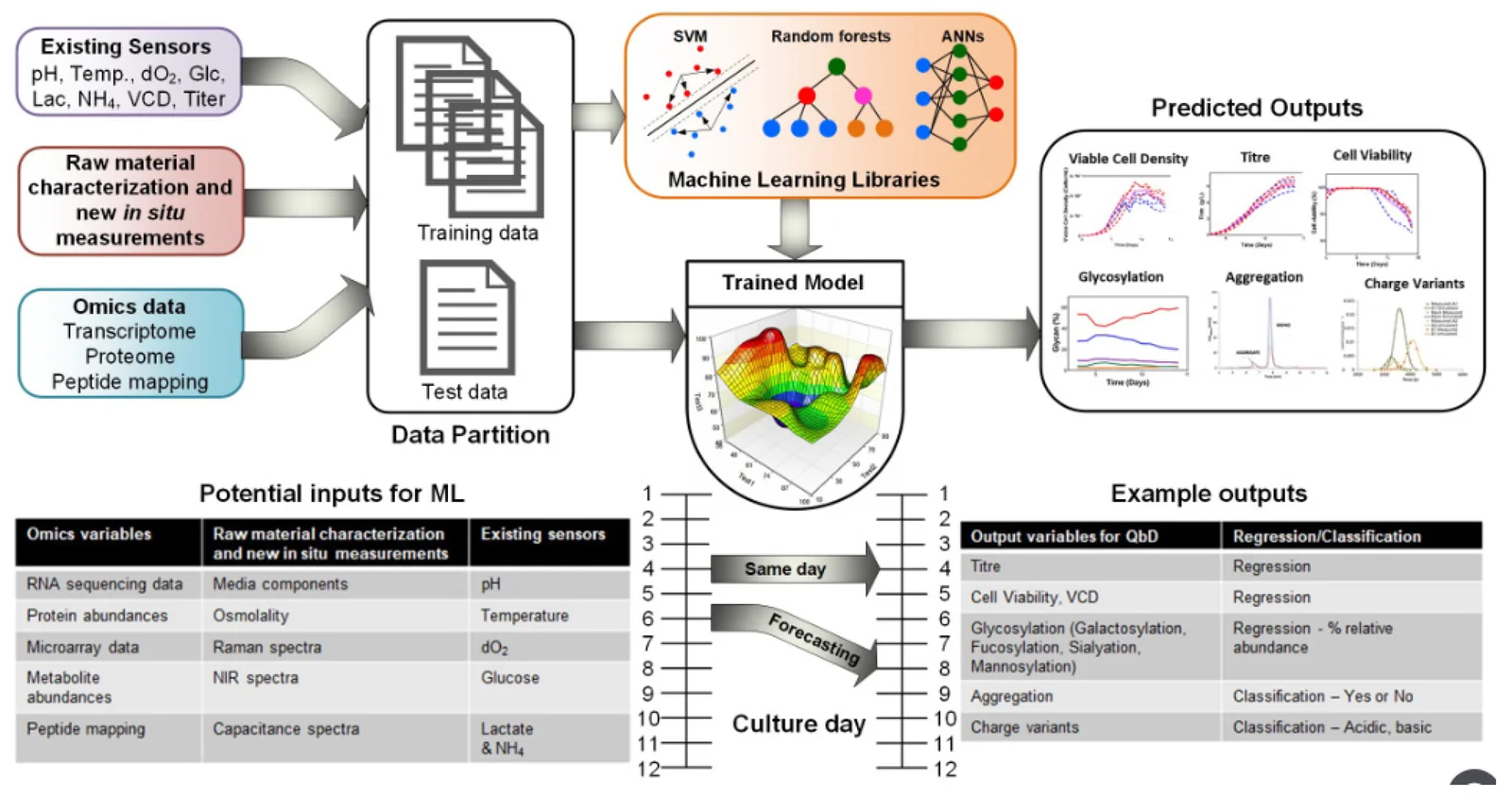
A superstructure describing ML modelling and multiple QbD applications, from [52].

A review on Quality by Design (QbD) via ML by Walsh et al. [52] describes the role of statistical modelling in biomanufacturing, showing how certain ML tools can analyse bioprocess datasets, to accelerate systematic QbD implementation in industry (Figure 50). Critical process parameters (CPP) and quality attributes (CQA) are defined, followed by a comparison of multivariate data analysis (MVDA) vs. supervised and unsupervised ML.

## 6. ML for mAb Property and Quality Control

Beyond AI/ML applications in plantwide mAb manufacturing, ML methods [53,54,55,56,57,58,59,60,61,62] emerge as useful in predicting, calculating and controlling specific physical properties of mAbs: the next sections describe a variety of successful ML implementations to this end.

### 6.1. Viscosity

Viscosity is one of the pivotal mAb physical properties determining their final market value: mAb solutions exhibit a significant viscosity increase with increasing mAb titres, which in turn complicates manufacturing processes, limiting options for product delivery as the underlying molecular basis of rheological behaviour is still not widely studied [53].

Lai et al. (2021) [54] measured concentration-dependent solution viscosity of 27 FDA-approved mAbs, in the 50–200 mg.mL^−1^ range (pH = 6.0, 10 mM histidine-HCl buffer). Predicted net charges and special charge map (SCM) scores were separately used in order to classify experimental viscosity data, and then ML algorithms were applied to search for molecular descriptors for those cases that accurately separated high and low viscosity. A high viscosity index (HVI) was used as a metric for rapid (low vs. high viscosity) mAb screening, and a decision tree model based on the mAb net charge and HVI was proposed.

**Figure 51:**
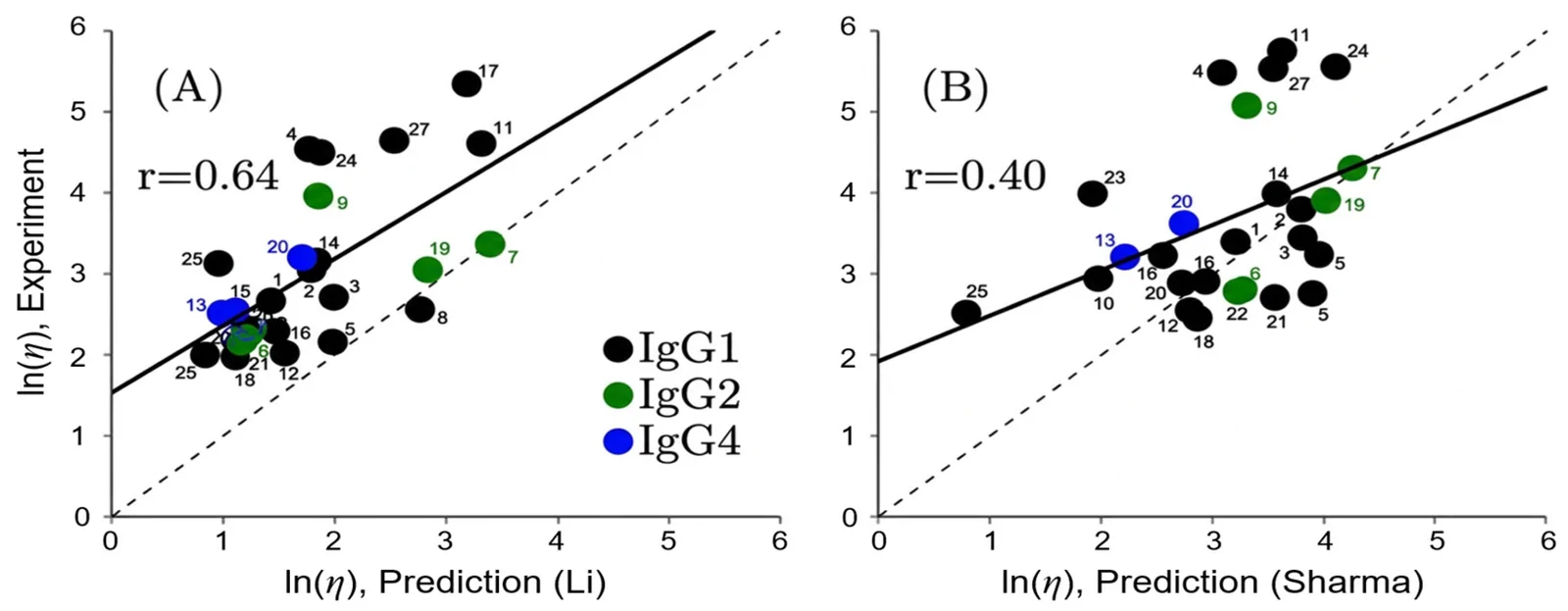
Comparison of mAb viscosity (*η*) predictions of two models (**A**,**B**) vs. exptl. data, from [54].

A comparative analysis of viscosity predictions for immunoglobulin G variants [54] has evaluated the models proposed by Li et al. (2014) [56] and Sharma et al. (2014) [57]. Figure 51 summarises viscosity predictions based on these models: solid and dashed lines therein denote the least squares regression and the identity (diagonal) lines, respectively.

The first model [55] predicted the viscosity of 27 proprietary mAbs at 150 mg.mL^−1^ (pH = 5.8, 20 mM histidine-HCl buffer), underestimating most experimental data (*r* = 0.64). The second model [56] pursued viscosity prediction for the same mAbs at 180 mg.mL^−1^ (pH = 5.5, 200 mM arginine-HCl buffer), at worse correlation to experimental data (r = 0.4); on the latter, it is noted that solution condition mismatch may affect model performance.

**Figure 52:**
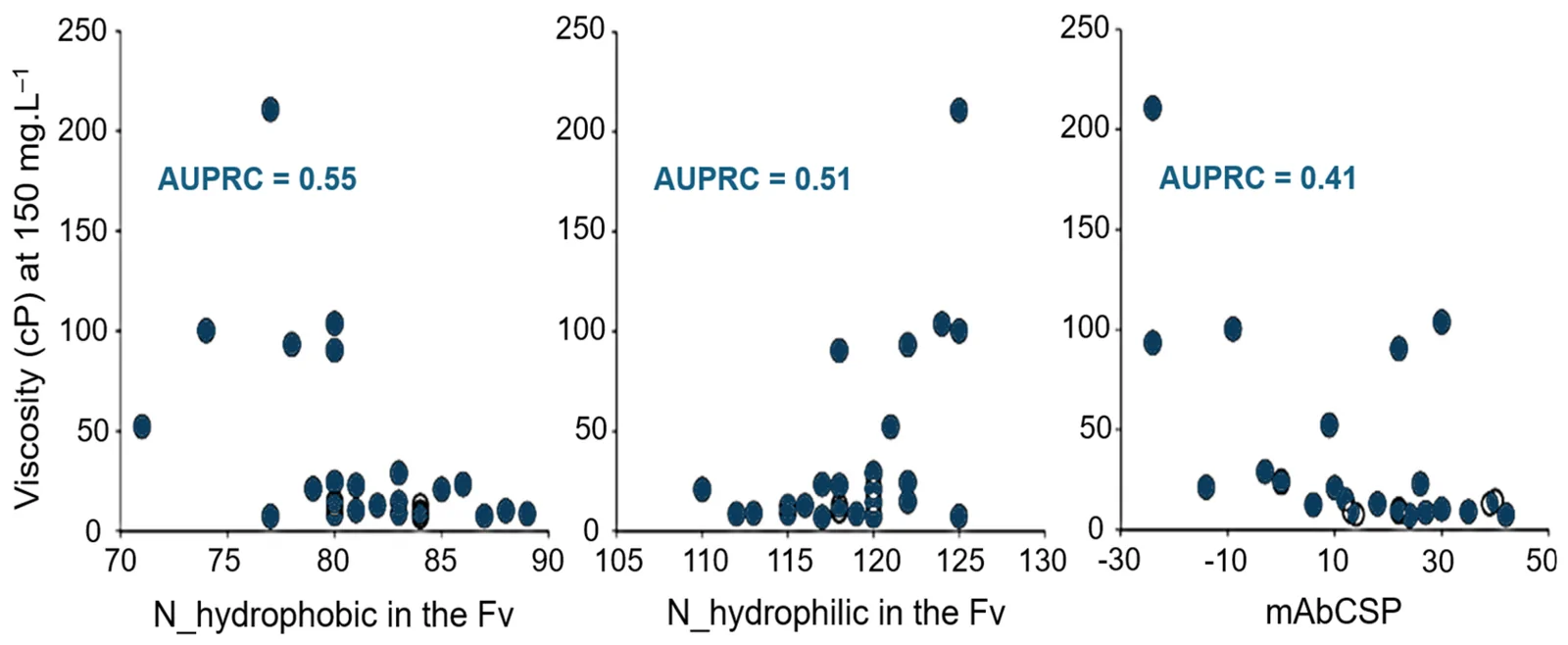
Relationship between viscosity and top three (max. AUPRC) mAb features, data by [54].

Another ML study of viscosity prediction [54] partitioned 27 mAbs into training and test sets for model evaluation and feature selection, including key molecular descriptors (e.g., charge, hydrophobic and hydrophilic properties) which affect rheological behaviour. The area under the precision-recall curve (AUPRC) was chosen as the accuracy metric, so the top 3 features (max. AUPRC) found are the hydrophobic and hydrophilic residues in Fv regions (N_hydrophobic, N_hydrophilic) and the charge symmetry parameter (CSP); achieving 86%, 83% and 81% accuracy, respectively, for the AUPRC values of Figure 52.

Authors note high-viscosity mmAbs are richer in hydrophilic (poorer in hydrophobic) residues, concluding (via charge analysis) that mAbs of high or low net charge have lower viscosity due to the resulting higher repulsive interactions between proteins, or strong attractive interactions that lead to larger cluster formation and opalescence. Conversely, the rheology of mAbs with medium net charge is governed by short-range interactions.

Lai et al. (2022) [55] later predicted and measured mAb aggregation rates and viscosity at 45 °C and 150 mg.mL^−1^ for a set of 20 AstraZeneca (preclinical and clinical-stage) mAbs. The collection included 18 IgG1- and 2 IgG4P-subclass products; accelerated aggregation tests (at pH = 6.0, using a 20 mM histidine-HCl buffer, for 2 weeks) provided experimental data for essential comparisons vs. mAb aggregation predictions, to evaluate ML model fidelity via the leave-one-out cross-validation (LOOCV) method for various regression models [55]. Molecular Dynamics (MD) simulations provided full-length antibody features (specific aggregation-inducing sequences) for ML model development.

**Figure 53:**
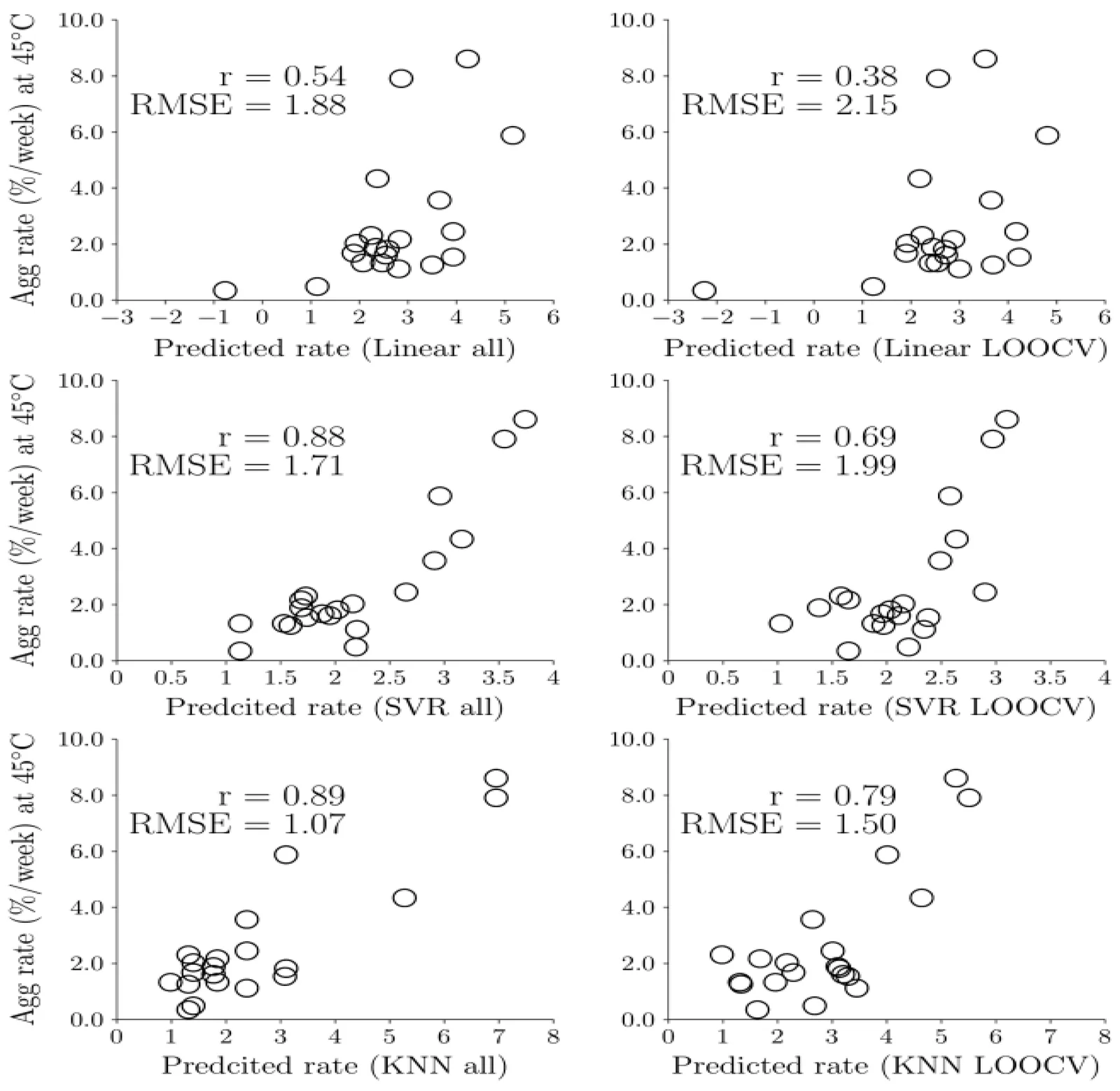
Correlation coefficients for the best two-feature linear, SVR and kNN models [55].

Figure 53 shows the comparison of predicted vs. experimental aggregation rates and fit statistics (RMSE and linear correlation coefficients, r) computed via the best two-feature combination from three regression models: linear, support vector regression (SVR), and k-nearest neighbours (kNN); the latter achieved both max(r) = 0.89 and min(RMSE) = 1.07. Later advances explored concentration-dependent viscosity behaviour [58,59], viscosity reduction [60], and targeted high-concentration mAb product stability improvement [61].

### 6.2. Thermal Stability

Thermal stability is a critical feature in determining R&D prospects of therapeutics. A recent thermostability study by Harmalkar et al. (2023) [62] addressed *multispecific biologics* (*msAbs*), which can successfully engage multiple targets (unlike mAbs that only bind to a single target): msAbs feature a single-chain variable fragment (scFv), a fusion protein of the variable regions of heavy (*V_H_*) and light (*V_L_*) chains of immunoglobulins, connected with a short peptide linker (ca. 10–25 amino acids). A key challenge hampering the market potential of msAbs is their poor physical properties vs. conventional mAbs, because of scFvs, and the relatively poor product stability in case of varying temperatures.

The study [62] employed DL methods to extract thermal stability characteristics from experimentally generated scFv sequences, then screened them vs. temperature sensitivity. The unsupervised CNN predictive potential for thermostability was tested via pre-trained language models (PTLMs), and SL was subsequently applied to train CNN architectures.

**Figure 54:**
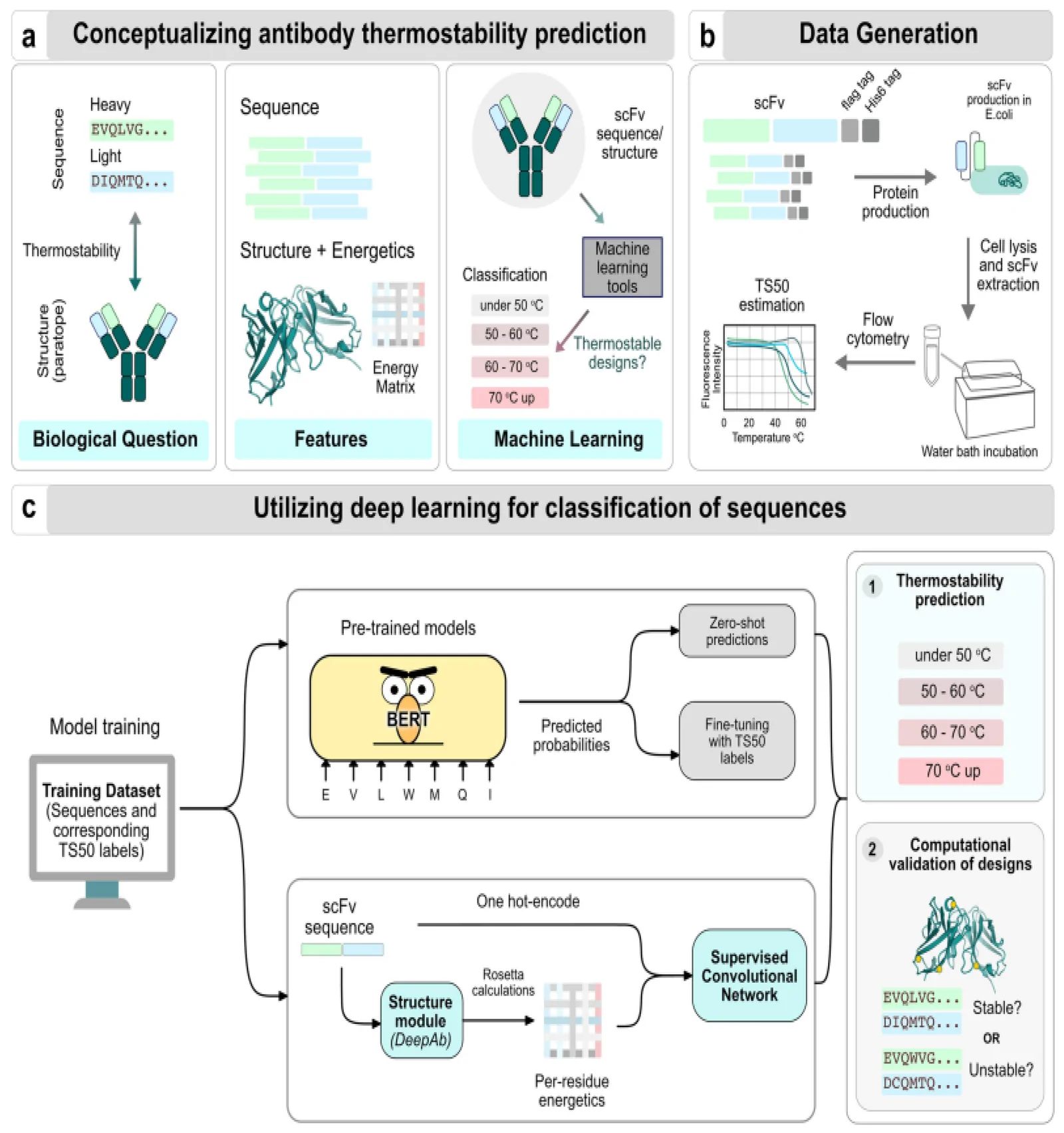
Thermostability (**a**) prediction, (**b**) data generation, (**c**) DL training for classification [62].

Figure 54 shows key biological challenges to predict antibody thermostability from sequences, e.g., identifying particular sequences for thermally stable structure formation. Both regression and classification were used to predict thermal stability via temperature measurements (TS50) for 2700 scFv sequences. For the first, absolute TS50 data were used; for the latter, TS50 data [63,64] were divided into four bins, to be used with a pre-trained language model (PTLM), and with a supervised CNN with Rosetta energetic features [65].

**Figure 55:**
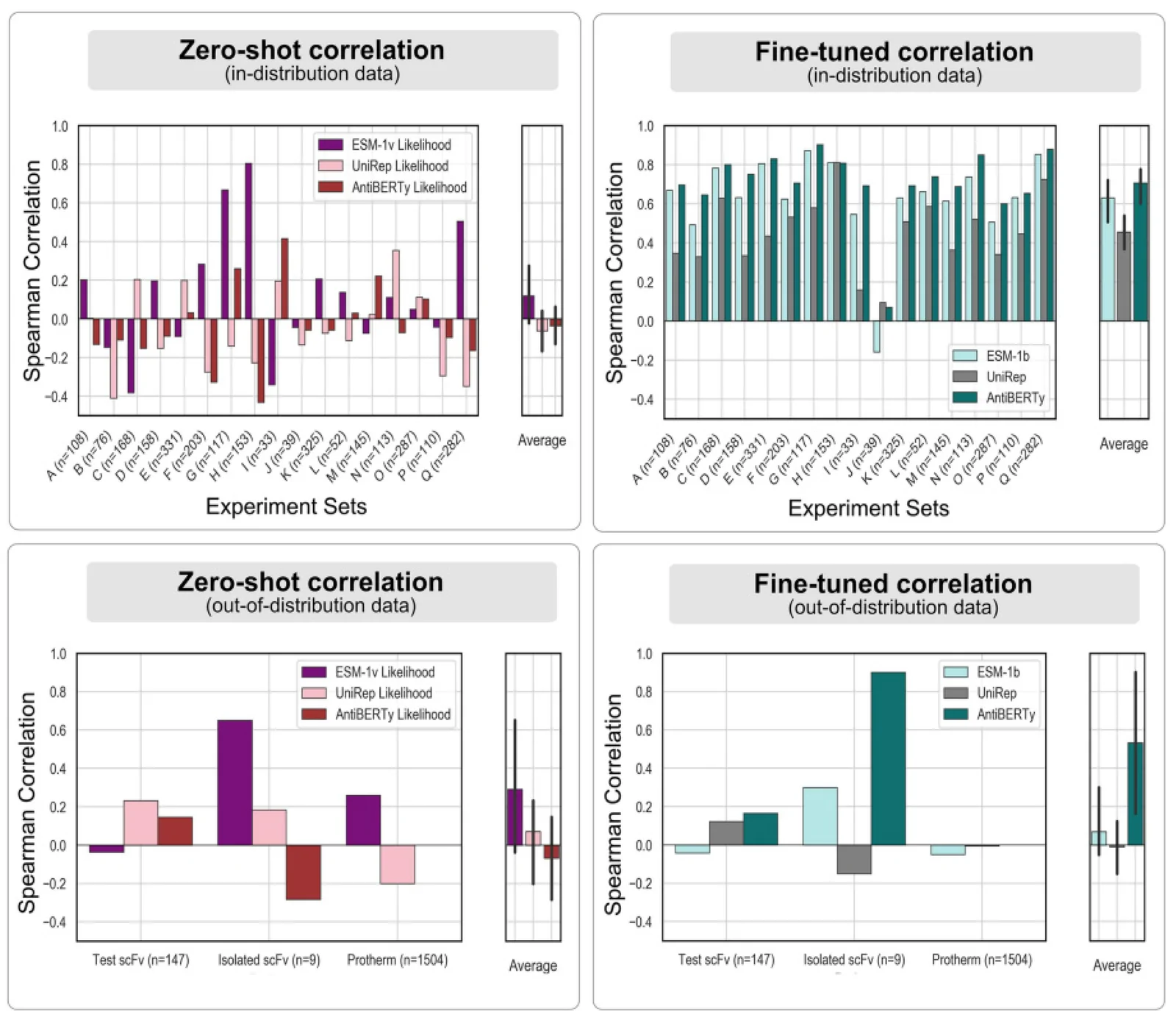
Fine-tuning improves the accuracy of ML models (**right**) vs. zero-shot ones (**left**) [62].

The ML models received enriched information (including thermodynamic features), unlike conventional ML approaches relying on sequence or structural coordinates only. The conclusion is that fine-tuning pre-trained sequence models with thermostability data can greatly improve their classification performance vs. zero-shot predictions (Figure 55).

**Figure 56:**
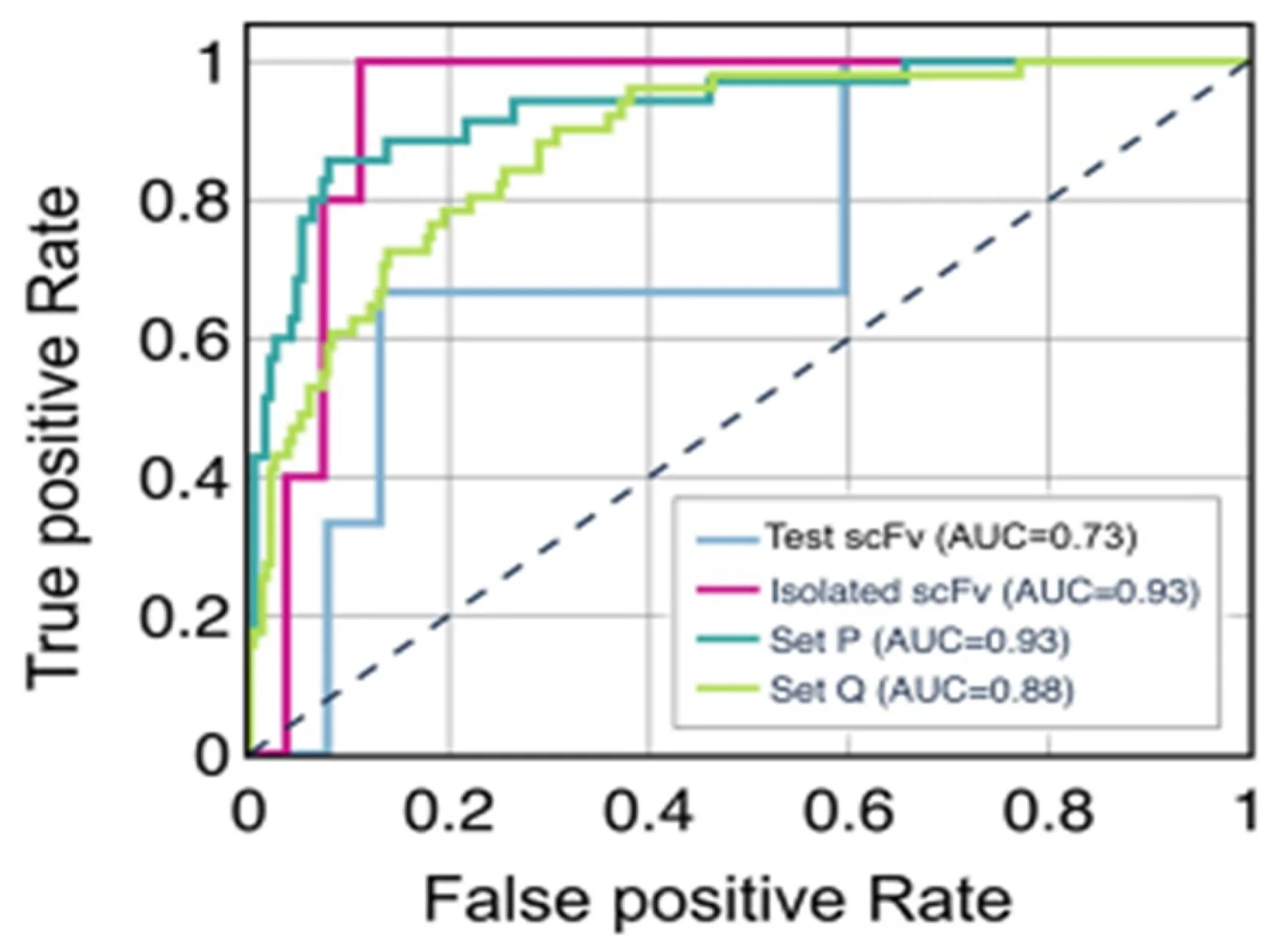
ROC curve demonstrating classification of test sequences for the AUC > 70 bin, by [62].

Figure 56 analyses SL model performance plotting receiver-operating-characteristic (ROC) curves derived from the prediction of the 70-up bin with the energetics-only model. The smaller sample sizes of test scFv and isolated scFv curves explain their few (3) kinks.

Data from thermal aggregation experimental studies by Koenig et al. (2017) [63] and Warszawski et al. (2019) [64] served to evaluate whether CNNs trained on TS50 data can provide insight into temperature-specific features (e.g., thermal aggregation), and if they can differentiate between thermally enhancing vs. thermally hampering mutations.

**Figure 57:**
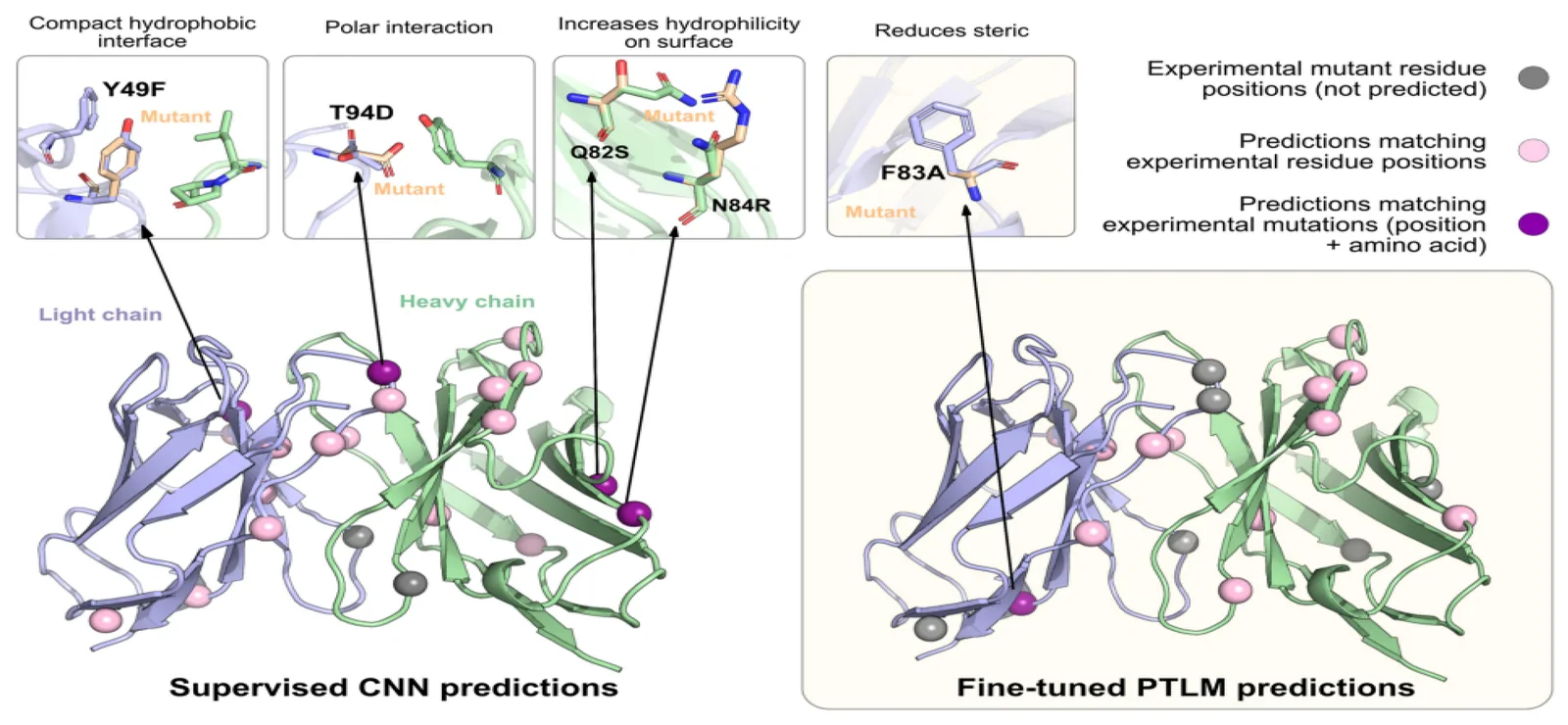
Computational deep mutational scan of mAb fragment agrees with exptl. data [62].

Figure 57 compares both models’ predictions vs. experimental thermostable mutants for heavy and light chains, revealing the performance of both supervised CNN and PTLM.

Spheres indicate experimentally validated mutants improving *T*_m_; pink indicates ML predictions matching experimental residue positions (but different amino acid mutation), purple denotes ML predictions matching both experimental residue positions and amino acid mutations, and grey indicates mutations not observed in computational predictions.

### 6.3. Characterisation

Various stresses during upstream/downstream mAb (and other therapeutic protein) manufacturing generate subvisible particle populations [66,67]: Greenblott et al. (2022) [66] used CNN to identify and classify particle generation root causes by subjecting three different mAbs (from formulations) to common manufacturing stresses, as per Table 9. The particle population generated by stressing was probed, indicating that CNN analyses are sensitive not only to the applied stress but also to the mAb type and buffer conditions.

ML models trained on particle images created with one mAb-buffer system cannot be accurate for root cause analysis if applied to particles generated by other such systems. A lever-rule analysis of CNN-derived fingerprints was employed in that study [66] to quantitatively characterise the composition of mixtures comprising various particle types. The FIM particle analysis method of [66] advances earlier work by Calderon et al. (2018) [67] relying on RGB preprocessing to portray temporal evolution of particle fingerprints.

**Figure 58:**
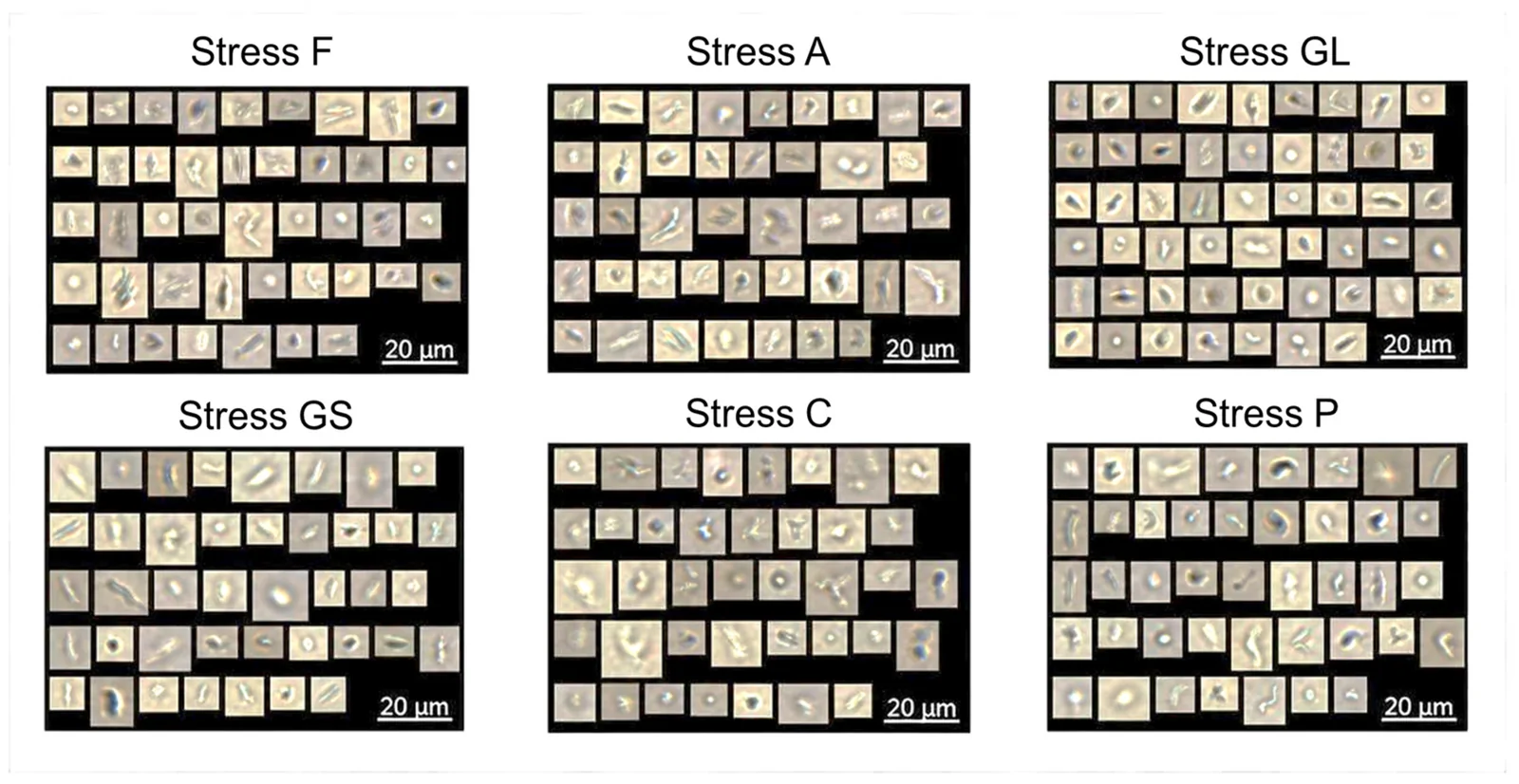
A collection of FIM particle images subjected to six stresses (Table 9), adapted from [66].

Flow imaging microscopy (FIM) analyses digital images of particles [66,67] from mAb formulations, e.g., with a FlowCAM^®^ VS instrument (Fluid Imaging Technologies). Figure 58 is an FIM image collection from mAb1 samples subjected to six stress conditions.

**Figure 59:**
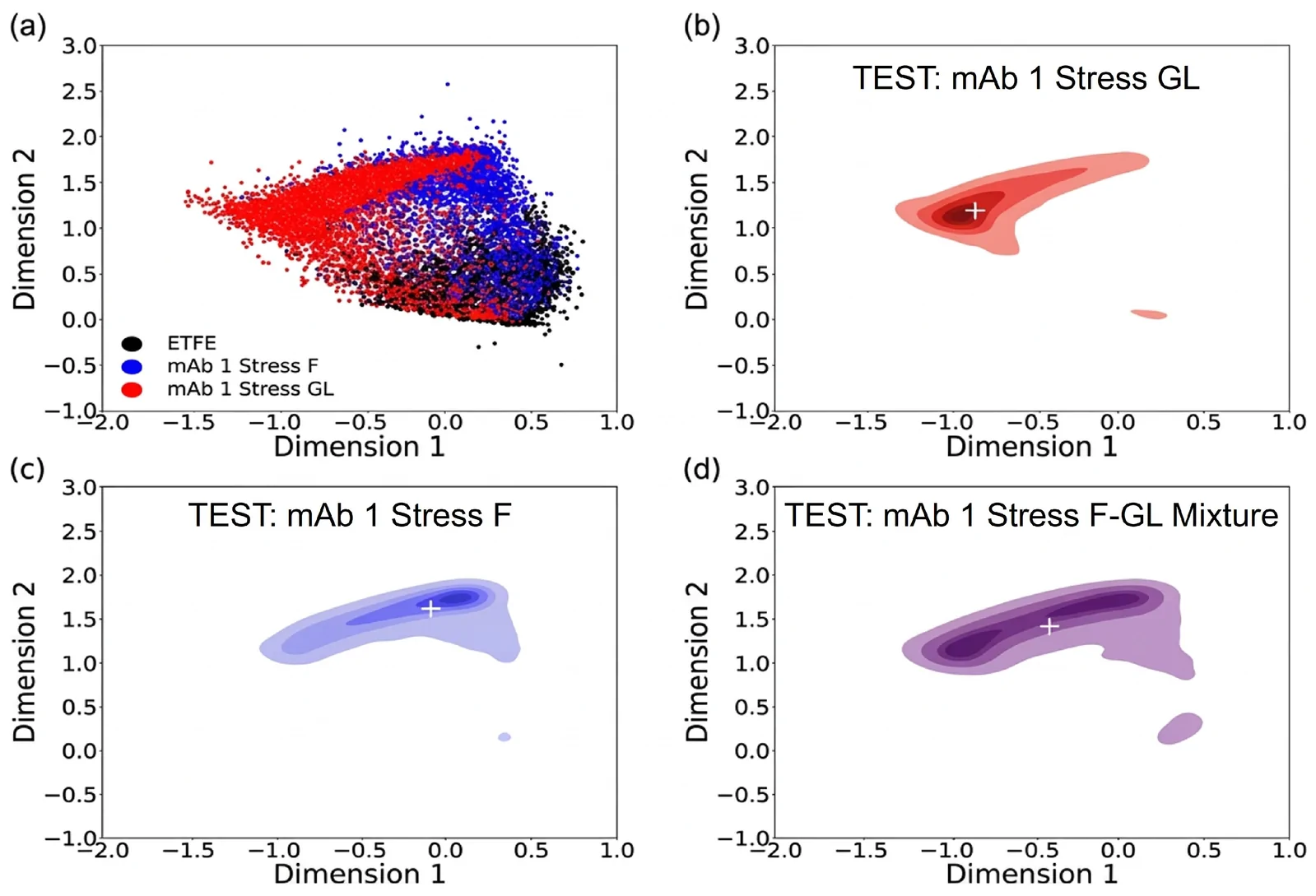
(**a**–**d**) Training and test clouds from a CNN trained on 16,000 images and 2 stresses, from [66].

A fingerprint CNN was trained on particle images captured after subjecting mAb 1 to Stress F, or Stress GL, or depicting ETFE particles. The trained CNN model was then validated vs. FIM test datasets, and Figure 59 shows embedding outputs for both stresses.

**Figure 60:**
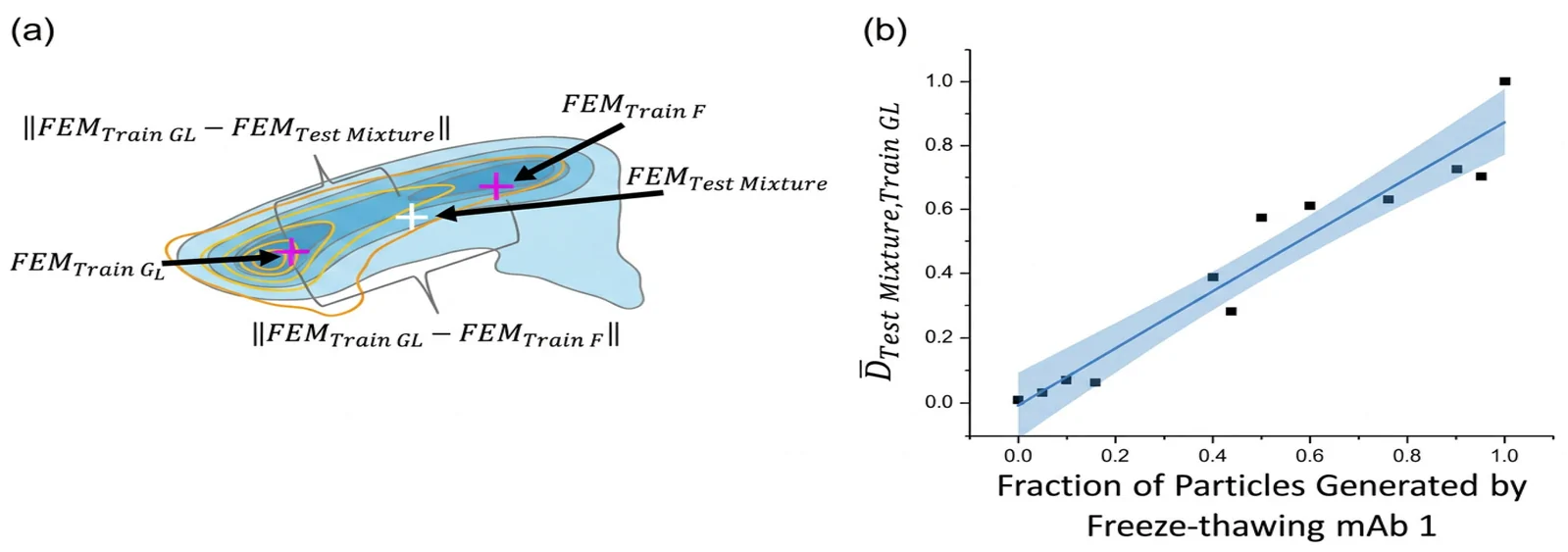
Test embedding fingerprint from an approximate 50–50% particle mixture, from [66].

Figure 60a shows a test embedding fingerprint from a 50–50% particle mixture which is generated by pumping mAb 1 via large-D Gore^®^ tubing (Stress GL) and freeze-thawing (Stress F) mAb 1 (green) with the training fingerprint by Stress GL (orange contours). Cross-hairs indicate FEMTest Mixture (white), FEMTrain F and FEMTrain GL (magenta). Euclidian distances between key FEM coordinate points are indicated with curly braces, and contours refer to 90%, 75%, 50%, 25%, and 10% of the embedding point cloud mass. Figure 60b is a scatterplot of D_Test Mixture, Train GL_ vs. the particle fraction generated by Stress F (the shaded area represents a 95% confidence interval on a linear fit of the relevant data).

**Figure 61:**
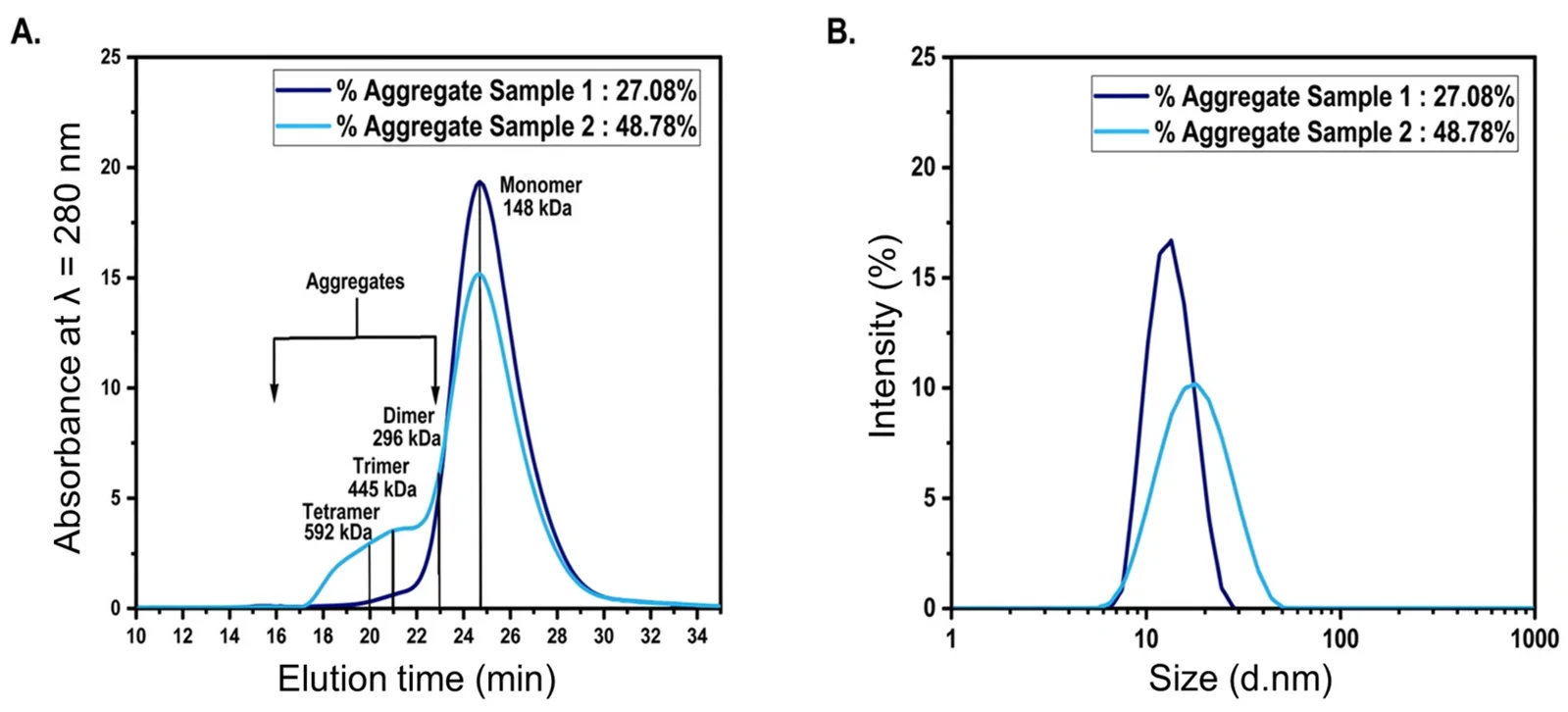
(**A**) SEC chromatogram, (**B**) DLS-PSD oligomer distribution analysis curves, from [68].

Beyond mAb properties (e.g., viscosity, thermal stability), a concern for industrial mAb therapeutics manufacturing is the detrimental agglomeration of many biomolecules; Shrivastava et al. (2023) [68] authored an ML-driven dynamic light scattering (DLS) study. DLS is a well-established method for evaluating sample stability and estimating average protein aggregate sizes by measuring size distributions in a wide (nano-to micro-) range, to compute relative percentages of multimers (monomers, dimers, trimers, tetramers) for two products (mAb1, mAb2), in the 10–100 nm range (e.g., size distributions in Figure 61).

Their experimental protocol entailed the manipulation of key operating conditions (e.g., pH, temperature, light intensity) for generating a wide variety of mAb aggregates. Light intensities of scattered (633 nm He–Ne) light from resulting mAb solutions were measured using a Zetasizer Nano ZS 90 instrument (Malvern Instruments, Malvern, UK). These DLS scattered intensities were recorded at a fixed scattering angle, and all samples were allowed to attain equilibrium before performing 11 scans for each measurement. Particle size distributions (PSD) were then calculated by post-processing the said DLS data, via proprietary Zetasizer software (Malvern Instruments, Malvern, UK) (Figure 61).

Thereafter, two ML algorithms were used for quantifying various mAb multimers. The first is an SVR/SVM method (a powerful SL tool), the other is an NN of multiple layers and neurons with adjustable weights; both algorithms were used to describe DLS data.

A total of 150 and 132 data points were used to study mAb1 and mAb2, respectively: 130 (mAb1) and 112 (mAb2) of them were used to train the models, while the remaining 20 (for both mAb cases) data points were used for the equally essential NN model testing. The goal is to quantify the composition (all multimer components) using these algorithms.

**Figure 62:**
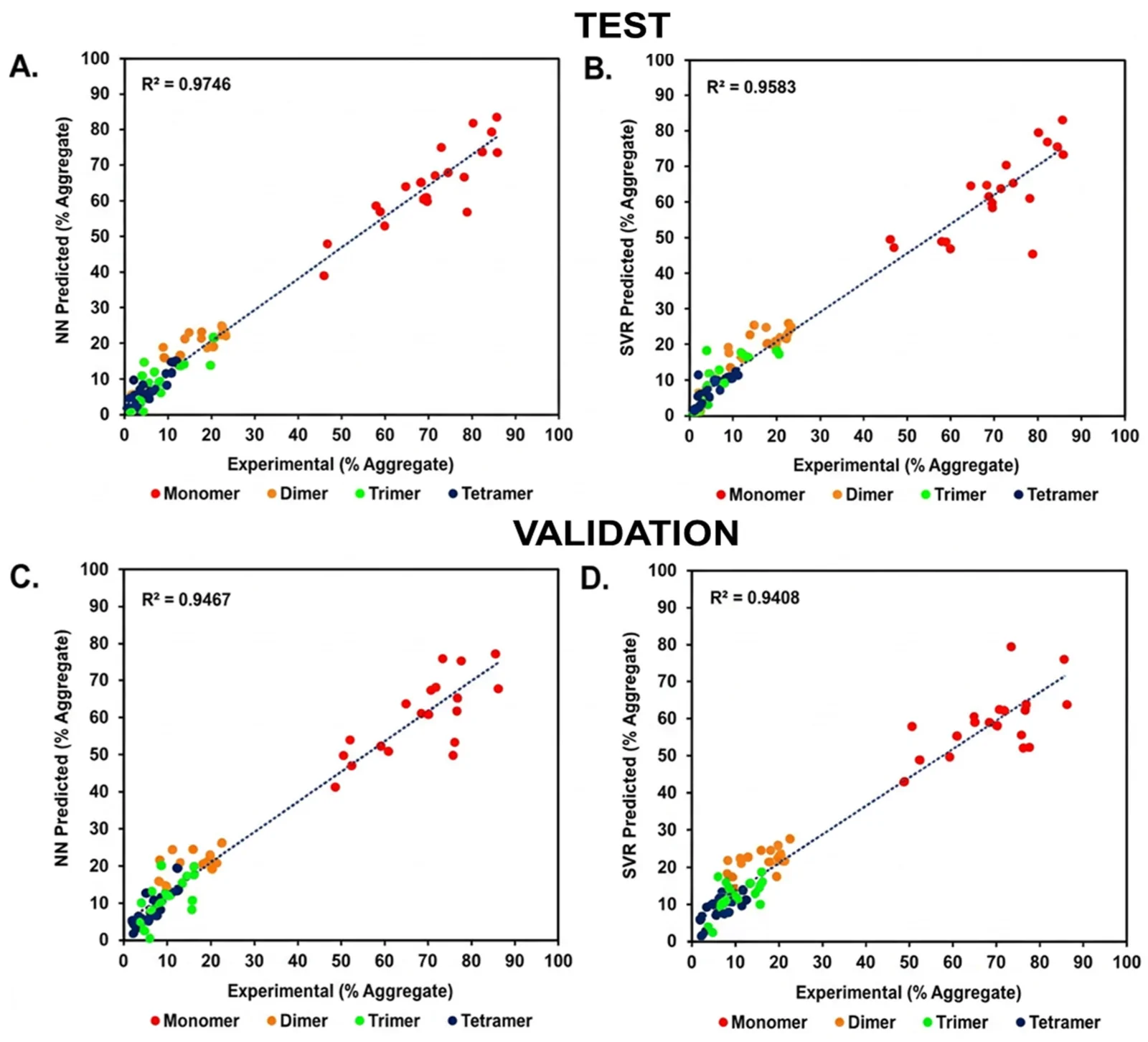
Predicted vs. exptl. data for mAb1 test and validation ((**A**,**C**): NN; (**B**,**D**): SVR) [68].

**Figure 63:**
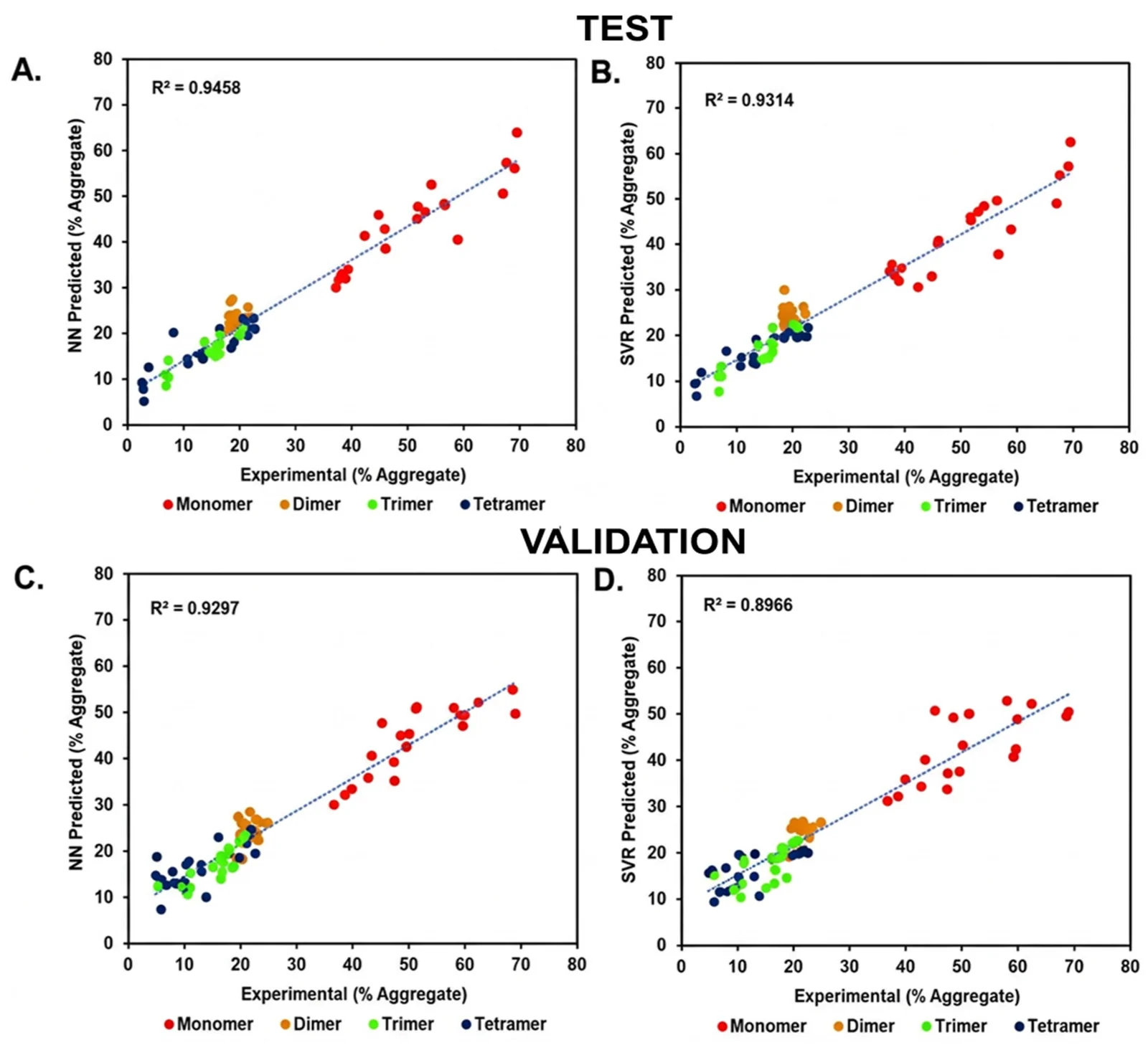
Predicted vs. exptl. data for mAb2 test and validation ((**A**,**C**): NN; (**B**,**D**): SVR) [68].

NN and SVR predictions for test and validation datasets are directly comparable for mAb1 and mAb2 products, respectively. For mAb1 test data, both algorithms predicted oligomer composition with determination coefficients (*R*^2^) of 0.97 (NN) and 0.96 (SVR). For mAb1 validation data, respective *R*^2^ values were 0.95 (NN) and 0.94 (SVR) (Figure 62). For mAb2 test data, oligomer prediction *R*^2^ values are 0.95 (NN) and 0.93 (SVR), but for mAb2 validation data, both R^2^ values are the lowest: 0.93 (NN) and 0.91 (SVR) (Figure 63).

The conclusion emerging from the said study is that DLS-ML (NN and SVR) results were in good agreement with mAb experimental datasets: the NN prediction algorithm achieved higher accuracy vs. the SVR model, for both the validation and the test datasets. The proposed ML approach was deemed to have great potential for application at various process R&D and analysis stages, for the given (mAb) and other therapeutic modalities. High-throughput mAb aggregation prediction at high concentration is also achieved [69].

**Figure 64:**
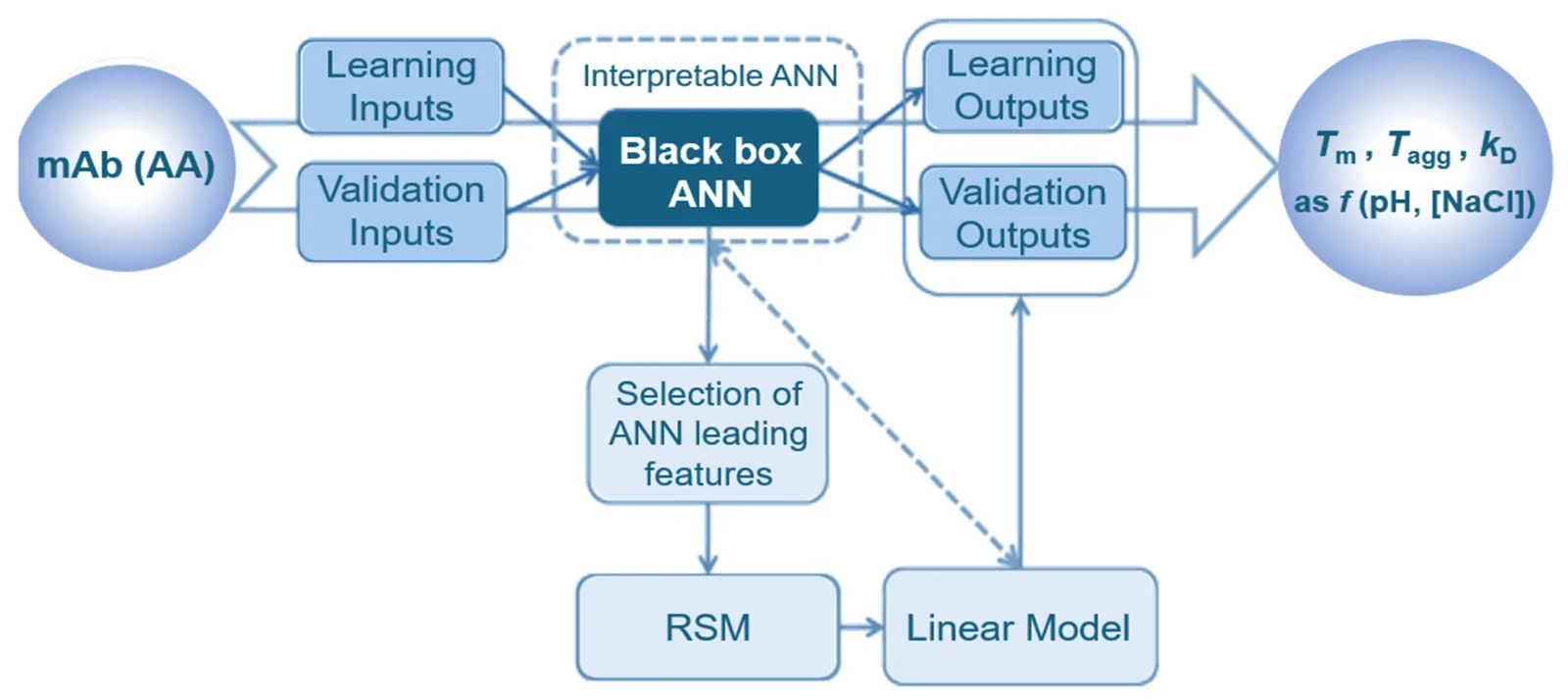
The ML implementation used to derive interpretable ANN-based predictions, from [70].

**Figure 65:**
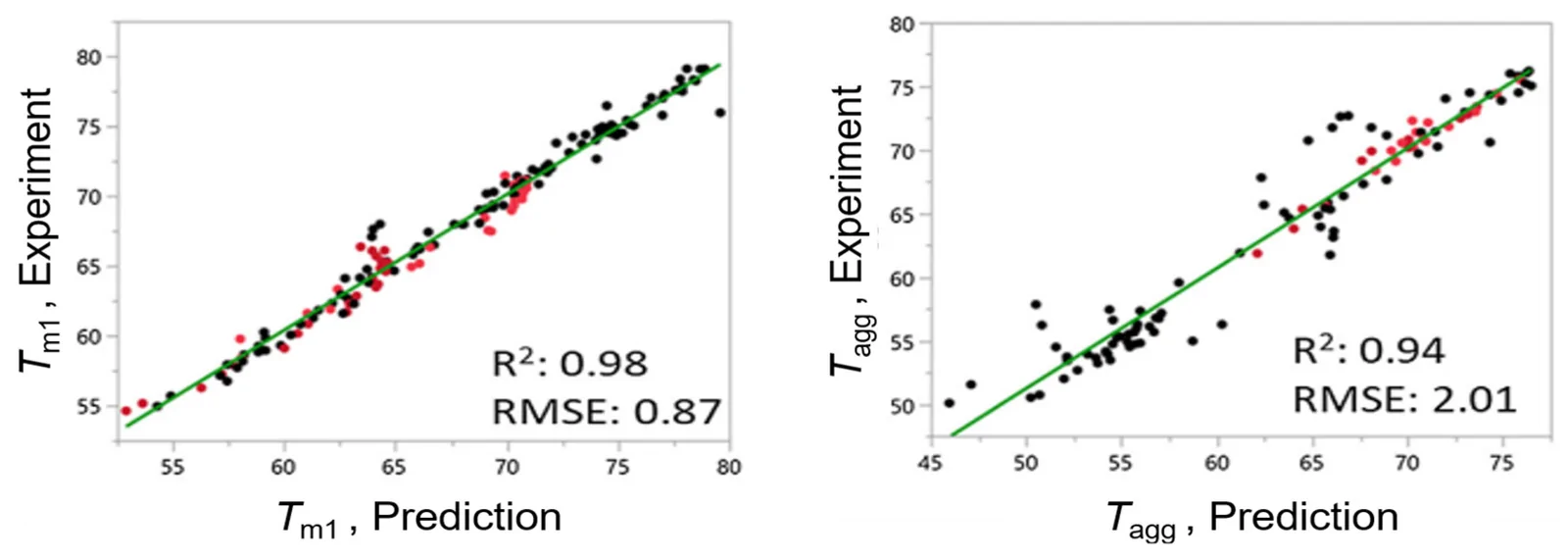
PPI-13&3 *T_m_*_1_ & *T_agg_* prediction performance (black: training, red: validation), from [70].

A study by Gentiluomo et al. (2019) [70] proposes interpretable ANNs for therapeutic mAb biophysical property prediction (melting temperature *T_m_*, aggregation onset temperature *T_agg_*, and interaction parameter *k_D_*) as a function of pH, salt concentration and amino acid composition (Figure 64). The authors used early-stage screening datasets for ANN training, with accuracy: Figure 65 shows ANN predictions vs. experimental data.

Model *T_m_*, *T_agg_* and *k_D_* predictions from mAb amino acid composition and the said formulation conditions (pH, salt concentration) were cross-validated (data for two mAbs were selected and held back from ANN training, for completing an unbiased validation). The higher RMSE for *T_agg_* vs. *T_m_* (2.01 vs. 0.87 °C) can be attributed to high-throughput screening, which stretched the required high data density for determination of the onset. The *k_D_* sign predictions are very good, without false negatives or false positives (Table 10).

## 7. Conclusions

Artificial Intelligence & Machine Learning (AI/ML) methods and software hold great promise in bioprocess R&D [71], with booming focus on biopharma mAb therapeutics. This review highlights pivotal ML applications in plantwide mAb modelling, bioreactor, downstream and portfolio optimisation, process control and property prediction [72,73,74,75], belabouring several AI/ML-driven protein structure-function mapping studies [76,77].

## Figures and Tables

**Table 1:** Final MLPCA model kinetic parameter values for rate expressions *φ* ^(i)^ and amino acids [6].

Parameter	Value	Unit	Parameter	Value	Unit
*φ*_max_ ^(1)^	17.166	d^−1^	*K*_N,s_	48.147	mM
*φ*_max_ ^(2)^	199.847	d^−1^	*K*_Asn,s_	0.135	mM
*φ*_max_ ^(3)^	0.0243	d^−1^	*K*_Arg,s_	67.467	mM
*K*_Cys,s_	0.099	mM	*K*_Ala,inh_	0.113	mM^−1^
*K*_Val,s_	0.288	mM	*K*_Met,inh_	6.179	mM^−1^
*K*_Ile,s_	0.294	mM	*K*_Pro,inh_	19.991	mM^−1^
*K*_Leu,s_	0.298	mM	*K*_Val,inh_	0.221	mM^−1^
*K*_Lys,s_	2.805	mM	(kinetic rate expressions *φ* ^(i)^ detailed in [3])		

**Table 2:** Experimental data-driven model fitting cost function *J_ML_* residuals in cross-validations [6].

Experiment	*J*_ML_ (*n* = 10^3^)	*J*_ML_ (*n* = 10^4^)
3	0.444	0.553
4	0.879	1.076
5	0.840	0.974

**Table 3:** ANN performance analysis: error metrics vs. experimental data for different periods [15].

Days	0–8	8–10	10–12	12+
Training Error (%)	0.14	0.4	0.27	0.07
Prediction Error (%)	0.11	0.22	0.13	0.03

**Table 4:** Final yield for each bioreactor and the two feeding regimes, in the validation study [22].

Condition	Replicate	Product Concentration (mg.L^−1^)
Control with non-uniform feeding	1	3635.036
Control with non-uniform feeding	2	2665.541
Optimised feed with non-uniform feeding	1	2825.806
Optimised feed with non-uniform feeding	2	2927.120
Optimised feed with uniform feeding	1	2828.525
Optimised feed with uniform feeding	2	2752.028

**Table 5:** Misclassification (*R^clf^*), mean abs. relative (*R^reg^*) and combined error (*R^comb^*) scores [29].

	Linear (Chemometrics)			Machine Learning		
Molecule	*R^clf^* (%)	*R^reg^* (%)	*R^comb^* (%)	*R^clf^* (%)	*R^reg^* (%)	*R^comb^* (%)
**Infliximab**	13.7	12.3	**13.2**	0.9	8.4	**3.4**
**Bevacizumab**	19.8	14.0	**17.9**	1.0	4.3	**2.1**
**Ramucirumab**	9.0	7.3	**8.4**	0.0	3.5	**1.2**
**Rituximab**	16.0	26.7	**19.6**	1.0	6.9	**3.0**
**Overall**	14.5	14.7	**14.6**	0.7	5.8	**2.4**

**Table 6:** RSM-ANN training and testing for IgM mAb production by hybridoma M1A2 cells [36].

Dataset Type and Medium Type	FBS (%)	Cultivation Time (day)	Temperature (°C)	Experimental mAb (μg/mL)	Predicted mAb (μg/mL)
Training data	5	3	33	673.97	673.88
(DMEM)	7	5	33	719.24	719.34
	7	2	33	638.02	638.08
	20	3	33	821.39	821.37
	17	2	33	509.83	509.88
	**17**	**5**	**33**	**1220.0**	**1219.8**
	5	3	37	529.14	529.14
	10	4	37	603.78	607.98
	10	4	37	610.00	605.77
	10	4	33	1066.7	1033.8
	10	4	33	1006.0	1039.5
	17	5	37	600.00	600.04
	10	6	37	600.00	599.98
	10	1	33	411.87	411.78
	20	3	37	529.14	529.15
	10	6	33	1097.2	1097.1
Testing data	10	4	37	610.00	608.00
(DMEM)	**10**	**4**	**33**	**1050.0**	**1034.0**
	7	2	37	504.31	545.62
	7	5	37	569.24	585.93
	10	1	33	400.00	411.72
Training data	5	3	33	354.06	354.06
(RPMI 1640)	7	5	33	600.00	600.00
	7	2	33	446.90	446.90
	20	3	33	125.29	125.28
	17	2	33	194.82	194.81
	17	5	37	568.18	568.17
	5	3	37	289.52	289.52
	10	4	37	547.45	547.45
	10	4	33	520.00	520.00
	**10**	**4**	**37**	**1100.0**	**1100.0**
	17	5	37	120.00	120.00
	10	6	37	100.00	100.00
	10	1	33	332.01	332.00
Testing data	10	4	37	520.00	533.73
(RPMI 1640)	**10**	**4**	**33**	**1002.0**	**1100.0**
	7	2	37	451.42	504.14
	7	5	37	110.00	213.54
	10	1	33	153.76	243.23

**Table 7:** Summary of ML algorithms and results of optimisation studies, and authoritative reviews.

Authors	Year	ML Method	Effectiveness	Remarks
Dewasme et al. [5]	2017	Principal Component Analysis (PCA), and MATLAB 2025a (*fmincon*)	30% increased production	Final mAb titre: 60.92 µg.mL^−1^ vs. 40 to 45 µg.mL^−1^
Le et al. [29]	2018	Kernel PCA and Support Vector Machines (SVM)	97.6% prediction accuracy	Combined error of 2.4% vs. 14.6% by a linear approach
Dewasme et al. [6]	2023	Max. Likelihood Principal Component Analysis (MLPCA) and Multi-Stage Nonlinear Model Predictive Control (MSNMPC)	28% increased production	Final mAb titre: 17.1 mM vs. 12.5 mM by NMPC
Manapragada et al. [22]	2023	Input–Throughput– Output (ITO) dual ANN model	8.9% increased production	Product titre via feed optimisation: 2927 vs. 2665 mg.L^−1^
Pham et al. [3]	2023	Supervised Learning (SL) & data-driven optimisation (SLDDO) use in studies	-	(Review paper)
Rathore et al. [10]	2023	A review of several AI/ML applications in the modern biopharmaceutical industry	-	(Review paper)

**Table 8:** Performance of both PLS and JY-PLS models in estimating the number of viable cells [27].

Scale (Figure 46)	Number of Available Clones	*R* ^2^_PLS_	*R* ^2^_JY-PLS_	e_JY-PLS_ < e_PLS_ (%)
24 w	277	0.99	0.98	35
24 w L	195	0.96	0.88	28
6 w	82	0.99	0.98	45
6 w L	42	0.94	0.95	40
T25	48	0.96	0.95	52
T25 L	17	0.86	0.86	52

**Table 9:** Manufacturing stress condition abbreviations relevant to FIM images of Figure 58 [66].

Stress	Abbreviation
Freeze–thaw	Stress F
Agitation	Stress A
Pumping in Gore^®^ large diameter tubing	Stress GL
Pumping in Gore^®^ small diameter tubing	Stress GS
Pumping in C-flex^®^ ULTRA tubing	Stress C
pH swing	Stress P

**Table 10:** Validation set results for the predicted vs. true sign of interaction parameter *k*_D_ [70].

	Predicted Sign of *k*_D_	
Sign of *k*_D_ Count:	Negative	Positive
**Negative**	48 **+ 24**	0
**Positive**	0	24 **+ 12**

## Data Availability

No new data were created or analyzed in this study. Data sharing is not applicable to this article, and Literature References provide more details on all topics discussed.
